# Functional, chemical genomic, and super-enhancer screening identify sensitivity to cyclin D1/CDK4 pathway inhibition in Ewing sarcoma

**DOI:** 10.18632/oncotarget.4903

**Published:** 2015-08-18

**Authors:** Alyssa L. Kennedy, Mounica Vallurupalli, Liying Chen, Brian Crompton, Glenn Cowley, Francisca Vazquez, Barbara A. Weir, Aviad Tsherniak, Sudha Parasuraman, Sunkyu Kim, Gabriela Alexe, Kimberly Stegmaier

**Affiliations:** ^1^ Department of Pediatric Oncology, Dana-Farber Cancer Institute and Boston Children's Hospital, Boston, Massachusetts, USA; ^2^ Boston Combined Residency Program in Pediatrics, Boston, Massachusetts, USA; ^3^ Department of Internal Medicine, Brigham and Women's Hospital, Boston, Massachusetts, USA; ^4^ Broad Institute, Cambridge, Massachusetts, USA; ^5^ Novartis Institute for Biomedical Research, Cambridge, Massachusetts, USA; ^6^ Bioinformatics Graduate Program, Boston University, Boston, Massachusetts, USA

**Keywords:** CDK4/6 inhibitor, cyclin D1, epigenetics, Ewing sarcoma, sarcoma/soft-tissue malignancies

## Abstract

Ewing sarcoma is an aggressive bone and soft tissue tumor in children and adolescents, with treatment remaining a clinical challenge. This disease is mediated by somatic chromosomal translocations of the *EWS* gene and a gene encoding an ETS transcription factor, most commonly, *FLI1*. While direct targeting of aberrant transcription factors remains a pharmacological challenge, identification of dependencies incurred by EWS/FLI1 expression would offer a new therapeutic avenue. We used a combination of super-enhancer profiling, near-whole genome shRNA-based and small-molecule screening to identify cyclin D1 and CDK4 as Ewing sarcoma-selective dependencies. We revealed that super-enhancers mark Ewing sarcoma specific expression signatures and EWS/FLI1 target genes in human Ewing sarcoma cell lines. Particularly, a super-enhancer regulates cyclin D1 and promotes its expression in Ewing sarcoma. We demonstrated that Ewing sarcoma cells require CDK4 and cyclin D1 for survival and anchorage-independent growth. Additionally, pharmacologic inhibition of CDK4 with selective CDK4/6 inhibitors led to cytostasis and cell death of Ewing sarcoma cell lines *in vitro* and growth delay in an *in vivo* Ewing sarcoma xenograft model. These results demonstrated a dependency in Ewing sarcoma on CDK4 and cyclin D1 and support exploration of CDK4/6 inhibitors as a therapeutic approach for patients with this disease.

## INTRODUCTION

Ewing sarcoma is an aggressive solid tumor affecting children and young adults. Effective treatment options for patients with metastatic and recurrent disease remain inadequate [1, 2]. The primary known oncogenic event in Ewing sarcoma is a somatic chromosomal translocation, most commonly between chromosomes 11 and 22, which results in a fusion between the 5′ region of the *EWS* gene and the 3′ region of the ETS family gene, *FLI1* [3]. The resulting fusion protein, EWS/FLI1, retains DNA-binding activity and promotes the expression of an aberrant transcriptional profile that is oncogenic in a permissive cell context [4–6]. While EWS/FLI1 plays a central role in orchestrating expression of oncogenic mediators in Ewing sarcoma, it remains a challenging drug target [7]. A compelling alternative approach to the development of effective targeted therapies in Ewing sarcoma is to identify Ewing sarcoma selective dependencies, such as the cooperating oncogenic pathways that are regulated by EWS/FLI1 expression or the epigenetic profiles that mediate tumorigenesis and proliferation.

With the characterization of the genomic landscapes of tumors, it has become clear that there is activation of oncogenic drivers, mutations in tumor suppressors, as well as epigenetic changes that contribute to the hallmarks of tumor cells [8]. Interestingly, the Ewing sarcoma cancer genome is characterized by one of the lowest mutational rates amongst cancer types [9–12], implicating epigenetic deregulation as a possible component of tumor development. A better understanding of epigenetic control of gene expression has begun to provide mechanistic insight into the complex regulatory elements that promote both normal and tumor cell identity and proliferation alike [13]. Recently, it has been shown that EWS/FLI1 utilizes divergent chromatin remodeling mechanisms to directly activate or repress enhancer elements in Ewing sarcoma [14, 15]. In the current study we focused on the importance of distal regulatory elements, in particular “super-enhancers,” in marking a small number of expressed genes that are essential for cell fate and identity in Ewing sarcoma. Super-enhancer regions of chromatin are broad regions of open chromatin with acetylated histones, master transcription factors and transcriptional activators [16, 17]. These regions can form loops to approximate the enhancer region with genes nearby to promote transcription. It has become increasingly clear that super-enhancer regions can be corrupted in cancer cells where they mark critical oncogenic drivers and are bound by tumor-specific master transcription factors that mediate a tumor-specific gene expression program [18, 19].

While some super-enhancer regions in cancer cells may mark genes that promote the malignancy, others may mark genes that are not essential to the cell. Intersection of epigenetic profiling with other high-throughput screening approaches may enable the prioritization of potential oncogenes. The last decade has seen a marked increase in the development and implementation of high-throughput approaches for the discovery of new targets in cancer. For example, RNAi-mediated functional genomic screening, and more recently CRISPR/Cas9 screening, provide powerful tools for high-throughput assessment of gene dependencies in mammalian systems. Similarly, more widespread access to small-molecule library screening capabilities has advanced discovery of new tool compounds for cancer research application. There still remain challenges to each of these screening modalities, however, such as off-target effects leading to false positives and false negatives. Integrated approaches that incorporate epigenetic, genetic, and small-molecule screening data now allow for the nomination of higher confidence candidate targets.

Toward this end, we integrated the results of super-enhancer profiling, a near-whole genome shRNA screen, and a publically available chemical screening database to identify a dependency of Ewing sarcoma cells on the G1 cell cycle signaling proteins cyclin D1 and CDK4. We also determined that the cyclin D1 gene (*CCND1)* is regulated by a super-enhancer and confirmed Ewing sarcoma is selectively dependent on *CCND1* and *CDK4* compared to other cancer cell lines. In addition, we showed that Ewing sarcoma cell lines are sensitive to the pharmacological inhibition of CDK4/6, both *in vitro* and *in vivo*. These studies support the exploration of CDK4/6 inhibitors as a treatment strategy in patients with Ewing sarcoma.

## RESULTS

### Super-enhancer screening identifies Ewing sarcoma oncogenic transcriptional targets

Recent work describing the Ewing sarcoma cancer genome has demonstrated that Ewing tumors have very few driver mutations other than the pathognomonic *EWS/ETS* somatic translocations [10–12]. Therefore, we hypothesized that epigenetic contributions to tumor initiation and maintenance may be especially important in Ewing tumors. We performed super-enhancer profiling to identify critical, and possibly targetable, dependencies that would not be apparent by traditional genomic sequencing.

**Figure 1:**
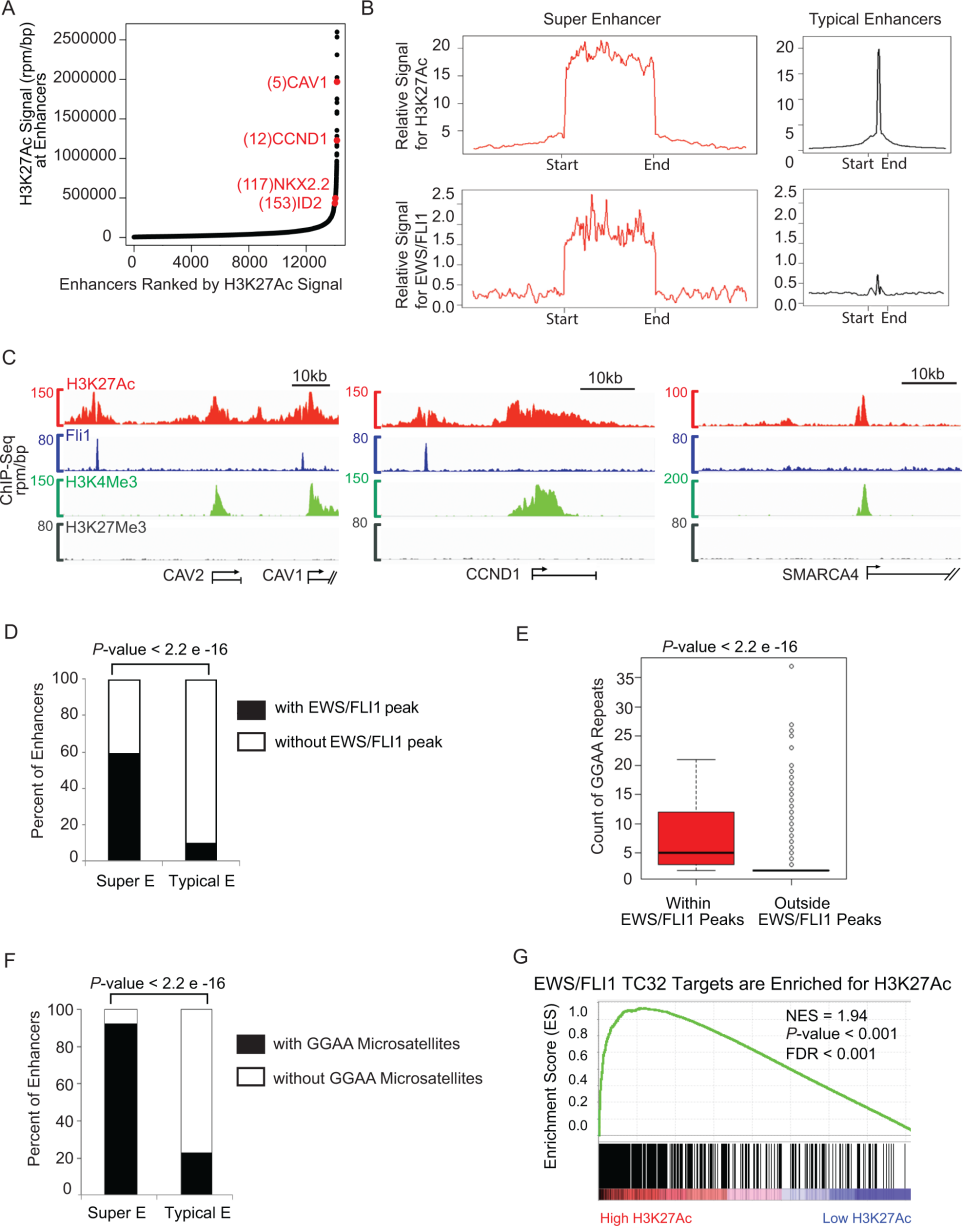
Super-enhancer profiling in TC32 identifies CCND1 and other important EWS/FLI1 targets **A.** Total H3K27Ac ChIP-seq signal in units of reads per million in enhancer regions for all enhancers in TC32. Enhancers are ranked by increasing H3K27Ac ChIP-seq signal. **B.** Metagene representation of global H3K27Ac or EWS/FLI1 occupancy at typical enhancers and super-enhancers in TC32. Metagenes are centered on the enhancer region, and the length of the enhancer reflects the difference in median length (2 kb for typical enhancers, 33 kb for super-enhancers). Additional 5 kb surrounding each enhancer is also shown. The y axis shows the relative ChIP-seq signal. **C.** Gene track of H3K27Ac (red), and FLI1 (blue), H3K4Me3 (green), H3K27Me3 (grey). ChIP-seq occupancy at the super-enhancer regions surrounding *CAV1/2* and *CCND1*, and at the typical enhancer region upstream of *SMARCA4*. **D.** Fisher test showed super-enhancer regions have significantly more EWS/FLI1 peaks than typical enhancer regions (odd ratio =15.8, *P*-value < 2.2 x e-16.) **E.** Two tailed *t*-test showed that the GGAA microsatellites in EWS/FLI1 peaks have more GGAA repeat counts than those in other genome regions outside of the EWS/FLI1 peaks (average GGAA count in EWS/FLI1 peaks = 7.3, average GGAA count outside EWS/FLI1 peaks = 2.3, *P*-value < 2.2 e-16.) **F.** Fisher test showed super-enhancer regions have significantly more GGAA microsatellites than typical enhancer regions (odd ratio = 38.0, *P*-value < 2.2e-16.) G. GSEA demonstrates enrichment of H3K27Ac binding in EWS/FLI1 target genes.

To identify active promoter and enhancer elements in Ewing sarcoma and determine global binding of the oncogenic transcription factor EWS/FLI1, we performed chromatin immunoprecipitation coupled to high-throughput sequencing (ChIP-seq) in two Ewing sarcoma cell lines, TC32 and TC71. Because the wild type FLI1 protein is not expressed in Ewing tumors [20], EWS/FLI1 binding was assayed with an antibody that recognizes the endogenous FLI1 peptide sequence. Enhancer regions were identified by analyzing the presence of the histone mark H3K27Ac. Because this histone mark is also present at the transcription start site (TSS), while H3K4me3 only annotates the TSS, we identified enhancer regions based on the presence of H3K27Ac signal outside of the TSS and without presence of H3K4me3, similar to methods and algorithms described in prior studies [16, 19]. We then ranked all of these defined enhancers based on increasing H3K27Ac ChIP-seq signal (Figure 1A and Supplementary Figure S1A). Among the collection of more than 14,000 enhancers in the genome, more than half of the H3K27Ac signal was deposited on the 250 top-ranking enhancers, which we refer to subsequently herein as super-enhancers. Notably, in the Ewing sarcoma cell lines profiled, typical enhancers had a median size range of 1.5 to 2.0 kb, while the top super-enhancers had a median size range of 28 to 33 kb based on H3K27Ac binding peaks (Supplementary Figure S1B).

Enhancers typically associate with target genes via looping within a distance of approximately 50 kb. We therefore nominated super-enhancer-associated genes by assigning all transcriptionally active genes to super-enhancers within a 50 kb window [19, 21]. We identified 321 and 239 genes associated with the 250 super-enhancers in TC32 and the 138 super-enhancers in TC71, respectively (Supplementary Tables S1 and S2). Many of the top ranked super-enhancer associated genes were genes of known relevance in Ewing sarcoma, including *caveolin 1 (CAV1)*, *NKX2.2*, *ID2* and *CCND1* [20, 22–25].

During normal development and in the malignant state, super-enhancer regions are often associated with enrichment of master transcription factors. Therefore, to compare the genome-wide binding patterns of the oncogenic transcription factor EWS/FLI1 and H3K27Ac at typical enhancers versus super- enhancer regions we performed metagene analysis. This analysis is used to compare the aggregated binding of a protein of interest centered at a specific locus. It showed that the average ChIP-seq signal of EWS/FLI1 and H3K27Ac spanning and flanking the super-enhancers was considerably higher as compared to typical enhancers in TC32 and TC71 Ewing sarcoma cells (Figure 1B and Supplementary Figure S1C, respectively).

We then examined the individual gene tracks of two of the largest super-enhancers in Ewing sarcoma, which were associated with *CAV1/2* and *CCND1* (Figure 1C). The amounts of an active mark H3K4me3 and a repressive mark H3K27me3 in the gene body of *CAV1/2* and *CCND1* are comparable to those in the gene body of a typical enhancer-associated gene *SMARCA4*. However, the super-enhancers of *CAV1/2* and *CCND1* showed broad clusters of H3K27Ac binding regions as expected, but also showed centrally located EWS/FLI1 binding peaks. In contrast, the typical enhancer-associated gene *SMARCA4* had much less H3K27Ac signal and very low EWS/FLI1 signal in its enhancer region.

### Super-enhancers are enriched for GGAA microsatellites in Ewing sarcoma

We then performed a Fisher exact test to estimate the percentage of super-enhancer regions and typical-enhancer regions containing EWS/FLI1 binding peaks, further confirming that super-enhancer regions are significantly enriched for EWS/FLI1 binding (Figure 1D, odds ratio = 15.72, Fisher exact test *P*-value < 2.2e-16). Consistent with previous literature showing that GGAA microsatellites are a DNA binding motif of EWS/FLI1 [26], we found that GGAA microsatellites within EWS/FLI1 peaks have more GGAA repeat counts than those residing in regions outside of the EWS/FLI1 peaks (Figure 1E). Furthermore, in light of a recent paper reporting a role for EWS/FLI1 as a pioneer factor at enhancer elements [14], we hypothesized that super-enhancers would be enriched with GGAA microsatellites. We compared the distributions of GGAA microsatellites within super-enhancers and typical enhancers in the Ewing genome and found that the percentage of super-enhancers containing GGAA microsatellites is significantly higher than the percentage of typical-enhancers containing GGAA microsatellites (Figure 1F).

We next used Gene Set Enrichment Analysis (GSEA) [27, 28] to estimate the significance of coordinated changes in two data sets, comparing EWS/FLI1 target genes and those with high levels of H3K27Ac. We found a significant enrichment of our ChIP-seq-defined EWS/FLI1 target genes in the category of genes with high levels of H3K27Ac binding signal in their enhancer regions (Figure 1G and Supplementary Figure S1D), consistent with enrichment of this oncogenic transcription factor at super-enhancers in Ewing sarcoma.

### Super-enhancer target genes correlate with known expression signatures in Ewing sarcoma

**Figure 2:**
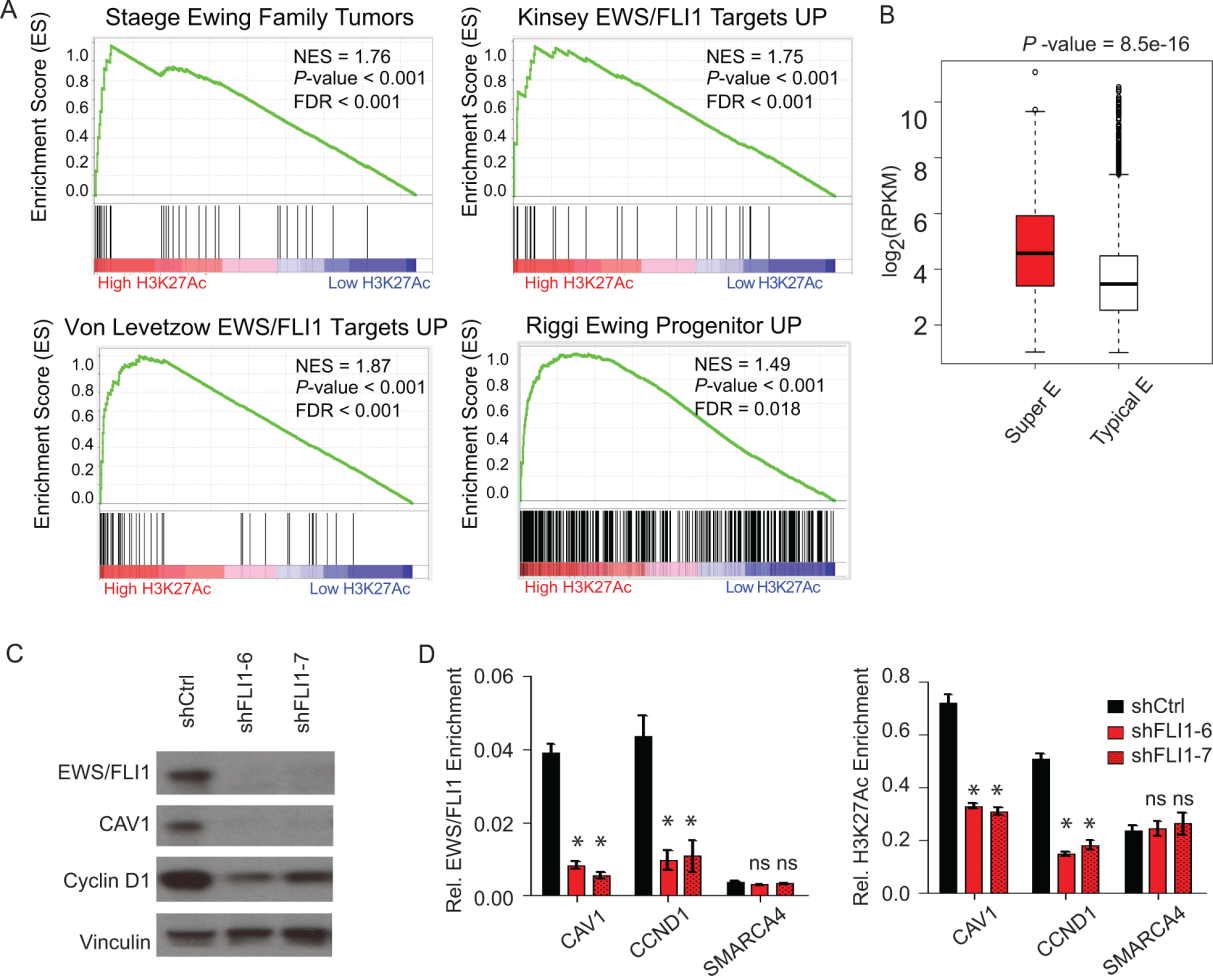
Super-enhancers play important role in Ewing sarcoma biology **A.** GSEA demonstrates that the Ewing family tumor gene signature and the various EWS/FLI1 target signatures are all enriched in genes with extremely high binding of H3K27Ac in the enhancer region. **B.** Box plots of expression values in TC32 cells for genes with proximal typical enhancers (white) or with proximal super-enhancers (red). **C.** mir30-shRNA-mediated knockdown of EWS/FLI1 in the TC32 cell line reduced the protein levels of the Ewing super-enhancer-associated genes *CAV1* and *CCND1*, with vinculin as a control. **D.** ChIP-qPCR analysis showing that conditional EWS/FLI1 knockdown in TC32 cells reduced the relative enrichment of EWS/FLI1 and H3K27Ac in the super-enhancer regions (*CAV1*, *CCND1*) but not in the typical enhancer region (*SMARCA4*). *P-value < 0.01, ns: not significant.

To further assess the extent to which H3K27Ac super-enhancer genes are associated with the tumor cell state in Ewing sarcoma, we performed GSEA comparing the list of H3K27Ac marked genes with a collection of publicly available Ewing sarcoma signatures from previously published studies. These gene sets were derived from global gene expression profiling comparisons between Ewing family tumors and normal tissues [29], before and after EWS/FLI1-induced expression in either neural crest cells [6] or mesenchymal stem cells [5], or before and after RNAi-mediated EWS/FLI1 knockdown in Ewing sarcoma cells [30]. GSEA revealed that the Ewing family tumor gene signature and the various EWS/FLI1 target signatures are all enriched in genes with high binding of H3K27Ac in the enhancer region (Figure 2A).

To further verify whether super-enhancer marked genes are associated with Ewing sarcoma gene signature pathways, we applied a complementary, unbiased enrichment method. We compared the lists of H3K27Ac marked genes in the TC32 and TC71 cell lines with the c2 collection of 4,722 canonical pathways and experimental gene sets available in MSigDB v4.0 [27]. Notably, many Ewing sarcoma-related oncogenic signatures emerged as top hits. (Supplementary Table S3) [5]. These results further support the notion that super-enhancers in Ewing sarcoma are associated with genes that regulate and enforce the Ewing sarcoma cancer cell state.

### Functional attributes of super-enhancers

We hypothesized that Ewing specific dependency genes may rely more heavily on super-enhancers to regulate gene transcription than those genes marked with typical enhancers. To address this question, we evaluated Ewing sarcoma RNA-sequencing data derived from Ewing sarcoma cell lines and primary patient tumors [11]. We found that genes associated with super-enhancers are generally more highly expressed than genes associated with typical enhancers (Figure 2B). These results are consistent with the concept that super-enhancers enable high levels of transcription of key genes that regulate and likely re-enforce the Ewing sarcoma cancer cell state.

A recent study depicting mechanisms of EWS/FLI1 chromatin remodeling has shown that EWS/FLI1 enforces an oncogenic regulatory program through activation of enhancer elements or repression depending on the genomic context [14]. In order to study the role of the EWS/FLI1 oncogenic transcription factor in maintaining super-enhancers specifically in Ewing sarcoma, we set up a Mir30-shRNA-mediated doxycycline-inducible EWS/FLI1 knockdown system in the Ewing sarcoma cell line TC32. In this system, EWS/FLI1 expression is significantly decreased after four days of doxycycline treatment. The suppression of EWS/FLI1 also leads to a selective decrease in protein expression of the Ewing sarcoma super-enhancer-associated genes *CAV1* and *CCND1* versus a vinculin control (Figure 2C). We subsequently assayed binding of EWS/FLI1 or H3K27Ac by ChIP-qPCR. EWS/FLI1 knockdown significantly reduced the EWS/FLI1 binding, as well as the H3K27Ac, at super-enhancer marked genes *CAV1* and *CCND1*, without significant effects on the *SMARCA4* typical enhancer (Figure 2D). These results suggest a role for EWS/FLI1 in maintaining both the function and the structure of the super-enhancers in Ewing sarcoma.

### Intersection of epigenetic screening with other screening methodologies identifies activation of the cyclin D1/CDK4 pathway in Ewing sarcoma

**Figure 3:**
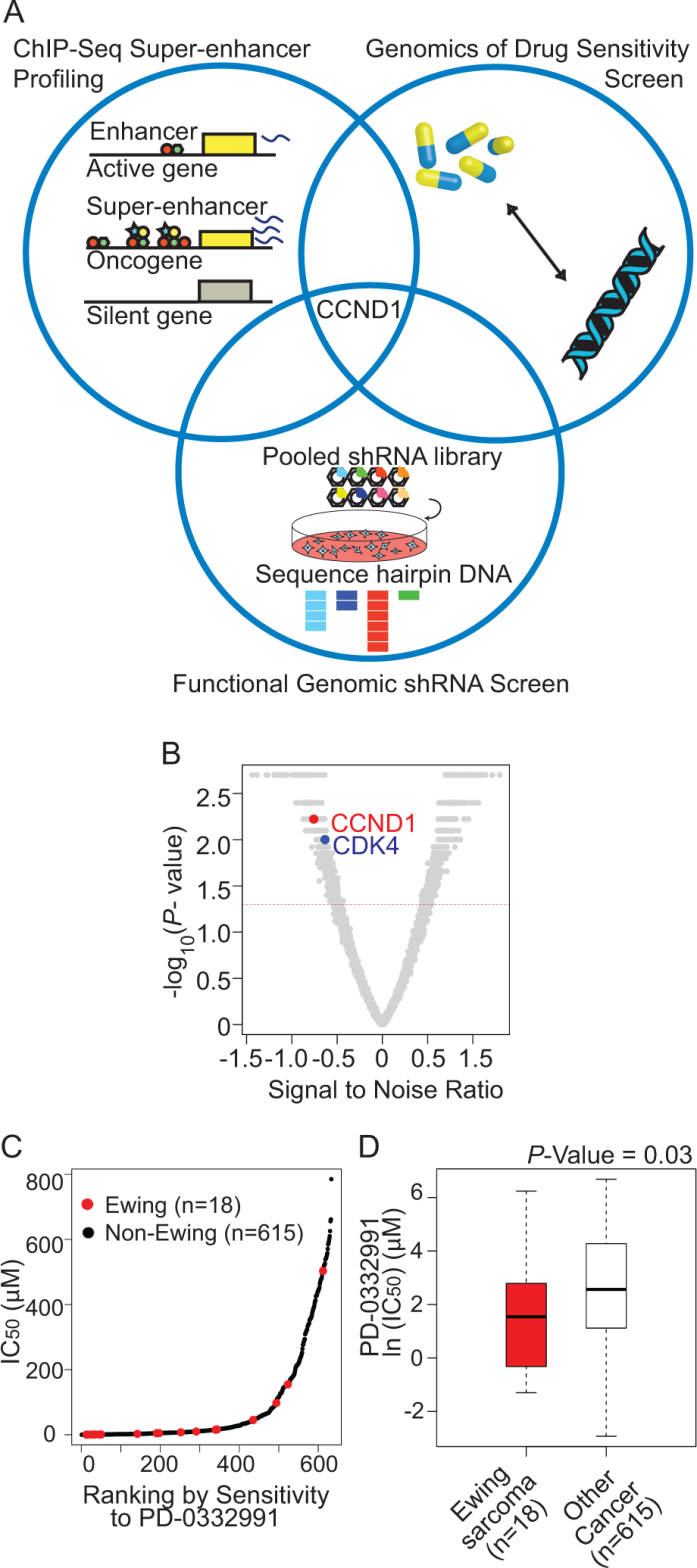
An integrative approach to identify Ewing sarcoma specific dependencies **A.** Venn diagram representing confluence of screening approaches that nominate the CDK4/cyclin D1 pathway as dependencies in Ewing sarcoma. **B.** Volcano plot depicting CCND1 and CDK4 as significant ATARiS depletion solutions for Ewing sarcoma compared to other cancer cell lines. Gene ranks based on signal to noise ratio (with significant *P*-value < 0.05), ranking: CCND1: 96, CDK4: 191 out of 4997 ATARiS solutions. **C.** Ranking of cell line data from Genomics of Drug Sensitivity in Cancer data: IC50 versus sensitivity to PD0332991. **D.** Cells expressing EWS/FLI1 have increased sensitivity (*P*-value = 0.03, Mann-Whitney test) to the CDK4/6 inhibitor PD0332991.

While some of the super-enhancer marked genes are likely to be oncogenic, it is unlikely that all of these genes will be relevant to tumor dependency. Thus, because our goal was to nominate targetable Ewing sarcoma dependencies, we intersected our list of super-enhancer marked genes with a near-whole genome shRNA screen and publically available data from a small-molecule screen of genomically characterized cancer cell lines [31] (Figure 3A). The shRNA screen was performed in 216 cell lines from 22 cancer types, with an shRNA library of 54,020 barcoded shRNAs in lentiviral vectors targeting 11,194 genes [32]. Four *EWS/FLI1*-rearranged Ewing sarcoma cell lines (A673, EW8, EWS502, and TC71) and one *EWS/ERG*-rearranged cell line (CADO-ES-1) were included in the screen. Ewing sarcoma dependencies were identified based on the Analytic Technique for Assessment of RNAi by Similarity (ATARiS) [33] (Figure 3B and Supplementary Table S4). The convergence of the super-enhancer marked genes and the shRNA screenings in TC32 and TC71 cells nominated *CCND1*, glycogen synthase kinase 3 alpha (*GSK-3α*) and microtubule-associated protein tau (*MAPT*). GSK-3α and the highly homologous GSK-3β, are serine threonine kinases which regulate numerous signaling pathways. MAPT is a gene that is alternatively spliced to create the tau proteins, which are implicated in Alzheimer's disease, neural crest development and neuronal differentiation. These tau proteins have also been previously identified to be important markers of neuronal features in gene signatures created from human mesenchymal stem cells expressing EWS/FLI1 [5].

To evaluate these candidates for possible therapeutic relevance, we queried small molecules targeting these candidate genes by referencing the Genomics of Drug Sensitivity in Cancer database [31]. In this data set, 634 cancer cell lines were treated with small molecules and the effects on cell viability measured. Using this data set, we queried whether the response of Ewing sarcoma cell lines to select inhibitors differed from that of the other cancer cell lines. We first evaluated response to GSK-3 inhibitors. While there were no GSK-3α isoform-selective inhibitors in the data set, we did evaluate response to the pan-GSK-3 inhibitors, including SB216763 and CHIR-99021. The IC_50_ values for these GSK-3 inhibitors were not significantly different in Ewing sarcoma cell lines as compared to other cancer types (Supplementary Figure S2). PD0332991, a well-validated small-molecule inhibitor of CDK4/6, and thus an indirect inhibitor of activated cyclin D1, was also profiled in the data set. The IC_50_ of PD0332991 in the Ewing cell lines was significantly lower than that of the other cancer cell lines tested in this dataset. In addition, the expression of EWS/FLI1 in Ewing sarcoma was a significant marker of increased drug sensitivity (*P*-value = 0.0307, Mann-Whitney test, Figure 3C and 3D). Unfortunately, there were no available tau modulators in this data set.

### CCND1 and CDK4 are highly expressed in Ewing sarcoma cell lines and primary tumors

**Figure 4:**
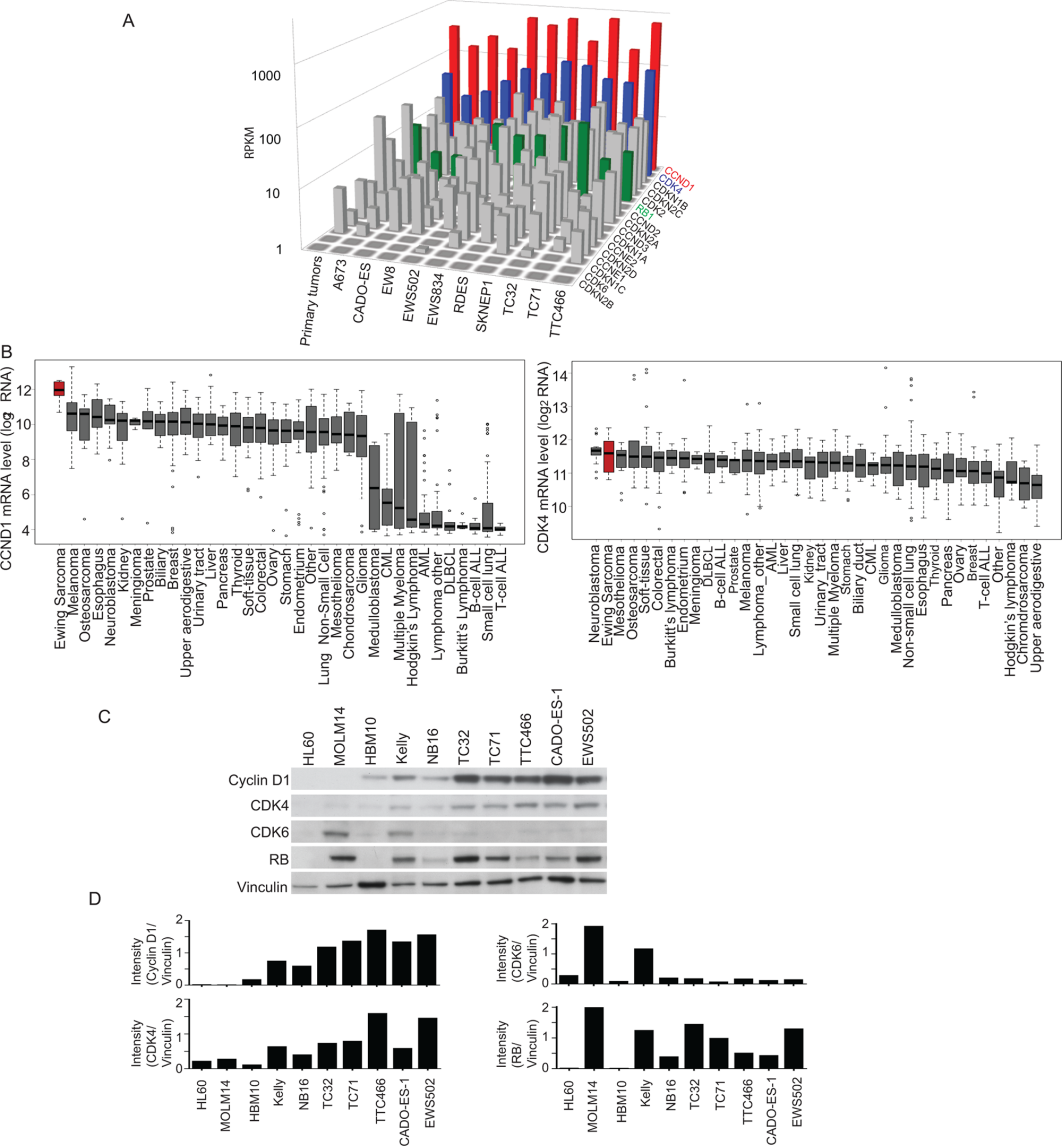
Cyclin D1 and CDK4 are highly expressed in Ewing sarcoma as compared to other tumor types **A.** RNA sequencing of Ewing sarcoma cell lines shows elevated levels of CCND1 (red) and CDK4 (blue) compared to other G1 cell cycle proteins. Rb is also highly expressed (green). **B.** CCND1 and CDK4 are highly expressed in Ewing sarcoma cell lines, as measured by mRNA expression, when compared to a panel of other non-Ewing sarcoma cancer cell lines. **C.** Western blot shows expression of cyclin D1 and CDK4, as well as expression of Rb in Ewing sarcoma cell lines versus leukemia cell lines (HL60 and MOLM14), mesenchymal stem cells (HBM10) and neuroblastoma cell lines (Kelly and NB16). **D.** Quantification of Western blots results in panel C using NIH ImageJ.

Based on the above findings, we focused our attention on cyclin D1/CDK4/6 and examined expression levels of the cell cycle pathway components in Ewing sarcoma cell lines and in primary human tumor samples. CDK4 also scored by ATARiS in the shRNA screen. We found that *CCND1* and *CDK4* were expressed at higher levels in Ewing sarcoma cells compared to other G1 checkpoint transcripts based on RNA sequencing data from primary patient Ewing sarcoma tumors and cell lines (Figure 4A). We analyzed the data for copy number changes in *CDK4* or chromosomal amplifications and found chromosomal arm level amplification resulting in three copies of *CDK4* in 25% of tumors, which coincided with a modest increase in CDK4 expression, but none of the cell lines had a copy gain in CDK4. There was no change in *CCND1* copy number in any of the human tumors. This data is consistent with prior reports of increased CDK4 expression and copy number amplification [34, 35].

To determine whether *CCND1* and *CDK4* are more highly expressed in Ewing sarcoma versus other tumors, we compared Ewing cells to other tumor types by using the data generated from the Cancer Cell Line Encyclopedia (CCLE) [36]. The CCLE resource offers genomic profiling for 1,036 cancer cell lines of more than twenty different tissue types, including ten Ewing sarcoma cell lines. Amongst this compendium of cell lines, the expression of *CCND1* is highest and the expression of *CDK4* is the second highest in the ten Ewing sarcoma cell lines (Figure 4B). In order to examine whether this comparative overexpression of transcripts was correlated with protein levels, we assessed by western blotting the expression of CDK4, cyclin D1 and Rb in Ewing sarcoma cells, mesenchymal stem cells, leukemia cell lines and neuroblastoma cells (Figure 4C–4D). Ewing sarcoma cells expressed high levels of protein when compared to these other cell types. Notably, cyclin D1, as well as its cell cycle regulatory partner, CDK4, were present in all Ewing sarcoma cell lines tested. In addition, Rb protein expression was detected in all Ewing sarcoma cell lines (Supplementary Figure S3A).

### Validation of shRNA targets

**Figure 5:**
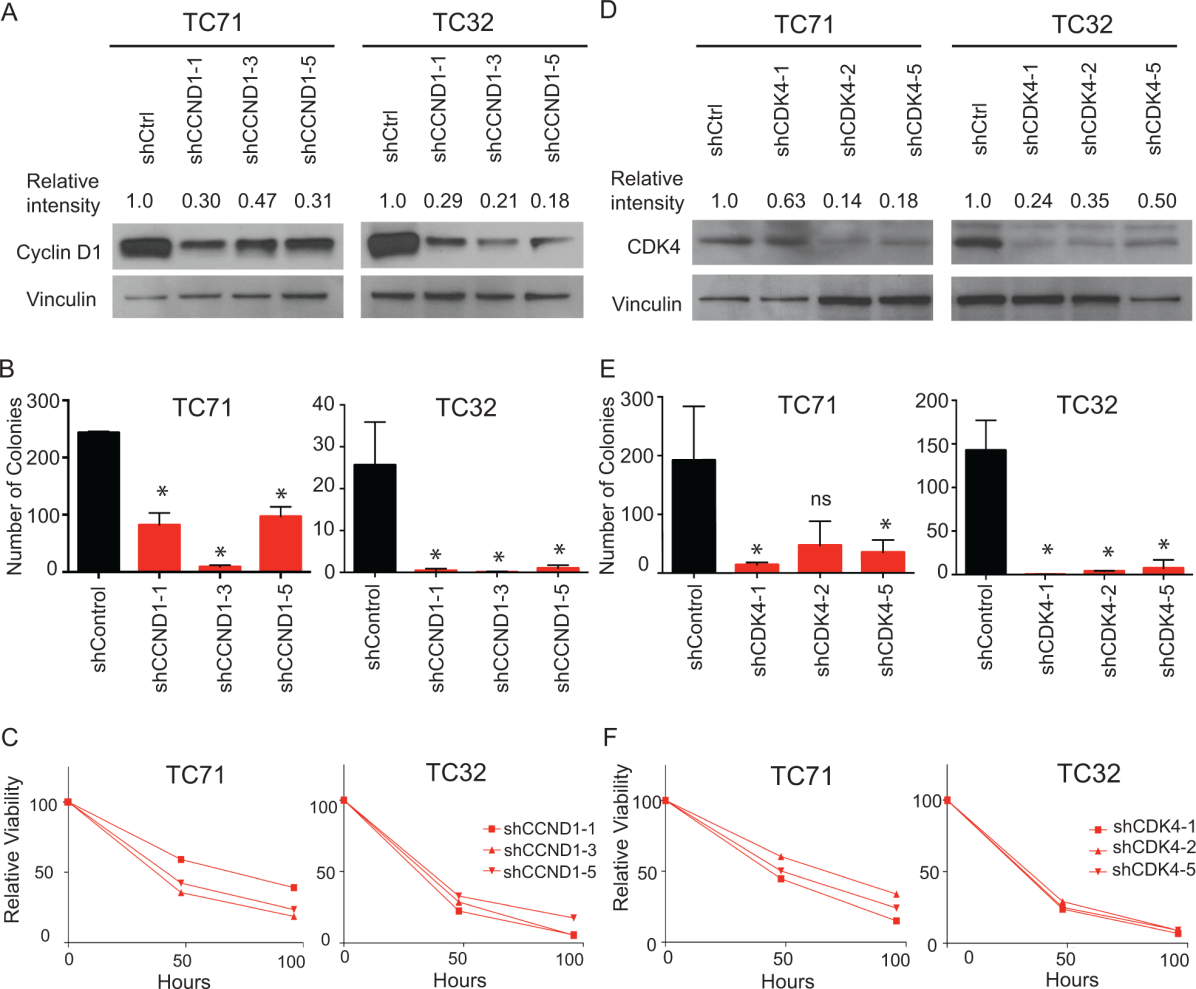
Targeting CDK4 and cyclin D1 with shRNA mediated knockdown results in impairment in both cell growth and colony formation **A.** Western blot showing decreased levels of cyclin D1 after transduction with shRNAs targeting cyclin D1 or a control. Relative intensity was quantified with NIH ImageJ. **B.** Decreased colony formation of cyclin D1 shRNA transduced cells versus control. Error bars represent +/− SEM of two independent experiments. *P-value < 0.05, ns: not significant. **C.** Relative viability over time of TC71 and TC32 cells transduced with indicated cyclin D1 targeting hairpins compared to cells transduced with control hairpin. Results were plotted as percentage of viability in cyclin D1 shRNA transduced cells compared to control shRNA transduced cells, based on the luminescent value at each timepoint. Shown is a representative of three independent experiments. **D.** Western blot showing decreased levels of CDK4 after transduction with shRNAs targeting CDK4 or control. Relative intensity was quantified with NIH ImageJ. **E.** Decreased colony formation of CDK4 shRNA transduced cells versus a control. Error bars represent +/− SEM of three independent experiments. *P-value < 0.05, ns: not significant. **F.** Relative viability over time of TC71 and TC32 cells transduced with indicated CDK4-targeting hairpins compared to cells transduced with a control hairpin. Results were plotted as the percentage of viability in cyclin D1 shRNA transduced cells compared to control shRNA transduced cells, based on the luminescent value at each timepoint. Shown is a representative of three independent experiments.

We next validated the results of our functional genomic screening by analyzing the cyclin D1/CDK4 dependency in Ewing sarcoma *in vitro*. We transduced TC32 and TC71 cell lines with shRNA targeting cyclin D1 or CDK4. Knockdown of cyclin D1 (Figure 5A) effectively depleted cyclin D1 levels in TC32 and TC71 cells as shown by western blotting. Ewing sarcoma cells were dependent on cyclin D1 (Figure 5B) for colony formation in methylcellulose, a measurement of anchorage independent growth. In addition, hairpins targeting cyclin D1 resulted in markedly decreased cell viability over time as compared to control shRNAs (Figure 5C). Similar results were obtained when using shRNA targeting CDK4 (Figure 5D–5F). These data suggest that there is a dependency of Ewing sarcoma cells on the cyclin D1/CDK4 pathway.

### Small molecule validation in Ewing sarcoma cells

**Figure 6:**
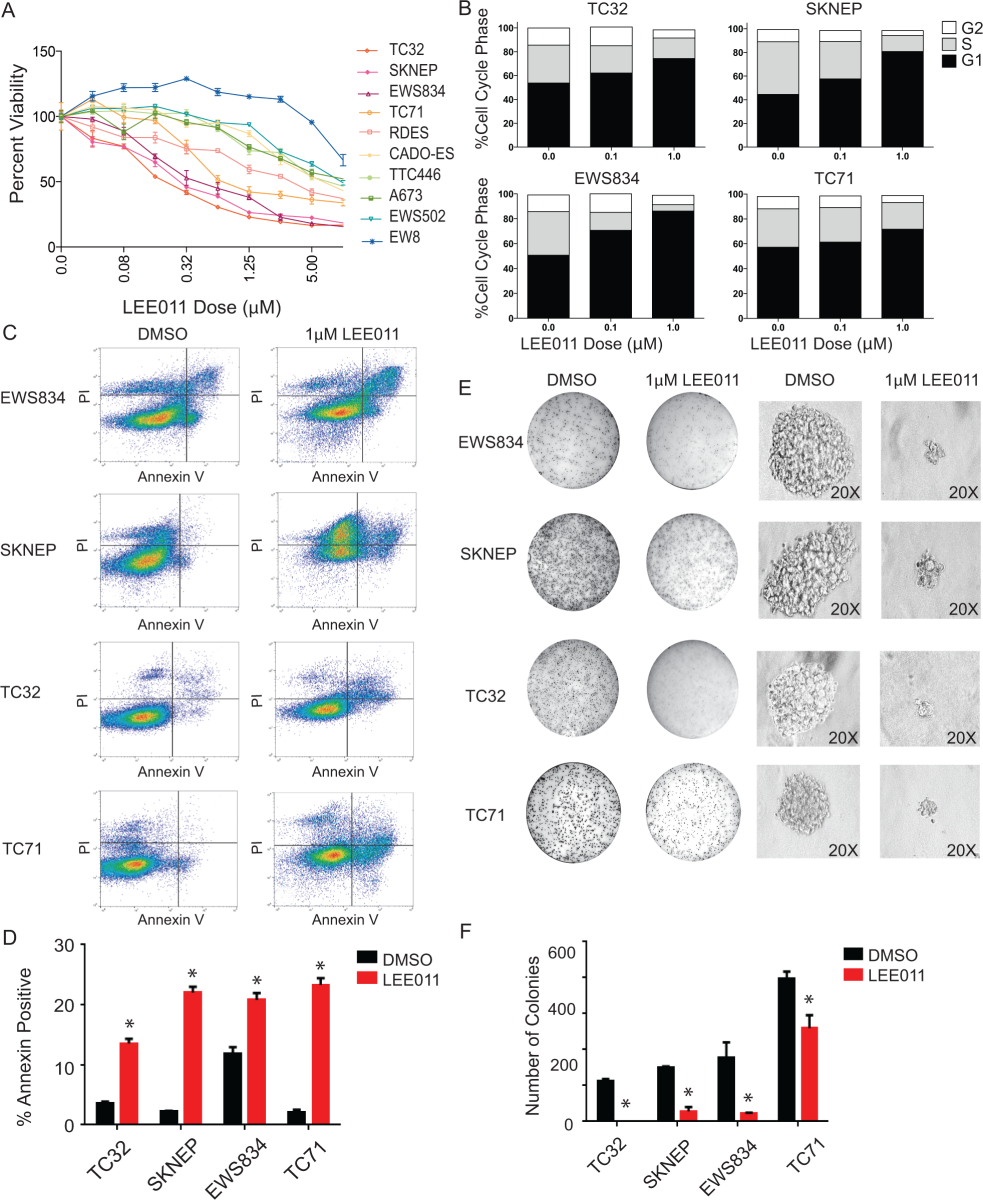
Pharmacologic inhibition of CDK4/6 in Ewing sarcoma results in impaired cell viability, G1 arrest and cell death in Ewing sarcoma cell lines **A.** Ewing sarcoma cells treated with LEE011 for five days show sensitivity in the low micromolar range in a panel of Ewing cell lines. Cell viability was assessed using the ATP-based CellTiter-Glo Luminescent Assay. **B.** Treatment with LEE011 in Ewing cell lines demonstrates G1 arrest. **C.** Flow cytometry plots for Annexin V and PI staining in the cell lines treated with 1 μM LEE011 or DMSO for 5 days. **D.** Percentage of Annexin V-positive cells in the cell lines treated with 1 μM LEE011 or DMSO for 5 days. Error bar represent +/− SD of two technical replicates. *P-value < 0.05, ns: not significant. Results are representative of three independent experiments. **E.** Colony morphology in methylcellulose-based media with 1 μM LEE011 versus control. **F.** Colony counts in methylcellulose-based media with 1 μM LEE011 versus control. Results are representative of three independent experiments, with error bars representing +/− SD of two technical replicates.*P-value < 0.05, ns: not significant.

To complement these shRNA studies, we tested the effects of a selective small-molecule inhibitor of CDK4/6, LEE011, in Ewing sarcoma cells. A panel of Ewing sarcoma cell lines was cultured with serially diluted concentrations of LEE011 and cell viability measured following five days of treatment. Target inhibition was confirmed by western blotting for phosphorylated RB at serine 780 (Supplementary Figure S3B) and the range of IC_50_s was 0.26–11.8 μM (Figure 6A and Supplementary Table S5). Sensitivity to LEE011 was correlated with sensitivity to PD0332991 (Supplementary Figure S4A) and was not based on intrinsic cell growth properties of the various Ewing lines tested (Supplementary Figure S4B). Because CDK4/6 inhibitors primarily induce a G1 cell cycle arrest in cancer cells with a functional Rb protein, we examined the effect of CDK4/6 inhibition on cell cycle in Ewing sarcoma cells. We observed a concentration-dependent G1 arrest in the majority of Ewing sarcoma cell lines tested (Figure 6B). We also noted decreased growth over time in many of the cell lines tested (Supplementary Figure S4C). In addition, cell death was observed as determined by trypan blue exclusion (Supplementary Figure S4D). In support of this observation, there was a marked increase in annexin V positivity by flow cytometry in some of the cell lines tested (Figure 6C–6D). In addition, when treated with 1 μM LEE011, Ewing sarcoma cell lines had decreased growth in methylcellulose, as demonstrated by smaller and fewer colonies formed in the presence of LEE011 compared to DMSO (Figure 6E–6F). These studies establish LEE011 as a potent inducer of cell cycle arrest and cell death in a subset of Ewing sarcoma cell lines.

### Ewing sarcoma xenograft responds to CDK4/6 inhibition

**Figure 7:**
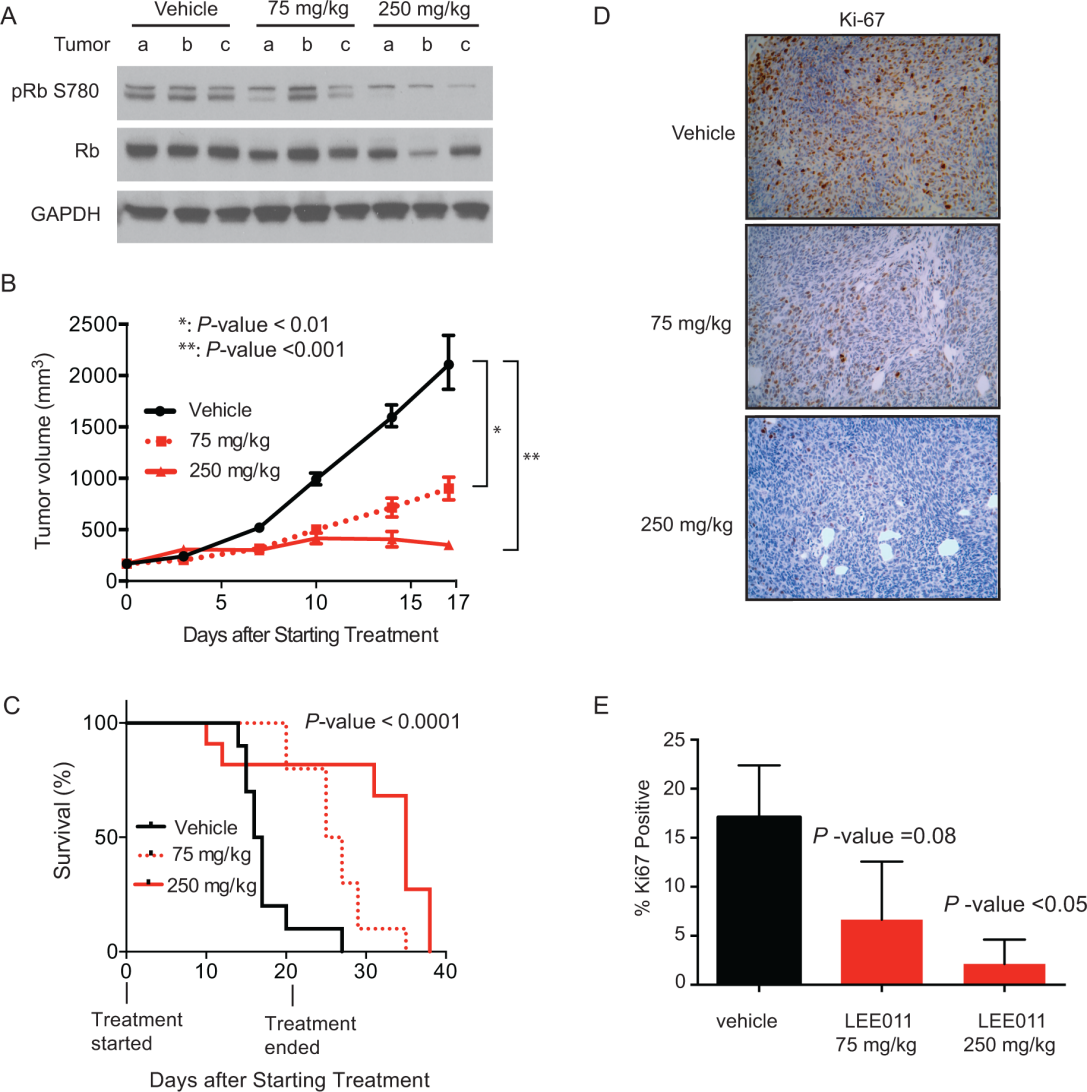
LEE011 impairs Ewing sarcoma tumor xenograft growth *in vivo* **A.** Western blot showing LEE011 effects on the downstream target of CDK4, Rb phosphoserine 780, total Rb or GAPDH in tumor cell lysates. **B.** Tumor volume over time in animals treated with vehicle, LEE011 at 75 mg/kg or 250 mg/kg. **C.** Kaplan-Meier survival curve of control, 75 mg/kg or 250 mg/kg daily dosing of LEE011. Log-rank (Mantel-Cox) test (*P*-value < 0.0001) and Logrank test for trend (*P*-value < 0.0001) were used to calculate *P*-value comparing the three survival curves. **D.** Immunohistochemical staining for proliferation marker Ki-67. **E.** Quantification of Ki-67 staining compared to vehicle.

Following the *in vitro* validation of cyclin D1 and CDK4 through shRNA-mediated knockdown and small-molecule inhibition, we next assessed the efficacy of LEE011 *in vivo*. NSG mice injected with subcutaneous TC32 Ewing sarcoma cells were treated with vehicle, 75 mg/kg or 250 mg/kg of LEE011 by daily oral gavage for 21 days after tumors were established. With daily dosing, especially with the higher dose, a few mice experienced a minor weight loss, which was alleviated with two drug-free holidays per week (Supplementary Figure S5A). Pharmacodynamic measurement of target inhibition performed at five days after initiation of treatment (Figure 7A) revealed a dose-dependent decrease in the cyclin D1/CDK4 complex dependent Rb phosphorylation site, phospho-serine 780, in resected tumors as evaluated by western immunoblotting. In addition, there was a statistically significant difference in tumor volume in LEE011 versus vehicle treatment (Figure 7B). Notably, at the highest dose of LEE011 treatment there was even a modest regression of tumor size. Most importantly, there was a significant difference in survival in animals treated with LEE011 as compared to vehicle (Figure 7C). Ki67 staining was significantly decreased in animals treated with the highest dose of LEE011, indicative of decreased cell proliferation (Figure 7D and 7E). In summary, these data demonstrate the *in vivo* efficacy of LEE011 in a Ewing sarcoma model.

## DISCUSSION

Ewing sarcoma remains a significant clinical challenge, particularly when patients present with metastases at diagnosis or recurrent disease. Patients with localized disease have an overall survival of 75–80% with intensive chemotherapy and aggressive local control [1, 2, 37]. Unfortunately, only a minority of patients with high-risk disease are long-term survivors. Although the promise of targeted, individualized therapies has begun to positively impact some patients with cancer, success has largely been in targeting mutated kinases. In the recent characterization of the genomic landscape of Ewing sarcoma, one of the most remarkable findings has been the marked absence of such recurrent kinase events. Furthermore, while numerous efforts are underway to target EWS/FLI1 directly, clinical success in these endeavors has yet to be achieved. New therapeutics aimed at the downstream effectors of this EWS/FLI1 oncogene are needed.

Recent developments in high-throughput screening technologies, including functional genomic screening, small-molecule screens and epigenetic screening, combined with cancer genomics, have led to the unbiased nomination of new therapeutic targets. Data mining of many of these published or publically available screening resources allows for increased confidence in the biological relevance of such targets. Our combination of super-enhancer, functional genomic, and small-molecule screening has converged on the discovery of a dependency on cyclin D1 and CDK4 in Ewing sarcoma. We show that Ewing sarcoma cells have an activated cyclinD1/CDK4 pathway and are sensitive to both chemical inhibition of CDK4 and shRNA suppression of CDK4 and cyclin D1. We demonstrate that inhibition of CDK4 leads to cytostasis in a panel of Ewing sarcoma cell lines and cell death in a subset of these. *In vivo*, a CDK4/6 inhibitor decreased tumor growth and prolonged survival in a Ewing sarcoma xenograft model. Our xenograft studies indicate an important *in vivo* response at both treatment doses of LEE011; the lower dose has been demonstrated to be clinically achievable and the higher dose was tested to enable comparison to previously published data in other models.

We recognize that our approach, albeit fruitful for this study, has several limitations. Combinatorial screening approaches may have led to exclusion of targets (false negatives) that may prove to be important in Ewing sarcoma biology. For example, in the current study we focused on protein coding genes in the near-whole-genome shRNA screening. Intriguingly, we noticed that several noncoding RNAs are associated with some top-ranking super-enhancers and are highly expressed. In addition, we narrowed our target list based on the publically available chemical genomic screening data, which is limited by the compounds included in the library. Many potential targets currently lack effective chemical inhibitors. For example, no GSK-3α selective inhibitors were represented in the library and pan-GSK3 inhibitors represented in the library might fail to yield a relative sensitivity in Ewing sarcoma. Therefore, our screening data may be re-mined for new targets as available data and analytical algorithms evolve, and as selective targeted therapies become available, providing valuable resources for the future study of Ewing sarcoma biology.

In our analysis, cyclin D1 is a hit in the combinatorial screening in Ewing sarcoma. Physiologic upregulation of cyclin D1 occurs in response to mitogens and leads to cell cycle progression through binding of cyclin D1 to cyclin dependent kinases, including CDK4 and CDK6. Upon activation, these kinases phosphorylate the G1 cell cycle gatekeeper, RB [38]. Once phosphorylated, Rb is inhibited and dissociates from the E2F family of G1 to S phase-promoting transcription factors, allowing for cell cycle progression. Deregulation of the cyclin D1/CDK4/6/Rb pathway leads to increased cell proliferation; this signaling axis is one of the most frequently mutated pathways in cancers [39]. There are several proposed mechanisms by which cyclin D1/CDK4 may be deregulated in Ewing sarcoma. First, cancer genome sequencing has demonstrated disruption of this pathway through *CDKN2A* deletion, a potential biomarker of sensitivity to CDK4/6 inhibitors [40], in approximately 13–30 percent of Ewing sarcoma tumors [10, 11, 41, 42]. Another mechanism reported to enhance cyclin D1/CDK4 pathway activation in Ewing sarcoma is via the EWS/FLI1 fusion protein binding the *CCND1* gene promoter resulting in increased expression of cyclin D1 protein [25]. In addition, this pathway is rendered more oncogenic through increased EWS/FLI1-dependent expression of the cyclin D1B isoform in Ewing sarcoma [43]. There are also reports of *CDK4* gene amplification in Ewing sarcoma [34]. In this work, we have demonstrated that an additional mechanism leading to increased expression of cyclin D1 and activation of the cyclin D1/CDK4 pathway is the presence of a cyclin D1 super-enhancer. Consistent with prior reports of super-enhancers in other tumors, these broad peaks are associated with increased expression of transcripts. In the case of Ewing sarcoma, EWS/FLI1 is an important component of the super-enhancer machinery.

Due to the frequent deregulation of the cyclin D1/CDK4 oncogenic pathway, there has been much interest in the development of specific CDK4/6 inhibitors. In cancer cells that have overactive CDK4/6-cyclin D1 activity and a functional Rb protein, CDK4/6 inhibitors block Rb phosphorylation, preventing its deactivation, and leading to G1 cell-cycle arrest or senescence [44]. Importantly, from a clinical perspective, mutations in *RB* are the strongest known predictor of resistance to CDK4/6 inhibitors, and Ewing sarcomas rarely contain *RB* mutations [10]. LEE011 and other orally available CDK4/6 inhibitors, such as PD0332991, are currently in clinical trials, and these CDK4/6 inhibitors have had promising pre-clinical activity in many different tumor types, including pediatric neuroblastoma [44] and rhabdomyosarcoma [45]. Notably, these CDK4/6 inhibitors have had clinical efficacy in estrogen receptor positive breast cancer [46] and liposarcoma [47].

Single agent chemotherapy is rarely curative. In Ewing sarcoma, combinations of doxorubicin, vincristine, ifosfamide, cyclophosphamide and etoposide are first line chemotherapeutic agents and require DNA replication for efficacy. Given the prior reports of antagonism of CDK4/6 inhibitors with conventional cytotoxics [48], which require cell cycling for efficacy, use of CDK4/6 inhibitors in combination with standard cytotoxic chemotherapy will likely require sequential delivery regimens. Alternatively, CDK4/6 inhibitors in Ewing might be used concurrently with other novel therapeutics that do not require S-phase cell cycling for efficacy. Indeed, there are emerging reports suggesting that synergy can be achieved with the combination of CDK4/6 inhibitors and PI3K inhibitors in breast cancer [49] or with insulin-like growth factor receptor 1 (IGF1R) inhibitors in pancreatic cancer [50]. Because Ewing sarcoma has been shown to have susceptibility to IGF1R inhibitors, with modest activity observed in clinical trials [51], this novel combination may prove interesting in Ewing sarcoma and will be the focus of future investigation. Taken together, our data reveal sensitivity of Ewing sarcoma cells to CDK4/6 inhibition and support the further study of selective CDK4/6 inhibitors in this disease.

## MATERIALS AND METHODS

### Cell lines and chemical compounds

Ewing cell lines were kindly provided by Todd Golub, except for EWS502 and EWS834, which were gifts from Jonathan Fletcher. Mesenchymal stem cells (HBM10) were a gift from Alejandro Sweet-Cordero. Kelly and NB16 cells were a gift from Rani George. All Ewing sarcoma cell lines were confirmed to have an *EWS/FLI1* or *EWS/ERG* rearrangement by RNA sequencing. Please refer to Supplementary Methods for details regarding cell culture conditions. Ewing sarcoma cell lines were treated with the CDK4/6 inhibitor LEE011, kindly provided by Novartis Oncology, and PD0332991 (Selleck Chemicals).

### ChIP-seq and ChIP-qPCR

Chromatin IP-sequencing was performed as described previously [17]; please see Supplementary Methods for details. The following antibodies were used for ChIP on the two human Ewing sarcoma cell lines TC32 and TC71: EWS/FLI1: anti-FLI1 antibody SC-356X (Santa Cruz Biotechnology), H3K27Ac: ab4729 (Abcam), H3K4me3: ab8580 (Abcam) and H3K27me3: 07-449 (EMD Millipore).

### Genomics of drug sensitivity in cancer data analysis, shRNA screen and analysis, and cancer cell line encyclopedia (CCLE) data analysis

For details about these data analyses, please refer to the Supplementary Methods.

### Lentivirus production and transduction

Lentivirus was produced by transfecting HEK-293T cells with the appropriate pLKO.1 lentiviral vector and packaging plasmids pCMV8.9 and pCMV-VSVG according to the FuGENE 6 (Roche) protocol or with Xtremegene 9 (Thermo-scientific) for Mir30 plasmids. Plasmids were obtained from The RNAi Consortium (Broad Institute). For lentiviral transduction, Ewing sarcoma cells were incubated with 2 mL of virus and 8 μg/mL of polybrene (Sigma-Aldrich). Cells were selected in puromycin (Sigma-Aldrich) 48 hours post-transduction. shRNA target sequences are listed in Supplementary Table S6.

### Determination of cell viability and colony formation

Cell viability was assessed using the CellTiter-Glo Luminescent Cell Viability Assay (Promega). Colony formation assays were performed after Ewing sarcoma cell lines were transduced with shRNAs targeting CDK4 and CCND1 or treatment with LEE011 or a DMSO control. Please refer to Supplementary Methods for details.

### Cell cycle analysis and cell death assays

The effect of LEE011 treatment on Ewing sarcoma cell cycling was measured at 72 and 120 hours post-treatment. Cells were harvested, washed and fixed in ethanol and then re-suspended in 40 μg/mL propidium iodide (Sigma-Aldrich) and 100 μg/mL of RNase A (Qiagen). Samples were analyzed on a FACSCanto II analyzer. Ewing sarcoma cells lines were assessed for cell death after small-molecule treatment at 120 hours post-treatment. Cell death was assessed using flow cytometric analysis of annexin V and propidium iodide staining according to the manufacturer's instructions (eBioscience). Data analysis was completed using Flowjo 7.6 software (Treestar).

### Protein extraction and immunoblotting

Whole-cell lysates were extracted in 1x Cell Lysis Buffer (Cell Signaling) supplemented with EDTA-free protease inhibitors and PhosSTOP phosphatase inhibitors (Roche). Western immunoblotting was performed using standard techniques. Primary antibodies utilized included GAPDH (Santa Cruz, sc-137179), Vinculin (Abcam, ab18058), CDK4 (Thermo Fisher Scientific, MS-469-P0), cyclin D1 (EMD Millipore, 04-1151), CAV1 (BD Transduction Lab, 610493), cyclin D3 (Santa Cruz, sc-182), pRb S780 (Cell Signaling, 9307), pRb S795 (Cell Signaling, 9301), and total Rb (Cell Signaling, 9309).

### Establishment of tumor xenografts and *in vivo* LEE011 treatment

Tumor xenografts were established in 45 NOD/SCID/gamma female mice using 5 × 10^6^ TC32 cells resuspended in 30% matrigel and injected into the flank. Animals bearing engrafted tumors of 100–250 mm^3^ were randomized to oral gavage treatment with 75 mg/kg (*n* = 13) or 250 mg/kg (*n* = 13) LEE011 in 0.5% methylcellulose or vehicle (*n* = 13) for a total of 21 days. Three mice per cohort were sacrificed to evaluate the pharmacodynamics of the compound following the fifth dose. Tumor volumes were measured with calipers and animals were sacrificed when tumor volumes exceeded 2,000 mm^3^. Tumor xenografts were flash frozen or formalin fixed for further immunohistochemical staining with Ki67 using traditional staining methods. These Ki67 stained slides were then imaged in three different fields per tumor using a Nikon microscope, IHC images were quantified, and then the average of the three images per slide and per treatment group were quantified using the Immunoratio software program [52]. All animal studies were conducted under the auspices of protocols approved by the Dana-Farber Cancer Institute Animal Care and Use Committee.

## SUPPLEMENTARY METHODS








