# Supplementary material for: Functional, chemical genomic, and super-enhancer screening identify sensitivity to cyclin D1/CDK4 pathway inhibition in Ewing sarcoma
Source: Oncotarget. 2015 Aug 18;6(30):30178–93. doi: 10.18632/oncotarget.4903 (PMC4745789; doi:10.18632/oncotarget.4903)
Supplement: Supplementary file 1 [file oncotarget-06-30178-s001.pdf]

SUPPLEMENTARY METHODS

Cell culture conditions

All cell lines were cultured at 37°C in a humidified atmosphere containing 5% CO_2. A673, EW8, SKNEP, EWS834, RDES, Kelly and NB16 cells were grown in Dulbecco's Modified Eagle's Media (Life Technologies) supplemented with 10% fetal bovine serum (FBS) (Sigma-Aldrich). A673 cells were cultured in media supplemented with 1 mmol/L of sodium pyruvate (Life Technologies). CADO-ES-1, TTC466 and EWS502 cell lines were cultured in RPMI 1640 media (Life Technologies) supplemented with 15% FBS. MOLM-14 and HL60 were grown in RPMI supplemented with 10% FBS. TC71 and TC32 lines were cultured in 10% RPMI1640. All cells were grown in the presence of 10 units/mL of penicillin, 10 µg/mL streptomycin and 30 µg/mL of L-Glutamine, PSQ (Thermo Fisher Scientific).

Determination of cell viability

Cell viability was assessed using the CellTiter-Glo Luminescent Cell Viability Assay (Promega). Luminescent readings were obtained using the FLUOstar Omega microplate reader (BMG Labtech). Cells were treated at a range of concentrations and IC_{50} values were calculated from ATP luminescence measurements after five days of treatment using log-transformed, normalized data in GraphPad Prism 5.0 (GraphPad Software, Inc.). Cell viability was also determined following shRNA-tranduction of Ewing sarcoma cells with pLKO.1 lentiviral shRNAs targeting CDK4 and CCND1. ATP content was measured at days 3, 5, 6 and 8 after transduction.

Colony formation assay

Three days after transduction with shRNAs targeting CDK4 and CCND1, 5 x10^3 cells were distributed into 2 mL of methylcellulose matrix ClonaCell-TCS Medium, (Stemcell Technologies), subsequently plated into gridded 6-cm plates (Thermo Fisher Scientific) and incubated for 10 to 15 days. Colonies were counted from 100 squares using a Nikon inverted microscope. All samples were plated in duplicate and adjusted for input based on CellTiter-Glo Luminescent Cell Viability Assay (Promega) as a surrogate for cell number input. Colony formation assay was also performed with LEE011 or DMSO dissolved into matrix. Colonies were stained with MTT dye (Roche Diagnostics) diluted 1:1 in PBS at approximately ten days following plating and counted using ImageQuant software.

ChIP-seq and ChIP-qPCR

Briefly, crosslinking was performed in cell culture medium containing 1% formaldehyde with gentle rotation for 5 minutes in room temperature. Fixation was stopped by the addition of glycine at 125 mM final concentration. Fixed cells were trypsinized, washed twice in ice-cold PBS, and then resuspended in SDS lysis buffer (1% SDS, 10 mM EDTA, 50 mM Tris-HCl, pH 8.1) supplemented with Complete mini-protease inhibitor cocktail (Roche). Chromatin was sheared to approximately 200 base pair fragments by Covaris ultra-sonication. After shearing, the samples were spun down at maximal speed for 10 mins and then the supernatant was incubated with antibodies overnight at 4°C and subsequently incubated with Protein A/G beads for two hours at 4°C. Precipitates were washed sequentially with ice cold low salt wash (0.1% SDS, 1% Triton-X-100, 2 mM EDTA, 20 mM Tris-HCl, pH 8.1, 150 mM NaCl), high salt wash (0.1% SDS, 1% Triton-X-100, 2 mM EDTA, 20 mM Tris-HCl, pH 8.1, 500 mM NaCl), LiCl wash (0.25 M LiCl, 1% IGEPAL CA-630, 1% deoxycholic acid, 1 mM EDTA, 10 mM Tris-HCl, pH 8.1) and TE wash (1 mM EDTA, 10 mM Tris-HCl, pH 8.1) and eluted in elution buffer (1% SDS, 0.1 M NaHCO3). Eluted DNA fragments were analyzed by qPCR or barcoded with NEBNext DNA library preparation kit NEB and subjected to sequencing on the Illumina HiSeq 2000 platform.

Specific primers were designed based on the location of FLI1-ChIP-seq peaks and H3K27Ac-ChIP-seq peaks in the enhancer region of individual genes. Primers for EWS/FLI1 ChIP-qPCR are CAV1 forward: AGATCTT TTGCTGACCCATGCT; reverse: AAATGACA GCTC TATACCCCAACAT, CCND1 forward: CCATCAA GCAGTCAAC AAATGC; reverse: TGTAGGAAAAACA GCTCTCTG GAA, SMARCA4 forward: TGGGAC TAATGAGCTGCT AAACAT; reverse: TTTGGCTTAGT TACTTCTTGGTT GAG; primers for H3K27Ac-ChIP-qPCR are CAV1 forward: TTCAGGCTT CCGGATCCA; reverse: TACCATGCTTTTGTTGTGATACTGTTAA, CCN D1 forward: CCCATCTGTGTAAAGGAAACAAACT; reverse: TCAACAGAC CCCCATCTTGTC, SMARCA4 forward: TGGGACTAATGAGCTGCTAAACAT; reverse: TTTGGCTTAGTTACTTCTTGGTTGAG.

Gene set enrichment analysis (GSEA)

The GSEA v2.0.14 software [1, 2] was utilized to identify pathways or groups of EWS/FLI1 functionally related genes that have a significant overlap with genes marked by the H3K27Ac histone. To apply GSEA, the genome-wide list of genes was ranked by the H3K27Ac ChIP-seq occupancy signal in the 50 kb proximity of the TSS. The goal of GSEA was to identify the gene sets that are distributed at the top or at the bottom of the ranked list of genes. GSEA assigns to each gene set an enrichment score calculated as a Kolmogorov-Smirnoff running sum statistic by walking down the ranked list of genes, increasing the sum when encountering genes in the pathway or in the gene set of interest, and decreasing

the sum otherwise. The significance of the enrichment score (ES) was estimated based on a permutation *P*-value adjusted for multiple hypotheses or false disovery rate (FDR). Gene sets with a nominal *P*-value ≤ 0.05 and an FDR ≤ 0.25 were considered significant. The lists of actively transcribed genes in the 50 kb proximity of H3K27Ac super-enhancers in the TC32 (321 genes) and in the TC71 (239 genes) cell lines were tested for significant overlap with the comprehensive collection c2 of 4,722 curated canonical pathways and gene sets, available in the MSigDB v4.0 database http://www.broadinstitute.org/gsea/msigdb/index.jsp. The test was run by applying the "Investigate GeneSets" module available in MSigDB. The significance of the overlap was assessed based on a hypergeometric test, with the 0.05 cut-off for the FDR.

ChIP-seq data analysis

All ChIP-seq data sets were aligned using Bowtie v2.2.3 [3] to build version NCBI37/HG19 of the human genome. In order to identify enhancer regions, we profiled H3K27Ac in the TC32 and TC71 cell lines by following the procedures described in Hnisz *et al.* [4]. Active enhancers were identified based on the algorithm described in Loven *et al.* [5]. ChIP-Seq enriched regions were defined using MACS v1.4.2 [6] with the cutoff 1e-09 for the *P*-value. According to Loven *et al.* [5] active enhancers were defined as significantly enriched regions which are outside promoters (i.e., not fully contained within ±2.5 kb region flanking the promoter). The active enhancers within 12.5 kb of one another were stitched together, and the stitched regions spanning more than two promoters were excluded. The total ChIP occupancy signal at enhancers expressed in units of reads per million mapped reads per bp (rpm/bp) was computed by using the area under curve formula based on the H3K27Ac normalized signal with background input signal subtracted. Following Loven *et al.*, the stitched active enhancers were ranked based on increasing total ChIP occupancy normalized signal and an inflection point in the signal distribution was geometrically detected. Super-enhancers were defined as those stitched active enhancers with total ChIP occupancy signal above the inflection cut-off. Actively transcribed genes were assessed based on the existence of significantly enriched regions of H3K4me3 within +/− 5 kb of the transcription starting site (TSS) [5] combined with RNASeq expression status RPKM scores >1 [7]. In order to verify that these genes were not bivalently marked or repressed, we assayed the repressive histone mark H3K27me3 and required that no significantly enriched region for H3K27me3 overlapped within +/− 5 kb of the TSS gene promoter region. The metagene representations of global genome-wide average ChIP-seq signal occupancy of H3K27Ac and EWS/FLI1 at super-enhancers vs. typical enhancers were created based on the method described in Loven *et al*. [5]. The ChIP-seq signal was mapped to the +/− 5 kb regions flanking the center of the active super-enhancer or typical enhancer. Each of the flanking regions and the active enhancer region were split into 50 bp bins. The active enhancer regions were aligned. The normalized ChIP-seq signal occupancy (with background input subtracted) was then computed as the "Area Under Curve" density signal (in rpm/bp units) in the flanked active enhancer region. Finally the meta-representations were obtained based on the average normalized ChIP-seq signal across the super-enhancers versus typical enhancers.

The presence of GGAA repeats in the proximity of super-enhancers and EWS/FLI1 peaks were annotated using the Homer v2.4.7 software package [8]. A whole genome search for GGAA microsatellites was performed using the software tool SciRoKo v3.4 [9]. The overlap of the collection of GGAA microsatellites with EWS/FLI1 peaks was analyzed with the BEDTools v2.17.0 software suite [10].

shRNA screen and analysis

This screen was performed in the Achilles v2.4.3 dataset consisting of 216 cell lines from 22 cancer types, with an shRNA library of 54,020 barcoded shRNAs in lentiviral vectors targeting 11,194 genes. These data are available through the Achilles Project at http://www.broadinstitute.org/achilles/. Four *EWS/FLI1*-rearranged Ewing sarcoma cell lines (A673, EW8, EWS502, TC71) and one *EWS/ERG* rearranged cell line (CADO-ES-1) were included in the screen. Ewing sarcoma dependencies were identified by employing the previously described Analytic Technique for Assessment of RNAi by Similarity (ATARiS) [11]. The Ewing dependencies were identified for the Ewing sarcoma cell lines compared with all other 211 tumor cell lines, and repeatedly for the Ewing sarcoma cell lines compared to 87 other cancer cell lines most dissimilar to Ewing sarcoma in terms of their gene expression profile. Functional non-similarity with Ewing sarcoma was estimated based on a consensus of published EWS/FLI1 gene signatures [12–15] and principal components analysis (PCA) applied on matched gene expression data. The genome-wide list of genes was then ranked based on their depletion scores in Ewing sarcoma. Significance was assessed based on the cut-off 0.05 applied to *P*-values adjusted for multiple occurrences.

Genomics of drug sensitivity in cancer data analysis

The Genomics of Drug Sensitivity project in Cancer (http://cancerrxgene.org) provides compound, cancer gene and cell line data to identify molecular features of cancers that predict response to anti-cancer drugs.

A Mann-Whitney test was performed in data release 5 (June 2014) to compare the drug sensitivity of Ewing sarcoma cell lines versus other cell lines.

Cancer cell line encyclopedia (CCLE) data analysis

The Cancer Cell Line Encyclopedia Project (http://www.broadinstitute.org/ccle/) [16] provides genomic data for 1036 cell lines of 24 tissue types, including 10 Ewing Sarcoma cell lines: A673, SK-N-MC, RDES, SK-ES-1, TC71, MHH-ES-1, CADO-ES-1, EWS502, TC32, EW8. For CCND1 and CDK4, the relative expression across different tumor types was visualized as boxplots ranked by the median expression within each tumor type.

REFERENCES

1. Subramanian A, Tamayo P, Mootha VK, Mukherjee S, Ebert BL, Gillette MA, Paulovich A, Pomeroy SL, Golub TR, Lander ES, et al. Gene set enrichment analysis: a knowledge-based approach for interpreting genome-wide expression profiles. Proc Natl Acad Sci U S A. 2005; 102:15545–15550.
2. Mootha VK, Lindgren CM, Eriksson KF, Subramanian A, Sihag S, Lehar J, Puigserver P, Carlsson E, Ridderstrale M, Laurila E, et al. PGC-1alpha-responsive genes involved in oxidative phosphorylation are coordinately downregulated in human diabetes. Nat Genet. 2003; 34:267–273.
3. Langmead B, Salzberg SL. Fast gapped-read alignment with Bowtie 2. Nat Methods. 2012; 9:357–359.
4. Hnisz D, Abraham BJ, Lee TI, Lau A, Saint-Andre V, Sigova AA, Hoke HA, Young RA. Super-enhancers in the control of cell identity and disease. Cell. 2013; 155:934–947.
5. Loven J, Hoke HA, Lin CY, Lau A, Orlando DA, Vakoc CR, Bradner JE, Lee TI, Young RA. Selective inhibition of tumor oncogenes by disruption of super-enhancers. Cell. 2013; 153:320–334.
6. Zhang Y, Liu T, Meyer CA, Eeckhoute J, Johnson DS, Bernstein BE, Nusbaum C, Myers RM, Brown M, Li W, et al. Model-based analysis of ChIP-Seq (MACS). Genome Biol. 2008; 9:R137.
7. Crompton B, Stewart C, Taylor-Weiner A, Alexe G, Kurek K, Calicchio M, Kiezun A, et al. The Genomic Landscape of Pediatric Ewing Sarcoma. Cancer Discovery. Published OnlineFirst September 3, 2014.
8. Heinz S, Benner C, Spann N, Bertolino E, Lin YC, Laslo P, Cheng JX, Murre C, Singh H, Glass CK. Simple combinations of lineage-determining transcription factors prime cis-regulatory elements required for macrophage and B cell identities. Mol Cell. 2010; 38:576–589.
9. Kofler R, Schlotterer C, Lelley T. SciRoKo: a new tool for whole genome microsatellite search and investigation. Bioinformatics. 2007; 23:1683–1685.
10. Quinlan AR, Hall IM. BEDTools: a flexible suite of utilities for comparing genomic features. Bioinformatics. 2010; 26:841–842.
11. Shao DD, Tsherniak A, Gopal S, Weir BA, Tamayo P, Stransky N, Schumacher SE, Zack TI, Beroukhim R, Garraway LA, et al. ATARiS: computational quantification of gene suppression phenotypes from multisample RNAi screens. Genome Res. 2013; 23:665–678.
12. Riggi N, Suva ML, Suva D, Cironi L, Provero P, Tercier S, Joseph JM, Stehle JC, Baumer K, Kindler V, et al. EWS-FLI-1 expression triggers a Ewing's sarcoma initiation program in primary human mesenchymal stem cells. Cancer Res. 2008; 68:2176–2185.
13. von Levetzow C, Jiang X., Gwye, Y. , von Levetzow G, Hung L, Cooper A, Hsu JH, Lawlor ER. Modeling initiation of Ewing sarcoma in human neural crest cells. PLoS One. 2011; 6:e19305.
14. Staege MS, Hutter C, Neumann I, Foja S, Hattenhorst UE, Hansen G, Afar D, Burdach SE. DNA microarrays reveal relationship of Ewing family tumors to both endothelial and fetal neural crest-derived cells and define novel targets. Cancer Res. 2004; 64:8213–8221.
15. Kinsey M, Smith R, Lessnick SL. NR0B1 is required for the oncogenic phenotype mediated by EWS/FLI in Ewing's sarcoma. Mol Cancer Res. 2006; 4:851–859.
16. Barretina J, Caponigro G, Stransky N, Venkatesan K, Margolin AA, Kim S, Wilson CJ, Lehar J, Kryukov GV, Sonkin D, et al. The Cancer Cell Line Encyclopedia enables predictive modelling of anticancer drug sensitivity. Nature. 2012; 483:603–607.

SUPPLEMENTARY FIGURES AND TABLES

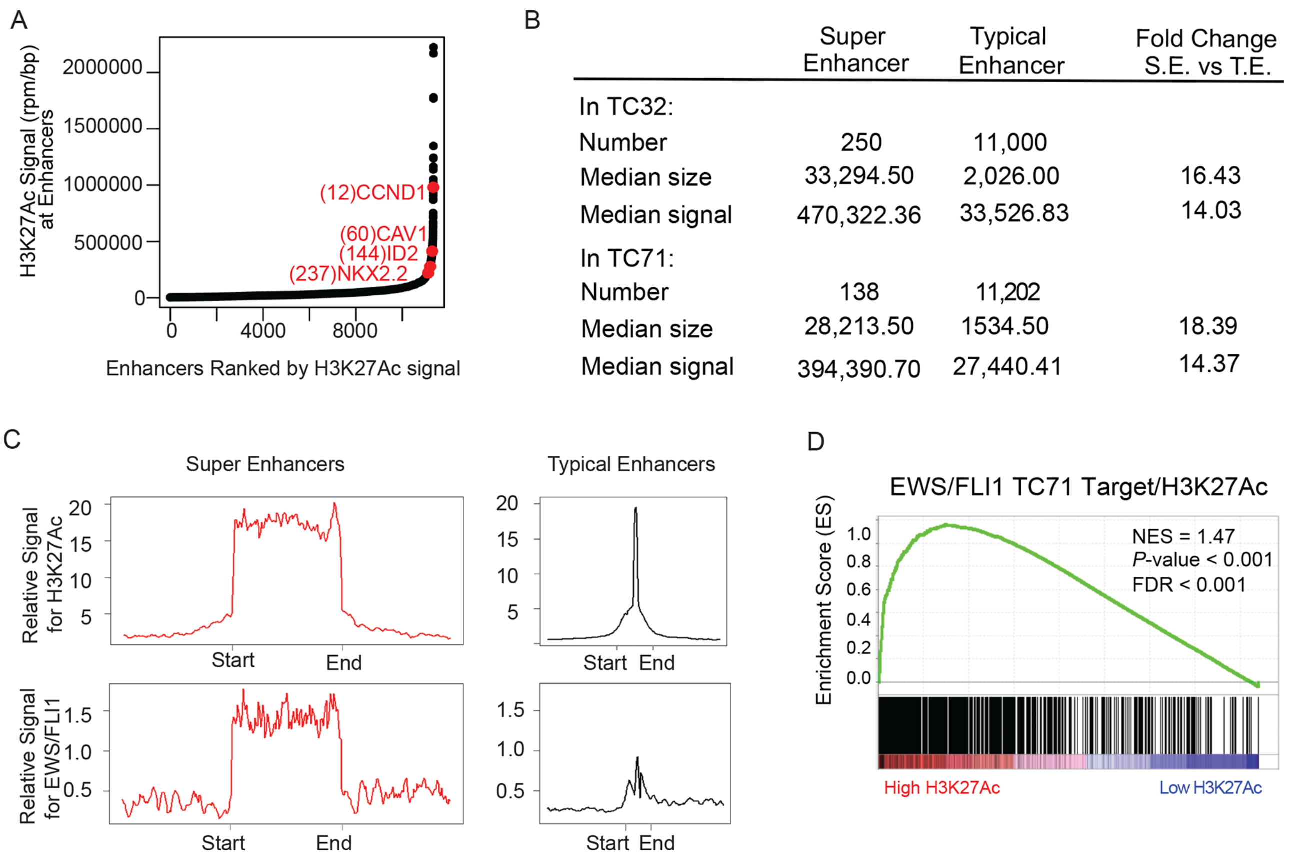

Supplementary Figure S1: Superenhancer profiling identifies CCND1 and other important Ewing sarcoma oncogenic targets in TC71 cells. A. Total H3K27Ac ChIP-seq signal in units of reads per million in enhancer regions for all enhancers in TC71. Enhancers are ranked by increasing H3K27Ac ChIP-seq signal. **B.** Chart showing comparison of typical and super-enhancer size. **C.** Metagene representation of global H3K27Ac or EWS/FLI1 occupancy at typical enhancers and super-enhancers. Metagenes are centered on the enhancer region, and the length of the enhancer reflects the difference in median length. Additional 5 kb surrounding each enhancer is also shown. The y axis shows the relative ChIP-seq signal. **D.** GSEA demonstrates enrichment of H3K27Ac binding in EWS/FLI1 target genes.

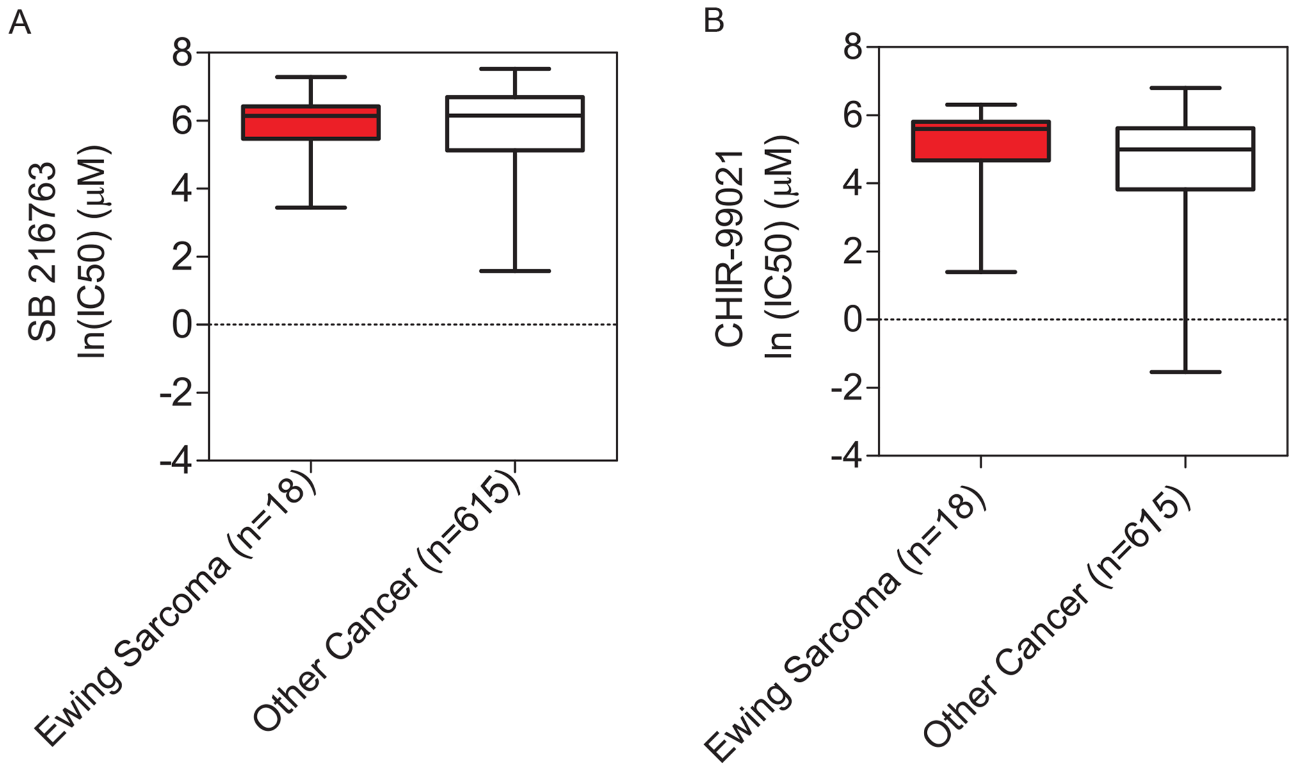

Supplementary Figure S2: Boxplots of the ln(IC50) scores in the Ewing Sarcoma cell lines vs the Other Cancer cell lines for the compounds SB216763 A. and CHIR-99021 B. based on the IC50 score data for each of these compounds across the 633 cell lines profiled in the Genomics of Drug Sensitivity Project in Cancer (http://cancerrxgene.org), release 5 (June 2014). *P*-Value > 0.05 in both cases.

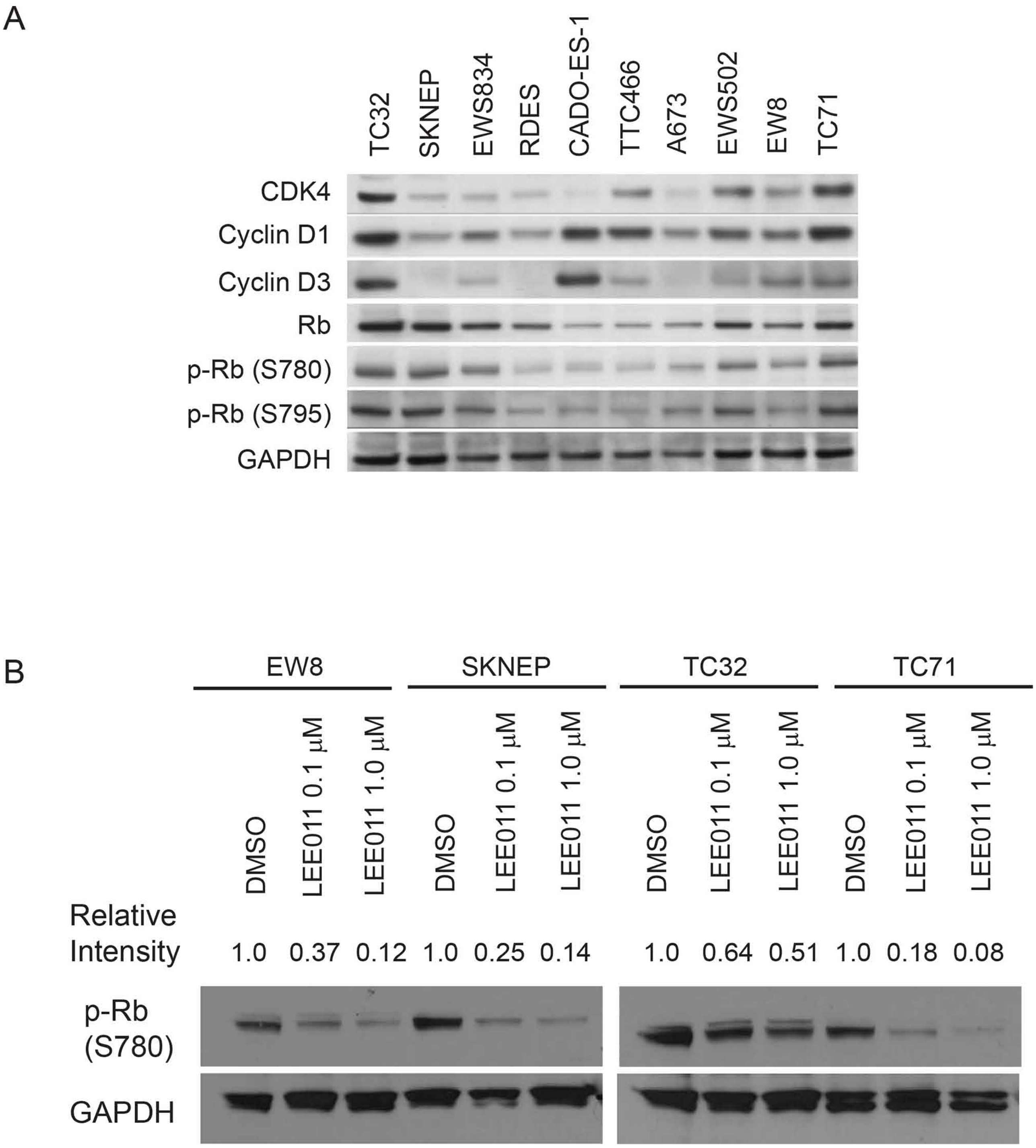

Supplementary Figure S3: G1 Cell cycle protein expression in Ewing sarcoma cells and LEE011 target validation. A. Western blots showing various G1 checkpoint cell cycle protein expression levels in Ewing sarcoma cell lines. **B.** LEE011 target validation showing decreased p-Rb S780 in LEE011 treated Ewing sarcoma cells versus DMSO treated cells. Relative intensity was quantified with NIH ImageJ.

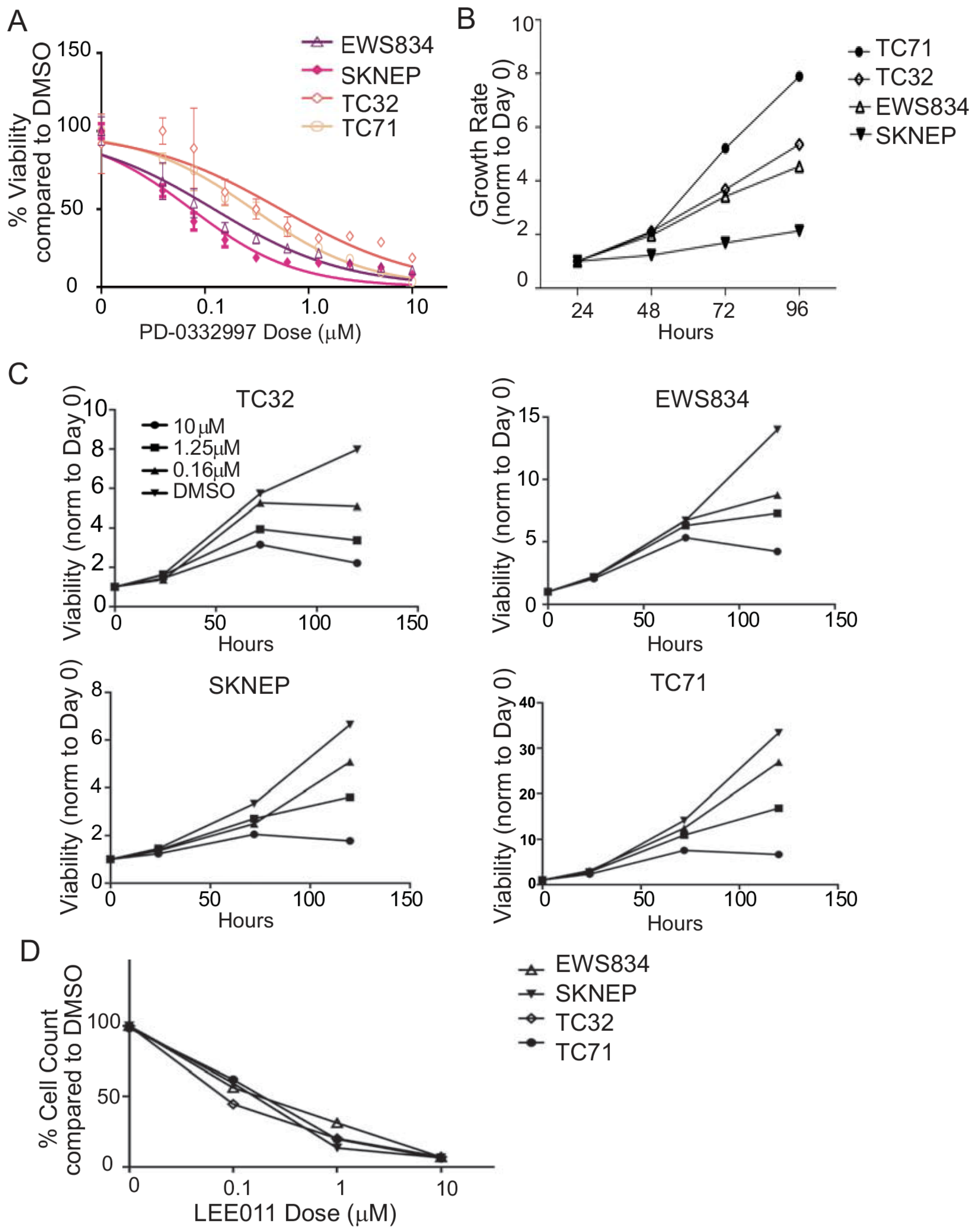

Supplementary Figure S4: Effects of PD-0332997 and LEE011 on Ewing sarcoma cell growth. A. Percent viability of Ewing sarcoma cells after 5 days of PD-0332997 treatment. ATP is quantified as a surrogate for cell number. Data are shown normalized to DMSO. Results are representative of two independent experiments. **B.** Growth of Ewing sarcoma cell lines over time in the absence of drug. ATP is quantified as a surrogate for cell number. Data are shown normalized to Day 0. Results are representative of three independent experiments. **C.** Ewing sarcoma cell lines treated with the indicated concentrations of LEE011 and ATP is measured as a surrogate for cell number. Data are shown normalized to Day 0. Results are representative of three independent experiments. **D.** Effects of LEE011 on cell number after 5 days of treatment as measured by Trypan Blue exclusion. Data are shown as a percentage of the DMSO control. Results are representative of three independent experiments.

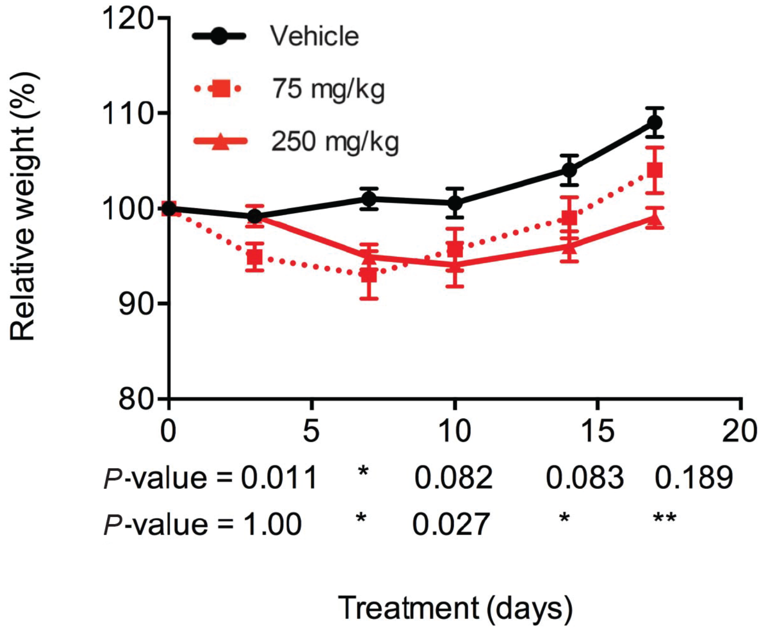

Supplementary Figure S5: Relative weight over time in LEE011-treated mice versus vehicle-treated at various doses. *P*-values are listed for each time point.

Supplementary Table S1: List of actively transcribed target genes associated with the top 500 H3K27Ac enhancers in the TC32 Ewing sarcoma cell line. The enhancers are ranked based on their AUC signal. Enhancers with no actively transcribed target gene are marked by "na" in the Target Gene Symbol field. Super-enhancers rank from 1 to 250 and they target 321 actively transcribed genes.

Supplementary Table S2: List of actively transcribed target genes associated with the top 500 H3K27Ac enhancers in the TC71 Ewing sarcoma cell line. The enhancers are ranked based on their AUC signal. Enhancers with no actively transcribed target gene are marked by "na" in the Target Gene Symbol field. Super-enhancers rank from 1 to 138 and they target 239 actively transcribed genes.

Supplementary Table S3: Top 20 gene set enrichment analysis results for the 321 actively transcribed target genes associated with the 250 H3K27Ac super-enhancers for the TC32 cell line

#	Gene Set Name	# Gene Set (*K*)	# Overlap (*k*)	*k*/*K*	*P*-value	FDR *q*-value
1	NIKOLSKY_BREAST_CANCER_1Q21_AMPLICON	38	15	0.39	1.94E-23	6.92E-20
2	PATIL_LIVER_CANCER	747	39	0.05	2.93E-23	6.92E-20
3	RIGGI_EWING_SARCOMA_PROGENITOR_UP	430	30	0.07	1.26E-21	1.98E-18
4	BENPORATH_EED_TARGETS	1062	39	0.04	5.95E-18	7.02E-15
5	LOCKWOOD_AMPLIFIED_IN_LUNG_CANCER	214	20	0.09	2.89E-17	2.52E-14
6	WAKABAYASHI_ADIPOGENESIS_PPARG_RXRA_BOUND_8D	882	35	0.04	3.20E-17	2.52E-14
7	KINSEY_TARGETS_OF_EWSR1_FLII_FUSION_UP	1278	41	0.03	8.67E-17	5.85E-14
8	BLALOCK_ALZHEIMERS_DISEASE_UP	1691	46	0.03	4.79E-16	2.83E-13
9	BENPORATH_SUZ12_TARGETS	1038	36	0.03	7.16E-16	3.76E-13
10	ZHANG_TARGETS_OF_EWSR1_FLI1_FUSION	88	14	0.16	9.44E-16	4.46E-13
11	BENPORATH_ES_WITH_H3K27ME3	1118	37	0.03	1.19E-15	5.11E-13
12	MARTENS_TRETINOIN_RESPONSE_DN	841	32	0.04	2.54E-15	1.00E-12
13	MEISSNER_BRAIN_HCP_WITH_H3K4ME3_AND_H3K27ME3	1069	35	0.03	1.02E-14	3.71E-12
14	WANG_TUMOR_INVASIVENESS_UP	374	22	0.06	1.24E-14	4.18E-12
15	MIYAGAWA_TARGETS_OF_EWSR1_ETS_FUSIONS_UP	259	19	0.07	1.58E-14	4.98E-12
16	CASORELLI_ACUTE_PROMYELOCYTIC_LEUKEMIA_DN	663	27	0.04	8.59E-14	2.54E-11
17	BENPORATH_PRC2_TARGETS	652	26	0.04	4.07E-13	1.13E-10
18	GRESHOCK_CANCER_COPY_NUMBER_UP	323	19	0.06	8.25E-13	2.16E-10
19	ENK_UV_RESPONSE_KERATINOCYTE_UP	530	23	0.04	1.74E-12	4.33E-10
20	LEI_MYB_TARGETS	318	18	0.06	6.33E-12	1.49E-09

The enrichment analysis was performed on the c2 collection of 4,722 curated Canonical Pathways and Experimental Gene Sets available from the MSigDB v4.0 database.

Supplementary Table S4: List of 425 significant Ewing Sarcoma gene dependencies identified based on the ATARiS method in the Achilles v2.4.3 data. The dependencies were estimated for five Ewing cell lines (A673, EW8, EWS502, TC71, CADO-ES-1) vs all other 211 tumor cell lines. Significance was assessed based on the adjusted *P*-value cut-off 0.05.

Supplementary Table S5: List of LEE011 IC50s in a panel of ten Ewing cell lines

#	Cell line	IC50 (µM)
1	TC32	0.26
2	SKNEP	0.36
3	EWS834	0.56
4	TC71	1.58
5	RDES	3.19
6	CADO-ES	6.38
7	TTC466	6.91
8	A673	8.63
9	EWS502	8.67
10	EW8	11.78

Cell lines are ranked in increasing order of IC50 (µM) scores.

Supplementary Table S6: List of PLKO and TRIPZ shRNA clones used to knock down CCND1, CDK4 and FLI1

Name	Vector	Clone ID
shCtrl	PLKO	na
shCCND1-1	PLKO	TRCN0000040038
shCCND1-2	PLKO	TRCN0000040042
shCCND1-5	PLKO	TRCN0000010317
shCDK4-1	PLKO	TRCN0000000362
shCDK4-3	PLKO	TRCN0000000364
shCDK4-5	PLKO	TRCN0000010520
shCtrl	TRIPZ	RHS4743
shFLI-6	TRIPZ	V2THS227524
shFLI-7	TRIPZ	V3THS414176
