# Supplementary material for: Functional, chemical genomic, and super-enhancer screening identify sensitivity to cyclin D1/CDK4 pathway inhibition in Ewing sarcoma
Source: Oncotarget. 2015 Aug 18;6(30):30178–93. doi: 10.18632/oncotarget.4903 (PMC4745789; doi:10.18632/oncotarget.4903)
Supplement: Supplementary file 2 [file oncotarget-06-30178-s002.doc]

| TC32 #  **Supplementary Table 1.** List of actively transcribed target genes associated with the top 500 H3K27Ac enhancers in the **TC32** Ewing sarcoma cell line. The enhancers are ranked based on their AUC signal. Enhancers with no actively transcribed target gene are marked by "na" in the Target Gene Symbol field. Super-enhancers rank from 1 to 250 and they target 321 actively transcribed genes. | Enhancer chr | Enhancer start | Enhancer end | Enhancer AUC | Enhancer  Rank | Enhancer Is SE? | Target Gene Symbol | Target Gene TC32 RPKM |
| --- | --- | --- | --- | --- | --- | --- | --- | --- |
| 1 | chr6 | 26087132 | 26287126 | 2599922.00 | 1 | 1 | HIST1H2BD | 2.04 |
| 2 | chr22 | 46430464 | 46489462 | 2535734.04 | 2 | 1 | na | na |
| 3 | chr7 | 130558852 | 130656695 | 2311051.66 | 3 | 1 | na | na |
| 4 | chr1 | 185613264 | 185706974 | 2024136.00 | 4 | 1 | HMCN1 | 5.79 |
| 5 | chr7 | 116101587 | 116180485 | 1970083.06 | 5 | 1 | CAV1 | 9.63 |
| 6 | chr7 | 116101587 | 116180485 | 1970083.06 | 5 | 1 | CAV2 | 5.51 |
| 7 | chr8 | 27039640 | 27099864 | 1753722.88 | 6 | 1 | na | na |
| 8 | chr19 | 13100968 | 13183275 | 1706224.11 | 7 | 1 | NFIX | 6.83 |
| 9 | chr17 | 47049476 | 47113351 | 1590487.50 | 8 | 1 | IGF2BP1 | 5.83 |
| 10 | chr12 | 54348250 | 54432297 | 1570838.43 | 9 | 1 | HOXC6 | 4.43 |
| 11 | chr12 | 54348250 | 54432297 | 1570838.43 | 9 | 1 | HOXC9 | 4.09 |
| 12 | chr12 | 54348250 | 54432297 | 1570838.43 | 9 | 1 | HOXC11 | 3.91 |
| 13 | chr12 | 54348250 | 54432297 | 1570838.43 | 9 | 1 | HOXC10 | 3.86 |
| 14 | chr12 | 54348250 | 54432297 | 1570838.43 | 9 | 1 | HOXC12 | 3.75 |
| 15 | chr12 | 54348250 | 54432297 | 1570838.43 | 9 | 1 | HOXC4 | 3.50 |
| 16 | chr12 | 54348250 | 54432297 | 1570838.43 | 9 | 1 | HOTAIR | 2.90 |
| 17 | chr12 | 54348250 | 54432297 | 1570838.43 | 9 | 1 | HOXC8 | 2.16 |
| 18 | chr12 | 54348250 | 54432297 | 1570838.43 | 9 | 1 | HOXC5 | 1.61 |
| 19 | chr1 | 201468991 | 201535915 | 1355880.24 | 10 | 1 | CSRP1 | 7.57 |
| 20 | chr17 | 46787552 | 46849776 | 1281814.40 | 11 | 1 | na | na |
| 21 | chr11 | 69429579 | 69471543 | 1228705.92 | 12 | 1 | CCND1 | 9.54 |
| 22 | chr11 | 69429579 | 69471543 | 1228705.92 | 12 | 1 | ORAOV1 | 2.69 |
| 23 | chr6 | 27760839 | 27864220 | 1227132.47 | 13 | 1 | na | na |
| 24 | chr1 | 230293025 | 230385938 | 1167916.41 | 14 | 1 | na | na |
| 25 | chr17 | 79358663 | 79398404 | 1153681.23 | 15 | 1 | na | na |
| 26 | chr6 | 110642616 | 110691559 | 1059615.95 | 16 | 1 | METTL24 | 2.88 |
| 27 | chr16 | 23846983 | 23921777 | 1057587.16 | 17 | 1 | PRKCB | 6.57 |
| 28 | chr8 | 6101750 | 6125139 | 1049698.32 | 18 | 1 | na | na |
| 29 | chr2 | 30331536 | 30388030 | 1042314.30 | 19 | 1 | YPEL5 | 8.68 |
| 30 | chr14 | 97671516 | 97705635 | 967273.65 | 20 | 1 | na | na |
| 31 | chr1 | 145053687 | 145101248 | 964537.08 | 21 | 1 | SEC22B | 4.58 |
| 32 | chr1 | 145053687 | 145101248 | 964537.08 | 21 | 1 | PDE4DIP | 3.77 |
| 33 | chr1 | 38454886 | 38539808 | 952824.84 | 22 | 1 | POU3F1 | 5.59 |
| 34 | chr1 | 38454886 | 38539808 | 952824.84 | 22 | 1 | SF3A3 | 4.56 |
| 35 | chr1 | 38454886 | 38539808 | 952824.84 | 22 | 1 | FHL3 | 4.08 |
| 36 | chr1 | 38454886 | 38539808 | 952824.84 | 22 | 1 | UTP11L | 3.42 |
| 37 | chr18 | 10453405 | 10483973 | 925904.72 | 23 | 1 | APCDD1 | 8.98 |
| 38 | chr1 | 164599817 | 164697547 | 905957.10 | 24 | 1 | na | na |
| 39 | chr6 | 26019453 | 26072596 | 900773.85 | 25 | 1 | HIST1H1C | 2.47 |
| 40 | chr6 | 26019453 | 26072596 | 900773.85 | 25 | 1 | HFE | 1.76 |
| 41 | chr1 | 241679895 | 241727486 | 895186.71 | 26 | 1 | FH | 8.16 |
| 42 | chr1 | 241679895 | 241727486 | 895186.71 | 26 | 1 | KMO | 5.62 |
| 43 | chr1 | 65352732 | 65400925 | 884823.48 | 27 | 1 | na | na |
| 44 | chr1 | 236801724 | 236870448 | 876918.24 | 28 | 1 | ACTN2 | 3.06 |
| 45 | chr14 | 99696519 | 99741711 | 859551.84 | 29 | 1 | BCL11B | 4.73 |
| 46 | chr4 | 40190441 | 40215679 | 858849.14 | 30 | 1 | RHOH | 4.82 |
| 47 | chr14 | 102974923 | 103027639 | 856635.00 | 31 | 1 | ANKRD9 | 3.30 |
| 48 | chr1 | 155161591 | 155248159 | 848366.40 | 32 | 1 | SCAMP3 | 5.94 |
| 49 | chr1 | 155161591 | 155248159 | 848366.40 | 32 | 1 | FAM189B | 5.21 |
| 50 | chr1 | 155161591 | 155248159 | 848366.40 | 32 | 1 | CLK2 | 5.18 |
| 51 | chr1 | 155161591 | 155248159 | 848366.40 | 32 | 1 | GBA | 4.93 |
| 52 | chr1 | 155161591 | 155248159 | 848366.40 | 32 | 1 | THBS3 | 4.32 |
| 53 | chr1 | 155161591 | 155248159 | 848366.40 | 32 | 1 | MTX1 | 4.14 |
| 54 | chr1 | 155161591 | 155248159 | 848366.40 | 32 | 1 | GBAP1 | 3.38 |
| 55 | chr1 | 155161591 | 155248159 | 848366.40 | 32 | 1 | HCN3 | 2.83 |
| 56 | chr1 | 155161591 | 155248159 | 848366.40 | 32 | 1 | MUC1 | 2.03 |
| 57 | chr8 | 12773178 | 12813512 | 842980.60 | 33 | 1 | na | na |
| 58 | chr2 | 85964555 | 86022509 | 837435.30 | 34 | 1 | ATOH8 | 5.82 |
| 59 | chr5 | 133199048 | 133238908 | 830283.80 | 35 | 1 | na | na |
| 60 | chr13 | 112720219 | 112759276 | 828008.40 | 36 | 1 | SOX1 | 6.21 |
| 61 | chr1 | 214146367 | 214176127 | 814233.60 | 37 | 1 | PROX1 | 5.56 |
| 62 | chr7 | 115832476 | 115882494 | 812792.50 | 38 | 1 | TES | 5.81 |
| 63 | chr1 | 146514023 | 146557732 | 803371.42 | 39 | 1 | na | na |
| 64 | chr1 | 157938003 | 157993230 | 801896.04 | 40 | 1 | KIRREL | 5.20 |
| 65 | chr1 | 156071509 | 156101126 | 789589.22 | 41 | 1 | LMNA | 7.09 |
| 66 | chr1 | 156071509 | 156101126 | 789589.22 | 41 | 1 | MEX3A | 4.58 |
| 67 | chr12 | 114822567 | 114852265 | 786106.06 | 42 | 1 | TBX5 | 6.54 |
| 68 | chr12 | 114822567 | 114852265 | 786106.06 | 42 | 1 | TBX5-AS1 | 5.02 |
| 69 | chr1 | 198833979 | 198907890 | 783456.60 | 43 | 1 | na | na |
| 70 | chr11 | 65238458 | 65277023 | 756645.30 | 44 | 1 | SCYL1 | 4.69 |
| 71 | chr11 | 65238458 | 65277023 | 756645.30 | 44 | 1 | MALAT1 | 4.41 |
| 72 | chr9 | 4652718 | 4689684 | 751518.78 | 45 | 1 | CDC37L1 | 4.82 |
| 73 | chr9 | 4652718 | 4689684 | 751518.78 | 45 | 1 | SPATA6L | 2.27 |
| 74 | chr21 | 46330151 | 46361249 | 748839.84 | 46 | 1 | C21orf67 | 5.99 |
| 75 | chr21 | 46330151 | 46361249 | 748839.84 | 46 | 1 | FAM207A | 4.05 |
| 76 | chr21 | 46330151 | 46361249 | 748839.84 | 46 | 1 | ITGB2 | 2.46 |
| 77 | chr20 | 4443339 | 4491831 | 742412.52 | 47 | 1 | na | na |
| 78 | chr8 | 13316346 | 13374033 | 741277.95 | 48 | 1 | DLC1 | 5.02 |
| 79 | chr11 | 83426373 | 83461697 | 740391.04 | 49 | 1 | na | na |
| 80 | chr7 | 5456881 | 5470260 | 735711.21 | 50 | 1 | TNRC18 | 4.65 |
| 81 | chr12 | 6999072 | 7081180 | 729119.04 | 51 | 1 | C12orf57 | 6.85 |
| 82 | chr12 | 6999072 | 7081180 | 729119.04 | 51 | 1 | PHB2 | 6.78 |
| 83 | chr12 | 6999072 | 7081180 | 729119.04 | 51 | 1 | ATN1 | 6.34 |
| 84 | chr12 | 6999072 | 7081180 | 729119.04 | 51 | 1 | ENO2 | 4.63 |
| 85 | chr12 | 6999072 | 7081180 | 729119.04 | 51 | 1 | EMG1 | 3.68 |
| 86 | chr12 | 6999072 | 7081180 | 729119.04 | 51 | 1 | SCARNA12 | 2.04 |
| 87 | chr12 | 6999072 | 7081180 | 729119.04 | 51 | 1 | PTPN6 | 1.20 |
| 88 | chr12 | 6999072 | 7081180 | 729119.04 | 51 | 1 | LRRC23 | 1.03 |
| 89 | chr13 | 64640468 | 64698259 | 717186.31 | 52 | 1 | na | na |
| 90 | chr10 | 21803093 | 21824890 | 706222.80 | 53 | 1 | MLLT10 | 3.42 |
| 91 | chr10 | 21803093 | 21824890 | 706222.80 | 53 | 1 | SKIDA1 | 2.16 |
| 92 | chr13 | 31583262 | 31621470 | 705319.68 | 54 | 1 | na | na |
| 93 | chr11 | 16621368 | 16642024 | 703336.80 | 55 | 1 | na | na |
| 94 | chr11 | 63823790 | 63896346 | 701616.52 | 56 | 1 | na | na |
| 95 | chr9 | 126071237 | 126113711 | 692750.94 | 57 | 1 | na | na |
| 96 | chr18 | 10054168 | 10073176 | 671932.80 | 58 | 1 | na | na |
| 97 | chr12 | 72258743 | 72320760 | 666682.75 | 59 | 1 | na | na |
| 98 | chr1 | 183773334 | 183821158 | 666666.56 | 60 | 1 | na | na |
| 99 | chr1 | 161122630 | 161173567 | 662181.00 | 61 | 1 | NDUFS2 | 5.91 |
| 100 | chr1 | 161122630 | 161173567 | 662181.00 | 61 | 1 | UFC1 | 5.89 |
| 101 | chr1 | 161122630 | 161173567 | 662181.00 | 61 | 1 | B4GALT3 | 4.74 |
| 102 | chr1 | 161122630 | 161173567 | 662181.00 | 61 | 1 | DEDD | 4.41 |
| 103 | chr1 | 161122630 | 161173567 | 662181.00 | 61 | 1 | USP21 | 4.37 |
| 104 | chr1 | 161122630 | 161173567 | 662181.00 | 61 | 1 | ADAMTS4 | 4.32 |
| 105 | chr1 | 161122630 | 161173567 | 662181.00 | 61 | 1 | PPOX | 3.44 |
| 106 | chr1 | 161122630 | 161173567 | 662181.00 | 61 | 1 | TOMM40L | 2.18 |
| 107 | chr1 | 161122630 | 161173567 | 662181.00 | 61 | 1 | APOA2 | 2.08 |
| 108 | chr19 | 42745789 | 42791545 | 658886.40 | 62 | 1 | PAFAH1B3 | 6.63 |
| 109 | chr19 | 42745789 | 42791545 | 658886.40 | 62 | 1 | ERF | 6.12 |
| 110 | chr19 | 42745789 | 42791545 | 658886.40 | 62 | 1 | CIC | 4.82 |
| 111 | chr19 | 42745789 | 42791545 | 658886.40 | 62 | 1 | GSK3A | 4.53 |
| 112 | chr19 | 42745789 | 42791545 | 658886.40 | 62 | 1 | DEDD2 | 3.43 |
| 113 | chr19 | 42745789 | 42791545 | 658886.40 | 62 | 1 | ZNF526 | 2.59 |
| 114 | chr19 | 42745789 | 42791545 | 658886.40 | 62 | 1 | PRR19 | 1.82 |
| 115 | chr15 | 64263059 | 64320141 | 646739.06 | 63 | 1 | DAPK2 | 3.08 |
| 116 | chr3 | 34300990 | 34337630 | 645963.20 | 64 | 1 | na | na |
| 117 | chr1 | 156697271 | 156738664 | 644489.01 | 65 | 1 | HDGF | 7.14 |
| 118 | chr1 | 156697271 | 156738664 | 644489.01 | 65 | 1 | MRPL24 | 6.02 |
| 119 | chr1 | 156697271 | 156738664 | 644489.01 | 65 | 1 | PRCC | 5.79 |
| 120 | chr1 | 156697271 | 156738664 | 644489.01 | 65 | 1 | ISG20L2 | 3.42 |
| 121 | chr1 | 156697271 | 156738664 | 644489.01 | 65 | 1 | RRNAD1 | 3.23 |
| 122 | chr1 | 153505033 | 153542157 | 641502.72 | 66 | 1 | S100A6 | 7.09 |
| 123 | chr1 | 164721059 | 164760919 | 631781.00 | 67 | 1 | na | na |
| 124 | chr17 | 6914965 | 6957724 | 627274.53 | 68 | 1 | SLC16A11 | 5.78 |
| 125 | chr17 | 6914965 | 6957724 | 627274.53 | 68 | 1 | RNASEK | 3.85 |
| 126 | chr17 | 6914965 | 6957724 | 627274.53 | 68 | 1 | SLC16A13 | 2.67 |
| 127 | chr17 | 6914965 | 6957724 | 627274.53 | 68 | 1 | C17orf49 | 1.14 |
| 128 | chr5 | 43038336 | 43069414 | 623735.46 | 69 | 1 | ANXA2R | 2.45 |
| 129 | chr8 | 75018563 | 75044420 | 623670.84 | 70 | 1 | na | na |
| 130 | chr3 | 35676378 | 35722900 | 622929.58 | 71 | 1 | ARPP21 | 3.60 |
| 131 | chr16 | 2008747 | 2090503 | 621345.60 | 72 | 1 | RPS2 | 9.66 |
| 132 | chr16 | 2008747 | 2090503 | 621345.60 | 72 | 1 | NDUFB10 | 6.59 |
| 133 | chr16 | 2008747 | 2090503 | 621345.60 | 72 | 1 | NPW | 5.73 |
| 134 | chr16 | 2008747 | 2090503 | 621345.60 | 72 | 1 | ZNF598 | 5.09 |
| 135 | chr16 | 2008747 | 2090503 | 621345.60 | 72 | 1 | TBL3 | 4.84 |
| 136 | chr16 | 2008747 | 2090503 | 621345.60 | 72 | 1 | NTHL1 | 4.64 |
| 137 | chr16 | 2008747 | 2090503 | 621345.60 | 72 | 1 | TSC2 | 4.33 |
| 138 | chr16 | 2008747 | 2090503 | 621345.60 | 72 | 1 | SLC9A3R2 | 4.17 |
| 139 | chr16 | 2008747 | 2090503 | 621345.60 | 72 | 1 | SYNGR3 | 3.04 |
| 140 | chr16 | 2008747 | 2090503 | 621345.60 | 72 | 1 | SNHG9 | 2.85 |
| 141 | chr16 | 2008747 | 2090503 | 621345.60 | 72 | 1 | SNORA10 | 2.29 |
| 142 | chr16 | 2008747 | 2090503 | 621345.60 | 72 | 1 | GFER | 1.88 |
| 143 | chr16 | 2008747 | 2090503 | 621345.60 | 72 | 1 | SNORA64 | 1.63 |
| 144 | chr9 | 14302339 | 14324939 | 619918.00 | 73 | 1 | NFIB | 4.46 |
| 145 | chr1 | 230401866 | 230447978 | 619745.28 | 74 | 1 | na | na |
| 146 | chr11 | 16364566 | 16397393 | 618460.68 | 75 | 1 | na | na |
| 147 | chr12 | 120694476 | 120731991 | 607743.00 | 76 | 1 | PXN | 6.36 |
| 148 | chr8 | 37583489 | 37606316 | 604915.50 | 77 | 1 | ZNF703 | 6.50 |
| 149 | chr8 | 37583489 | 37606316 | 604915.50 | 77 | 1 | PROSC | 4.13 |
| 150 | chr8 | 37583489 | 37606316 | 604915.50 | 77 | 1 | ERLIN2 | 3.79 |
| 151 | chr17 | 46617997 | 46660074 | 601280.33 | 78 | 1 | HOXB2 | 3.05 |
| 152 | chr17 | 46617997 | 46660074 | 601280.33 | 78 | 1 | HOXB5 | 2.95 |
| 153 | chr17 | 46617997 | 46660074 | 601280.33 | 78 | 1 | HOXB6 | 2.69 |
| 154 | chr17 | 46617997 | 46660074 | 601280.33 | 78 | 1 | HOXB3 | 2.29 |
| 155 | chr17 | 46617997 | 46660074 | 601280.33 | 78 | 1 | HOXB4 | 1.95 |
| 156 | chr17 | 46617997 | 46660074 | 601280.33 | 78 | 1 | HOXB-AS3 | 1.72 |
| 157 | chr1 | 209790365 | 209823552 | 601016.57 | 79 | 1 | CAMK1G | 5.19 |
| 158 | chr9 | 132241663 | 132263547 | 585178.16 | 80 | 1 | na | na |
| 159 | chr6 | 99271406 | 99293953 | 584869.18 | 81 | 1 | POU3F2 | 5.59 |
| 160 | chr10 | 18422847 | 18454253 | 584465.66 | 82 | 1 | CACNB2 | 3.17 |
| 161 | chr1 | 218622758 | 218673720 | 583005.28 | 83 | 1 | na | na |
| 162 | chr17 | 43999844 | 44033224 | 578809.20 | 84 | 1 | MAPT | 5.31 |
| 163 | chr19 | 1237083 | 1276886 | 577541.53 | 85 | 1 | CIRBP | 6.38 |
| 164 | chr19 | 1237083 | 1276886 | 577541.53 | 85 | 1 | ATP5D | 5.66 |
| 165 | chr19 | 1237083 | 1276886 | 577541.53 | 85 | 1 | MIDN | 4.29 |
| 166 | chr19 | 1237083 | 1276886 | 577541.53 | 85 | 1 | C19orf24 | 2.53 |
| 167 | chr19 | 1237083 | 1276886 | 577541.53 | 85 | 1 | C19orf26 | 1.22 |
| 168 | chr11 | 795581 | 863109 | 571286.88 | 86 | 1 | RPLP2 | 9.59 |
| 169 | chr11 | 795581 | 863109 | 571286.88 | 86 | 1 | POLR2L | 9.19 |
| 170 | chr11 | 795581 | 863109 | 571286.88 | 86 | 1 | CD151 | 6.25 |
| 171 | chr11 | 795581 | 863109 | 571286.88 | 86 | 1 | TSPAN4 | 5.98 |
| 172 | chr11 | 795581 | 863109 | 571286.88 | 86 | 1 | PNPLA2 | 5.75 |
| 173 | chr11 | 795581 | 863109 | 571286.88 | 86 | 1 | PIDD | 3.58 |
| 174 | chr11 | 795581 | 863109 | 571286.88 | 86 | 1 | SLC25A22 | 3.36 |
| 175 | chr11 | 795581 | 863109 | 571286.88 | 86 | 1 | EFCAB4A | 2.89 |
| 176 | chr11 | 795581 | 863109 | 571286.88 | 86 | 1 | SNORA52 | 1.46 |
| 177 | chr2 | 183867143 | 183904340 | 567998.19 | 87 | 1 | NCKAP1 | 6.50 |
| 178 | chr13 | 46675826 | 46701785 | 565646.61 | 88 | 1 | na | na |
| 179 | chr9 | 132455773 | 132500869 | 563700.00 | 89 | 1 | na | na |
| 180 | chr3 | 33955690 | 33999501 | 563409.46 | 90 | 1 | na | na |
| 181 | chr1 | 18955952 | 18974237 | 559155.30 | 91 | 1 | PAX7 | 5.85 |
| 182 | chr17 | 67263945 | 67324362 | 557648.91 | 92 | 1 | ABCA5 | 5.37 |
| 183 | chr1 | 153917025 | 153964411 | 555363.92 | 93 | 1 | RPS27 | 9.02 |
| 184 | chr1 | 153917025 | 153964411 | 555363.92 | 93 | 1 | JTB | 6.78 |
| 185 | chr1 | 153917025 | 153964411 | 555363.92 | 93 | 1 | SLC39A1 | 6.04 |
| 186 | chr1 | 153917025 | 153964411 | 555363.92 | 93 | 1 | RAB13 | 5.54 |
| 187 | chr1 | 153917025 | 153964411 | 555363.92 | 93 | 1 | GATAD2B | 4.47 |
| 188 | chr1 | 153917025 | 153964411 | 555363.92 | 93 | 1 | CRTC2 | 4.40 |
| 189 | chr1 | 153917025 | 153964411 | 555363.92 | 93 | 1 | DENND4B | 4.27 |
| 190 | chr1 | 153917025 | 153964411 | 555363.92 | 93 | 1 | CREB3L4 | 3.28 |
| 191 | chr1 | 218542821 | 218577213 | 551303.76 | 94 | 1 | TGFB2 | 3.90 |
| 192 | chr2 | 134251481 | 134287022 | 550530.09 | 95 | 1 | na | na |
| 193 | chr8 | 131449235 | 131476224 | 545717.58 | 96 | 1 | ASAP1 | 4.90 |
| 194 | chr1 | 199117928 | 199147384 | 544052.32 | 97 | 1 | na | na |
| 195 | chr10 | 118918733 | 118935740 | 540482.46 | 98 | 1 | KIAA1598 | 3.78 |
| 196 | chr10 | 118918733 | 118935740 | 540482.46 | 98 | 1 | VAX1 | 3.06 |
| 197 | chr8 | 128805731 | 128832871 | 540357.40 | 99 | 1 | na | na |
| 198 | chr10 | 105541167 | 105581099 | 537085.40 | 100 | 1 | na | na |
| 199 | chr17 | 7013178 | 7041452 | 534095.86 | 101 | 1 | na | na |
| 200 | chr4 | 15001016 | 15023572 | 532998.28 | 102 | 1 | CPEB2 | 4.42 |
| 201 | chr3 | 194900079 | 194945959 | 531749.20 | 103 | 1 | na | na |
| 202 | chr1 | 51424959 | 51447302 | 521038.76 | 104 | 1 | CDKN2C | 4.82 |
| 203 | chr1 | 51424959 | 51447302 | 521038.76 | 104 | 1 | FAF1 | 3.64 |
| 204 | chr14 | 77489284 | 77512578 | 518058.56 | 105 | 1 | IRF2BPL | 5.07 |
| 205 | chr8 | 67418890 | 67478365 | 518027.25 | 106 | 1 | na | na |
| 206 | chr17 | 7108024 | 7156689 | 510982.50 | 107 | 1 | ACADVL | 7.36 |
| 207 | chr17 | 7108024 | 7156689 | 510982.50 | 107 | 1 | GABARAP | 6.30 |
| 208 | chr17 | 7108024 | 7156689 | 510982.50 | 107 | 1 | CTDNEP1 | 6.08 |
| 209 | chr17 | 7108024 | 7156689 | 510982.50 | 107 | 1 | DVL2 | 5.44 |
| 210 | chr17 | 7108024 | 7156689 | 510982.50 | 107 | 1 | ASGR1 | 5.03 |
| 211 | chr17 | 7108024 | 7156689 | 510982.50 | 107 | 1 | PHF23 | 4.79 |
| 212 | chr17 | 7108024 | 7156689 | 510982.50 | 107 | 1 | DLG4 | 4.44 |
| 213 | chr10 | 63795342 | 63845188 | 510423.04 | 108 | 1 | ARID5B | 3.73 |
| 214 | chr8 | 12601033 | 12624681 | 509614.40 | 109 | 1 | LONRF1 | 6.09 |
| 215 | chr19 | 49976788 | 50037595 | 509562.66 | 110 | 1 | RPS11 | 11.07 |
| 216 | chr19 | 49976788 | 50037595 | 509562.66 | 110 | 1 | RPL13A | 9.72 |
| 217 | chr19 | 49976788 | 50037595 | 509562.66 | 110 | 1 | RPL13AP5 | 8.79 |
| 218 | chr19 | 49976788 | 50037595 | 509562.66 | 110 | 1 | FCGRT | 7.67 |
| 219 | chr19 | 49976788 | 50037595 | 509562.66 | 110 | 1 | FLT3LG | 2.03 |
| 220 | chr19 | 49976788 | 50037595 | 509562.66 | 110 | 1 | SNORD35B | 1.82 |
| 221 | chr19 | 49976788 | 50037595 | 509562.66 | 110 | 1 | SNORD35A | 1.35 |
| 222 | chr19 | 49976788 | 50037595 | 509562.66 | 110 | 1 | SNORD32A | 1.04 |
| 223 | chr16 | 25792762 | 25824092 | 509425.80 | 111 | 1 | na | na |
| 224 | chr12 | 100882655 | 100948508 | 509043.69 | 112 | 1 | na | na |
| 225 | chr1 | 163529754 | 163567791 | 501327.66 | 113 | 1 | na | na |
| 226 | chr2 | 242742569 | 242806446 | 500795.68 | 114 | 1 | na | na |
| 227 | chr7 | 130691685 | 130725839 | 498306.86 | 115 | 1 | na | na |
| 228 | chr1 | 154927830 | 154956952 | 497694.98 | 116 | 1 | SHC1 | 6.00 |
| 229 | chr1 | 154927830 | 154956952 | 497694.98 | 116 | 1 | PMVK | 5.89 |
| 230 | chr1 | 154927830 | 154956952 | 497694.98 | 116 | 1 | CKS1B | 5.62 |
| 231 | chr1 | 154927830 | 154956952 | 497694.98 | 116 | 1 | PYGO2 | 5.31 |
| 232 | chr1 | 154927830 | 154956952 | 497694.98 | 116 | 1 | PBXIP1 | 4.84 |
| 233 | chr1 | 154927830 | 154956952 | 497694.98 | 116 | 1 | FLAD1 | 4.03 |
| 234 | chr1 | 154927830 | 154956952 | 497694.98 | 116 | 1 | ZBTB7B | 2.95 |
| 235 | chr20 | 21485946 | 21504350 | 497460.12 | 117 | 1 | NKX2-2 | 5.62 |
| 236 | chr11 | 134850225 | 134904163 | 497308.36 | 118 | 1 | na | na |
| 237 | chr1 | 25664361 | 25700955 | 492921.18 | 119 | 1 | TMEM50A | 6.63 |
| 238 | chr6 | 24321847 | 24361873 | 488717.46 | 120 | 1 | DCDC2 | 4.44 |
| 239 | chr2 | 223904634 | 223929635 | 487519.50 | 121 | 1 | KCNE4 | 6.07 |
| 240 | chr8 | 70779705 | 70806529 | 487392.08 | 122 | 1 | SLCO5A1 | 2.26 |
| 241 | chr12 | 12866940 | 12880202 | 478625.58 | 123 | 1 | CDKN1B | 4.83 |
| 242 | chr10 | 118889966 | 118899148 | 478290.38 | 124 | 1 | KIAA1598 | 3.78 |
| 243 | chr10 | 118889966 | 118899148 | 478290.38 | 124 | 1 | VAX1 | 3.06 |
| 244 | chr1 | 170630215 | 170646141 | 473957.76 | 125 | 1 | PRRX1 | 5.05 |
| 245 | chr2 | 145261880 | 145282854 | 471076.04 | 126 | 1 | ZEB2 | 3.80 |
| 246 | chr2 | 145261880 | 145282854 | 471076.04 | 126 | 1 | ZEB2-AS1 | 1.87 |
| 247 | chr19 | 13949152 | 13977449 | 470579.11 | 127 | 1 | na | na |
| 248 | chr9 | 89144921 | 89179529 | 470322.72 | 128 | 1 | na | na |
| 249 | chr8 | 127821023 | 127847152 | 470322.00 | 129 | 1 | na | na |
| 250 | chr10 | 72972182 | 73011261 | 468557.21 | 130 | 1 | UNC5B | 5.45 |
| 251 | chr14 | 22103034 | 22136783 | 465061.22 | 131 | 1 | na | na |
| 252 | chr21 | 42435242 | 42468720 | 464339.86 | 132 | 1 | na | na |
| 253 | chr8 | 29939347 | 29971859 | 464271.36 | 133 | 1 | TMEM66 | 6.55 |
| 254 | chr8 | 29939347 | 29971859 | 464271.36 | 133 | 1 | LEPROTL1 | 5.23 |
| 255 | chr15 | 40361115 | 40402006 | 464112.85 | 134 | 1 | BMF | 4.15 |
| 256 | chr2 | 1839057 | 1874755 | 462289.10 | 135 | 1 | na | na |
| 257 | chr17 | 8053904 | 8127659 | 461706.30 | 136 | 1 | AURKB | 5.66 |
| 258 | chr17 | 8053904 | 8127659 | 461706.30 | 136 | 1 | VAMP2 | 4.56 |
| 259 | chr17 | 8053904 | 8127659 | 461706.30 | 136 | 1 | C17orf59 | 2.53 |
| 260 | chr17 | 8053904 | 8127659 | 461706.30 | 136 | 1 | PER1 | 2.23 |
| 261 | chr17 | 8053904 | 8127659 | 461706.30 | 136 | 1 | TMEM107 | 2.16 |
| 262 | chr1 | 185741727 | 185787620 | 459847.86 | 137 | 1 | na | na |
| 263 | chr10 | 17229352 | 17273035 | 456050.52 | 138 | 1 | VIM | 8.86 |
| 264 | chr10 | 17229352 | 17273035 | 456050.52 | 138 | 1 | TRDMT1 | 1.27 |
| 265 | chr1 | 162253235 | 162281357 | 455013.96 | 139 | 1 | na | na |
| 266 | chr1 | 149847997 | 149872604 | 454245.22 | 140 | 1 | SF3B4 | 6.17 |
| 267 | chr1 | 149847997 | 149872604 | 454245.22 | 140 | 1 | SV2A | 4.77 |
| 268 | chr1 | 149847997 | 149872604 | 454245.22 | 140 | 1 | BOLA1 | 4.51 |
| 269 | chr1 | 149847997 | 149872604 | 454245.22 | 140 | 1 | HIST2H2AA4 | 2.32 |
| 270 | chr1 | 149847997 | 149872604 | 454245.22 | 140 | 1 | HIST2H2BC | 2.10 |
| 271 | chr1 | 149847997 | 149872604 | 454245.22 | 140 | 1 | HIST2H2AA3 | 2.02 |
| 272 | chr1 | 149847997 | 149872604 | 454245.22 | 140 | 1 | HIST2H2AC | 1.90 |
| 273 | chr1 | 149847997 | 149872604 | 454245.22 | 140 | 1 | HIST2H2BE | 1.35 |
| 274 | chr17 | 42263244 | 42300248 | 452928.96 | 141 | 1 | UBTF | 5.37 |
| 275 | chr17 | 42263244 | 42300248 | 452928.96 | 141 | 1 | ATXN7L3 | 4.44 |
| 276 | chr17 | 42263244 | 42300248 | 452928.96 | 141 | 1 | TMUB2 | 3.55 |
| 277 | chr15 | 60654227 | 60692816 | 450719.52 | 142 | 1 | ANXA2 | 7.76 |
| 278 | chr11 | 65184198 | 65198490 | 447053.76 | 143 | 1 | NEAT1 | 3.76 |
| 279 | chr11 | 65184198 | 65198490 | 447053.76 | 143 | 1 | FRMD8 | 3.52 |
| 280 | chr11 | 65184198 | 65198490 | 447053.76 | 143 | 1 | SLC25A45 | 1.21 |
| 281 | chr3 | 58215361 | 58239000 | 445831.54 | 144 | 1 | ABHD6 | 5.95 |
| 282 | chr15 | 78008841 | 78040948 | 445003.02 | 145 | 1 | na | na |
| 283 | chr11 | 61581749 | 61604483 | 444677.04 | 146 | 1 | FADS2 | 7.79 |
| 284 | chr11 | 61581749 | 61604483 | 444677.04 | 146 | 1 | FEN1 | 6.18 |
| 285 | chr11 | 61581749 | 61604483 | 444677.04 | 146 | 1 | FADS1 | 6.12 |
| 286 | chr11 | 61581749 | 61604483 | 444677.04 | 146 | 1 | MIR611 | 3.16 |
| 287 | chr11 | 10558710 | 10584567 | 443964.69 | 147 | 1 | RNF141 | 5.12 |
| 288 | chr20 | 42542813 | 42585192 | 440317.81 | 148 | 1 | TOX2 | 5.08 |
| 289 | chr8 | 65480275 | 65498629 | 438477.06 | 149 | 1 | na | na |
| 290 | chr6 | 148812529 | 148851977 | 437083.84 | 150 | 1 | na | na |
| 291 | chr8 | 21401763 | 21413522 | 436611.67 | 151 | 1 | na | na |
| 292 | chr3 | 145842957 | 145880748 | 433840.68 | 152 | 1 | PLOD2 | 4.40 |
| 293 | chr2 | 8816241 | 8834334 | 433508.28 | 153 | 1 | ID2 | 7.80 |
| 294 | chr11 | 114002554 | 114038108 | 432692.18 | 154 | 1 | na | na |
| 295 | chr11 | 34065011 | 34076427 | 431524.80 | 155 | 1 | CAPRIN1 | 6.61 |
| 296 | chr11 | 71095090 | 71110953 | 430839.08 | 156 | 1 | na | na |
| 297 | chr1 | 30979438 | 30999988 | 429700.50 | 157 | 1 | na | na |
| 298 | chr10 | 90733369 | 90763122 | 428740.73 | 158 | 1 | ACTA2 | 4.16 |
| 299 | chr10 | 90733369 | 90763122 | 428740.73 | 158 | 1 | FAS | 3.53 |
| 300 | chr16 | 70718649 | 70750960 | 427151.42 | 159 | 1 | MTSS1L | 6.00 |
| 301 | chr6 | 97886711 | 97919962 | 426610.33 | 160 | 1 | na | na |
| 302 | chr2 | 223153108 | 223171594 | 426102.30 | 161 | 1 | PAX3 | 4.49 |
| 303 | chr2 | 46525267 | 46563949 | 425502.00 | 162 | 1 | EPAS1 | 5.93 |
| 304 | chr1 | 202090479 | 202114936 | 425062.66 | 163 | 1 | ARL8A | 5.85 |
| 305 | chr6 | 10400415 | 10427887 | 423068.80 | 164 | 1 | TFAP2A | 3.80 |
| 306 | chr1 | 116891785 | 116921287 | 420108.48 | 165 | 1 | ATP1A1 | 7.38 |
| 307 | chr9 | 132368936 | 132439405 | 419995.24 | 166 | 1 | PRRX2 | 6.52 |
| 308 | chr9 | 132368936 | 132439405 | 419995.24 | 166 | 1 | ASB6 | 4.60 |
| 309 | chr9 | 132368936 | 132439405 | 419995.24 | 166 | 1 | NTMT1 | 3.89 |
| 310 | chr19 | 51220980 | 51249719 | 417002.89 | 167 | 1 | CLEC11A | 7.46 |
| 311 | chr19 | 51220980 | 51249719 | 417002.89 | 167 | 1 | SHANK1 | 4.88 |
| 312 | chr22 | 39151042 | 39201308 | 416705.14 | 168 | 1 | SUN2 | 5.21 |
| 313 | chr22 | 39151042 | 39201308 | 416705.14 | 168 | 1 | DNAL4 | 4.17 |
| 314 | chr2 | 213757141 | 213779282 | 414922.34 | 169 | 1 | na | na |
| 315 | chr8 | 2022617 | 2055462 | 413190.10 | 170 | 1 | na | na |
| 316 | chr20 | 3765792 | 3802280 | 411584.64 | 171 | 1 | CENPB | 5.62 |
| 317 | chr20 | 3765792 | 3802280 | 411584.64 | 171 | 1 | CDC25B | 5.00 |
| 318 | chr20 | 3765792 | 3802280 | 411584.64 | 171 | 1 | C20orf27 | 4.81 |
| 319 | chr20 | 3765792 | 3802280 | 411584.64 | 171 | 1 | MAVS | 4.03 |
| 320 | chr20 | 3765792 | 3802280 | 411584.64 | 171 | 1 | AP5S1 | 2.83 |
| 321 | chr8 | 37551774 | 37562167 | 411043.15 | 172 | 1 | ZNF703 | 6.50 |
| 322 | chr8 | 37551774 | 37562167 | 411043.15 | 172 | 1 | ERLIN2 | 3.79 |
| 323 | chr16 | 8617726 | 8630496 | 410938.60 | 173 | 1 | na | na |
| 324 | chr17 | 75434626 | 75474555 | 410070.83 | 174 | 1 | 41891 | 5.95 |
| 325 | chr4 | 116007770 | 116041108 | 410057.40 | 175 | 1 | NDST4 | 3.39 |
| 326 | chr19 | 59024550 | 59087813 | 409944.24 | 176 | 1 | TRIM28 | 7.58 |
| 327 | chr19 | 59024550 | 59087813 | 409944.24 | 176 | 1 | CHMP2A | 6.02 |
| 328 | chr19 | 59024550 | 59087813 | 409944.24 | 176 | 1 | UBE2M | 5.58 |
| 329 | chr19 | 59024550 | 59087813 | 409944.24 | 176 | 1 | ZBTB45 | 3.36 |
| 330 | chr19 | 59024550 | 59087813 | 409944.24 | 176 | 1 | MZF1 | 3.13 |
| 331 | chr1 | 248278117 | 248308779 | 409031.08 | 177 | 1 | na | na |
| 332 | chr8 | 49462838 | 49503767 | 408880.71 | 178 | 1 | na | na |
| 333 | chr8 | 127567847 | 127598980 | 408776.29 | 179 | 1 | FAM84B | 4.75 |
| 334 | chr3 | 47684931 | 47712736 | 407899.35 | 180 | 1 | na | na |
| 335 | chr14 | 85982133 | 86003716 | 407702.87 | 181 | 1 | FLRT2 | 5.10 |
| 336 | chr17 | 79314112 | 79339705 | 407696.49 | 182 | 1 | na | na |
| 337 | chr9 | 36921233 | 36945311 | 407158.98 | 183 | 1 | na | na |
| 338 | chr15 | 69357147 | 69380966 | 406352.14 | 184 | 1 | LINC00277 | 4.33 |
| 339 | chr19 | 12888284 | 12918395 | 405896.28 | 185 | 1 | PRDX2 | 7.53 |
| 340 | chr19 | 12888284 | 12918395 | 405896.28 | 185 | 1 | RNASEH2A | 6.23 |
| 341 | chr19 | 12888284 | 12918395 | 405896.28 | 185 | 1 | JUNB | 3.90 |
| 342 | chr19 | 12888284 | 12918395 | 405896.28 | 185 | 1 | HOOK2 | 2.87 |
| 343 | chr19 | 12888284 | 12918395 | 405896.28 | 185 | 1 | MAST1 | 1.31 |
| 344 | chr1 | 162352954 | 162369470 | 405632.96 | 186 | 1 | NOS1AP | 2.01 |
| 345 | chr8 | 144566651 | 144599072 | 404289.87 | 187 | 1 | ZC3H3 | 3.90 |
| 346 | chr14 | 76892980 | 76918330 | 402304.50 | 188 | 1 | na | na |
| 347 | chr1 | 28831355 | 28846288 | 401847.03 | 189 | 1 | SNHG3 | 5.18 |
| 348 | chr1 | 28831355 | 28846288 | 401847.03 | 189 | 1 | RCC1 | 4.78 |
| 349 | chr1 | 28831355 | 28846288 | 401847.03 | 189 | 1 | TRNAU1AP | 2.74 |
| 350 | chr10 | 29697914 | 29737786 | 401112.32 | 190 | 1 | na | na |
| 351 | chr1 | 236070893 | 236087814 | 400858.49 | 191 | 1 | na | na |
| 352 | chr1 | 23879596 | 23905390 | 400322.88 | 192 | 1 | ID3 | 6.84 |
| 353 | chr1 | 23879596 | 23905390 | 400322.88 | 192 | 1 | E2F2 | 3.48 |
| 354 | chr1 | 230162024 | 230181527 | 400201.56 | 193 | 1 | GALNT2 | 7.37 |
| 355 | chr2 | 152171471 | 152196946 | 399193.25 | 194 | 1 | TNFAIP6 | 5.25 |
| 356 | chr2 | 152171471 | 152196946 | 399193.25 | 194 | 1 | NMI | 4.34 |
| 357 | chr17 | 38587993 | 38638597 | 397241.40 | 195 | 1 | IGFBP4 | 8.19 |
| 358 | chr17 | 38587993 | 38638597 | 397241.40 | 195 | 1 | TOP2A | 7.13 |
| 359 | chr7 | 27191647 | 27214153 | 396555.72 | 196 | 1 | HOXA10 | 2.37 |
| 360 | chr7 | 27191647 | 27214153 | 396555.72 | 196 | 1 | HOXA7 | 1.48 |
| 361 | chr7 | 27191647 | 27214153 | 396555.72 | 196 | 1 | HOXA6 | 1.44 |
| 362 | chr7 | 27191647 | 27214153 | 396555.72 | 196 | 1 | HOXA9 | 1.37 |
| 363 | chr7 | 121939190 | 121952456 | 396255.42 | 197 | 1 | FEZF1-AS1 | 6.07 |
| 364 | chr7 | 121939190 | 121952456 | 396255.42 | 197 | 1 | FEZF1 | 5.09 |
| 365 | chr1 | 30722863 | 30737575 | 395899.92 | 198 | 1 | na | na |
| 366 | chr12 | 122231465 | 122243736 | 395126.20 | 199 | 1 | SETD1B | 3.61 |
| 367 | chr8 | 13049189 | 13083028 | 394901.13 | 200 | 1 | na | na |
| 368 | chr15 | 99394974 | 99419330 | 393592.96 | 201 | 1 | na | na |
| 369 | chr5 | 37809648 | 37841482 | 393149.90 | 202 | 1 | GDNF | 3.82 |
| 370 | chr20 | 1927998 | 1947183 | 392908.80 | 203 | 1 | na | na |
| 371 | chr8 | 9201997 | 9211768 | 391817.10 | 204 | 1 | na | na |
| 372 | chr1 | 150532799 | 150553352 | 389684.88 | 205 | 1 | MCL1 | 5.25 |
| 373 | chr18 | 55435973 | 55476607 | 389273.72 | 206 | 1 | ATP8B1 | 4.45 |
| 374 | chr8 | 119086040 | 119132858 | 388589.40 | 207 | 1 | EXT1 | 4.46 |
| 375 | chr1 | 234734483 | 234756465 | 387982.30 | 208 | 1 | IRF2BP2 | 4.78 |
| 376 | chr2 | 224585852 | 224615635 | 387774.66 | 209 | 1 | na | na |
| 377 | chr8 | 81776099 | 81806792 | 387652.59 | 210 | 1 | ZNF704 | 5.18 |
| 378 | chr1 | 156458923 | 156476306 | 384511.96 | 211 | 1 | MEF2D | 4.20 |
| 379 | chr7 | 3040141 | 3068825 | 384365.60 | 212 | 1 | CARD11 | 4.28 |
| 380 | chr8 | 77584997 | 77598445 | 384209.36 | 213 | 1 | ZFHX4 | 2.98 |
| 381 | chr1 | 32211370 | 32269289 | 381107.02 | 214 | 1 | BAI2 | 4.67 |
| 382 | chr13 | 46614267 | 46629013 | 380151.88 | 215 | 1 | ZC3H13 | 5.77 |
| 383 | chr13 | 46614267 | 46629013 | 380151.88 | 215 | 1 | CPB2-AS1 | 1.38 |
| 384 | chr13 | 30968406 | 30997248 | 380137.56 | 216 | 1 | na | na |
| 385 | chr11 | 110219158 | 110234598 | 378743.20 | 217 | 1 | na | na |
| 386 | chr12 | 98884473 | 98915919 | 378609.84 | 218 | 1 | TMPO | 5.69 |
| 387 | chr3 | 8351040 | 8366446 | 376984.82 | 219 | 1 | na | na |
| 388 | chrX | 2721164 | 2750893 | 376963.72 | 220 | 1 | GYG2 | 6.59 |
| 389 | chr5 | 24138673 | 24167862 | 376829.99 | 221 | 1 | na | na |
| 390 | chr6 | 135032713 | 135055591 | 374970.42 | 222 | 1 | na | na |
| 391 | chr11 | 73017636 | 73046660 | 374409.60 | 223 | 1 | ARHGEF17 | 4.02 |
| 392 | chr6 | 110603711 | 110620732 | 371398.22 | 224 | 1 | na | na |
| 393 | chr12 | 114909192 | 114920281 | 367822.13 | 225 | 1 | na | na |
| 394 | chr10 | 69833107 | 69882136 | 366246.63 | 226 | 1 | HERC4 | 2.89 |
| 395 | chr12 | 72660289 | 72676387 | 364941.66 | 227 | 1 | TRHDE | 3.41 |
| 396 | chr12 | 72660289 | 72676387 | 364941.66 | 227 | 1 | TRHDE-AS1 | 1.84 |
| 397 | chr11 | 33920144 | 33976413 | 363497.74 | 228 | 1 | LMO2 | 5.32 |
| 398 | chr6 | 134488730 | 134501243 | 361250.31 | 229 | 1 | SGK1 | 6.06 |
| 399 | chr8 | 99868029 | 99882080 | 359846.11 | 230 | 1 | STK3 | 3.13 |
| 400 | chr4 | 87461647 | 87482916 | 358808.03 | 231 | 1 | PTPN13 | 6.11 |
| 401 | chr4 | 174441807 | 174453682 | 358625.00 | 232 | 1 | HAND2 | 5.08 |
| 402 | chr1 | 198731690 | 198775572 | 356760.66 | 233 | 1 | na | na |
| 403 | chr2 | 216398826 | 216422299 | 353972.84 | 234 | 1 | na | na |
| 404 | chr1 | 223892363 | 223922876 | 352120.02 | 235 | 1 | CAPN2 | 5.26 |
| 405 | chr20 | 52196064 | 52241229 | 351835.35 | 236 | 1 | na | na |
| 406 | chr10 | 43890703 | 43916925 | 349539.26 | 237 | 1 | HNRNPF | 6.46 |
| 407 | chr8 | 2119916 | 2148046 | 349093.30 | 238 | 1 | na | na |
| 408 | chr22 | 50327316 | 50364640 | 347486.44 | 239 | 1 | PIM3 | 4.76 |
| 409 | chr22 | 50327316 | 50364640 | 347486.44 | 239 | 1 | ALG12 | 3.87 |
| 410 | chr22 | 50327316 | 50364640 | 347486.44 | 239 | 1 | CRELD2 | 3.52 |
| 411 | chr11 | 70097537 | 70119921 | 346504.32 | 240 | 1 | PPFIA1 | 5.09 |
| 412 | chr14 | 72177359 | 72225796 | 344871.44 | 241 | 1 | na | na |
| 413 | chr7 | 30360141 | 30394267 | 343990.08 | 242 | 1 | na | na |
| 414 | chr18 | 61030710 | 61048032 | 343841.70 | 243 | 1 | KDSR | 6.28 |
| 415 | chr1 | 234634206 | 234681763 | 340983.69 | 244 | 1 | TARBP1 | 1.59 |
| 416 | chr4 | 175122814 | 175140412 | 340873.26 | 245 | 1 | na | na |
| 417 | chr1 | 64783020 | 64830623 | 340837.48 | 246 | 1 | na | na |
| 418 | chr9 | 682539 | 707651 | 339012.00 | 247 | 1 | na | na |
| 419 | chr16 | 11438909 | 11459249 | 337644.00 | 248 | 1 | RMI2 | 3.49 |
| 420 | chr6 | 32935327 | 32941165 | 336560.70 | 249 | 1 | BRD2 | 5.65 |
| 421 | chr1 | 2120436 | 2162432 | 333448.24 | 250 | 1 | SKI | 5.12 |
| 422 | chr1 | 2120436 | 2162432 | 333448.24 | 250 | 1 | C1orf86 | 2.90 |
| 423 | chr11 | 67033356 | 67085648 | 333100.04 | 251 | 0 | ADRBK1 | 4.65 |
| 424 | chr11 | 67033356 | 67085648 | 333100.04 | 251 | 0 | SSH3 | 3.70 |
| 425 | chr11 | 67033356 | 67085648 | 333100.04 | 251 | 0 | ANKRD13D | 3.54 |
| 426 | chr20 | 1873478 | 1901976 | 332856.64 | 252 | 0 | SIRPA | 5.82 |
| 427 | chr2 | 172948856 | 172967824 | 332509.04 | 253 | 0 | DLX1 | 3.01 |
| 428 | chr2 | 172948856 | 172967824 | 332509.04 | 253 | 0 | DLX2 | 2.62 |
| 429 | chr11 | 83312387 | 83358004 | 331179.42 | 254 | 0 | na | na |
| 430 | chr13 | 94956324 | 94982664 | 330830.40 | 255 | 0 | na | na |
| 431 | chr14 | 69131071 | 69156121 | 328906.50 | 256 | 0 | na | na |
| 432 | chr15 | 45198534 | 45217896 | 327798.66 | 257 | 0 | na | na |
| 433 | chr18 | 46448869 | 46479668 | 327085.38 | 258 | 0 | SMAD7 | 2.79 |
| 434 | chr19 | 47609687 | 47617886 | 326730.15 | 259 | 0 | SAE1 | 6.39 |
| 435 | chr19 | 47609687 | 47617886 | 326730.15 | 259 | 0 | ZC3H4 | 4.34 |
| 436 | chr17 | 38256265 | 38280196 | 326418.84 | 260 | 0 | CASC3 | 5.19 |
| 437 | chr17 | 38256265 | 38280196 | 326418.84 | 260 | 0 | MSL1 | 4.81 |
| 438 | chr17 | 38256265 | 38280196 | 326418.84 | 260 | 0 | THRA | 3.40 |
| 439 | chr17 | 38256265 | 38280196 | 326418.84 | 260 | 0 | NR1D1 | 2.19 |
| 440 | chr20 | 21534675 | 21565765 | 324890.50 | 261 | 0 | na | na |
| 441 | chr20 | 21534675 | 21565765 | 324890.50 | 261 | 0 | na | na |
| 442 | chr3 | 193851787 | 193860867 | 323792.80 | 262 | 0 | HES1 | 5.70 |
| 443 | chr19 | 56135488 | 56167957 | 323066.55 | 263 | 0 | U2AF2 | 6.38 |
| 444 | chr19 | 56135488 | 56167957 | 323066.55 | 263 | 0 | EPN1 | 5.48 |
| 445 | chr19 | 56135488 | 56167957 | 323066.55 | 263 | 0 | ZNF581 | 4.37 |
| 446 | chr19 | 56135488 | 56167957 | 323066.55 | 263 | 0 | ZNF580 | 4.18 |
| 447 | chr19 | 56135488 | 56167957 | 323066.55 | 263 | 0 | CCDC106 | 4.16 |
| 448 | chr19 | 56135488 | 56167957 | 323066.55 | 263 | 0 | FIZ1 | 3.05 |
| 449 | chr19 | 56135488 | 56167957 | 323066.55 | 263 | 0 | ZNF784 | 2.62 |
| 450 | chr19 | 56135488 | 56167957 | 323066.55 | 263 | 0 | ZNF524 | 2.56 |
| 451 | chr1 | 214723250 | 214756128 | 322861.96 | 264 | 0 | CENPF | 6.41 |
| 452 | chr1 | 214723250 | 214756128 | 322861.96 | 264 | 0 | PTPN14 | 3.89 |
| 453 | chr10 | 32664698 | 32692977 | 322663.39 | 265 | 0 | EPC1 | 2.77 |
| 454 | chrX | 68504700 | 68523251 | 322045.36 | 266 | 0 | na | na |
| 455 | chr16 | 29800949 | 29828676 | 321633.20 | 267 | 0 | MAZ | 6.71 |
| 456 | chr16 | 29800949 | 29828676 | 321633.20 | 267 | 0 | KIF22 | 5.13 |
| 457 | chr16 | 29800949 | 29828676 | 321633.20 | 267 | 0 | PRRT2 | 3.99 |
| 458 | chr16 | 29800949 | 29828676 | 321633.20 | 267 | 0 | MVP | 2.51 |
| 459 | chr1 | 162628898 | 162667826 | 320766.72 | 268 | 0 | DDR2 | 5.82 |
| 460 | chr12 | 46764566 | 46803481 | 320659.60 | 269 | 0 | SLC38A2 | 6.24 |
| 461 | chr5 | 60602366 | 60643795 | 320246.17 | 270 | 0 | ZSWIM6 | 4.58 |
| 462 | chr2 | 69236158 | 69259209 | 320178.39 | 271 | 0 | ANTXR1 | 6.45 |
| 463 | chr8 | 37637533 | 37662649 | 319977.84 | 272 | 0 | GPR124 | 6.07 |
| 464 | chr8 | 37637533 | 37662649 | 319977.84 | 272 | 0 | PROSC | 4.13 |
| 465 | chr21 | 37842935 | 37866296 | 319812.09 | 273 | 0 | na | na |
| 466 | chr11 | 118778166 | 118801083 | 318087.96 | 274 | 0 | na | na |
| 467 | chr1 | 246728490 | 246758299 | 318062.03 | 275 | 0 | TFB2M | 4.21 |
| 468 | chr1 | 246728490 | 246758299 | 318062.03 | 275 | 0 | CNST | 4.07 |
| 469 | chr12 | 81666659 | 81691475 | 317892.96 | 276 | 0 | na | na |
| 470 | chr14 | 103045776 | 103070248 | 317891.28 | 277 | 0 | RCOR1 | 6.78 |
| 471 | chr4 | 3108421 | 3129004 | 316360.71 | 278 | 0 | HTT | 4.73 |
| 472 | chr18 | 23562031 | 23576715 | 316146.52 | 279 | 0 | na | na |
| 473 | chr17 | 46674485 | 46692172 | 315712.95 | 280 | 0 | HOXB5 | 2.95 |
| 474 | chr17 | 46674485 | 46692172 | 315712.95 | 280 | 0 | HOXB6 | 2.69 |
| 475 | chr17 | 46674485 | 46692172 | 315712.95 | 280 | 0 | HOXB3 | 2.29 |
| 476 | chr17 | 46674485 | 46692172 | 315712.95 | 280 | 0 | HOXB4 | 1.95 |
| 477 | chr17 | 46674485 | 46692172 | 315712.95 | 280 | 0 | HOXB-AS3 | 1.72 |
| 478 | chr17 | 46674485 | 46692172 | 315712.95 | 280 | 0 | HOXB9 | 1.39 |
| 479 | chr19 | 1847389 | 1885873 | 315568.80 | 281 | 0 | KLF16 | 4.10 |
| 480 | chr19 | 1847389 | 1885873 | 315568.80 | 281 | 0 | SCAMP4 | 4.04 |
| 481 | chr19 | 1847389 | 1885873 | 315568.80 | 281 | 0 | REXO1 | 3.57 |
| 482 | chr19 | 1847389 | 1885873 | 315568.80 | 281 | 0 | ADAT3 | 2.43 |
| 483 | chrX | 19135777 | 19147286 | 315461.69 | 282 | 0 | GPR64 | 6.33 |
| 484 | chr11 | 1038033 | 1052465 | 315339.20 | 283 | 0 | na | na |
| 485 | chr1 | 78872952 | 78893657 | 315130.10 | 284 | 0 | na | na |
| 486 | chr20 | 4223172 | 4232717 | 314698.65 | 285 | 0 | ADRA1D | 6.46 |
| 487 | chr1 | 20186817 | 20210559 | 314344.08 | 286 | 0 | OTUD3 | 3.30 |
| 488 | chr1 | 172875091 | 172911496 | 314175.15 | 287 | 0 | na | na |
| 489 | chr8 | 9532422 | 9561847 | 312787.75 | 288 | 0 | na | na |
| 490 | chr3 | 193593678 | 193620955 | 312594.42 | 289 | 0 | na | na |
| 491 | chr8 | 12973164 | 13016126 | 312333.74 | 290 | 0 | DLC1 | 5.02 |
| 492 | chr1 | 151019515 | 151044838 | 311726.13 | 291 | 0 | MLLT11 | 5.01 |
| 493 | chr1 | 151019515 | 151044838 | 311726.13 | 291 | 0 | CDC42SE1 | 4.96 |
| 494 | chr1 | 151019515 | 151044838 | 311726.13 | 291 | 0 | GABPB2 | 2.52 |
| 495 | chr1 | 151019515 | 151044838 | 311726.13 | 291 | 0 | C1orf56 | 2.36 |
| 496 | chr6 | 139685669 | 139697938 | 311387.22 | 292 | 0 | CITED2 | 7.56 |
| 497 | chrX | 19189909 | 19203213 | 310382.32 | 293 | 0 | na | na |
| 498 | chr20 | 37061529 | 37085593 | 309703.68 | 294 | 0 | RALGAPB | 3.56 |
| 499 | chr20 | 37061529 | 37085593 | 309703.68 | 294 | 0 | SNHG11 | 2.96 |
| 500 | chr20 | 37061529 | 37085593 | 309703.68 | 294 | 0 | SNORA60 | 1.14 |
| 501 | chr20 | 37061529 | 37085593 | 309703.68 | 294 | 0 | SNORA71C | 1.14 |
| 502 | chr19 | 3345916 | 3370977 | 309252.74 | 295 | 0 | NFIC | 5.32 |
| 503 | chr18 | 3446247 | 3458110 | 308319.37 | 296 | 0 | TGIF1 | 3.30 |
| 504 | chr9 | 136423293 | 136452171 | 306395.58 | 297 | 0 | na | na |
| 505 | chr6 | 31761982 | 31804451 | 305776.80 | 298 | 0 | C6orf48 | 6.13 |
| 506 | chr6 | 31761982 | 31804451 | 305776.80 | 298 | 0 | VARS | 5.51 |
| 507 | chr6 | 31761982 | 31804451 | 305776.80 | 298 | 0 | HSPA1B | 5.23 |
| 508 | chr6 | 31761982 | 31804451 | 305776.80 | 298 | 0 | LSM2 | 5.08 |
| 509 | chr6 | 31761982 | 31804451 | 305776.80 | 298 | 0 | NEU1 | 3.38 |
| 510 | chr6 | 31761982 | 31804451 | 305776.80 | 298 | 0 | SNORD48 | 2.43 |
| 511 | chr1 | 230201643 | 230223242 | 304977.88 | 299 | 0 | GALNT2 | 7.37 |
| 512 | chr10 | 71689505 | 71702528 | 304868.43 | 300 | 0 | na | na |
| 513 | chr21 | 15577580 | 15592666 | 304586.34 | 301 | 0 | RBM11 | 5.16 |
| 514 | chr21 | 15577580 | 15592666 | 304586.34 | 301 | 0 | LIPI | 3.79 |
| 515 | chr17 | 27037180 | 27056501 | 303919.33 | 302 | 0 | RPL23A | 7.51 |
| 516 | chr17 | 27037180 | 27056501 | 303919.33 | 302 | 0 | RAB34 | 5.83 |
| 517 | chr17 | 27037180 | 27056501 | 303919.33 | 302 | 0 | TRAF4 | 3.25 |
| 518 | chr17 | 27037180 | 27056501 | 303919.33 | 302 | 0 | TLCD1 | 2.07 |
| 519 | chr17 | 27037180 | 27056501 | 303919.33 | 302 | 0 | PROCA1 | 1.82 |
| 520 | chr17 | 27037180 | 27056501 | 303919.33 | 302 | 0 | NEK8 | 1.06 |
| 521 | chr3 | 39188557 | 39222586 | 303538.68 | 303 | 0 | CSRNP1 | 2.63 |
| 522 | chr1 | 181041323 | 181075822 | 302901.22 | 304 | 0 | IER5 | 3.68 |
| 523 | chr7 | 81995789 | 82025152 | 302438.90 | 305 | 0 | na | na |
| 524 | chr9 | 118352133 | 118379811 | 301966.98 | 306 | 0 | na | na |
| 525 | chr2 | 234595204 | 234620282 | 300434.44 | 307 | 0 | na | na |
| 526 | chr2 | 234224954 | 234243738 | 300168.32 | 308 | 0 | DGKD | 4.37 |
| 527 | chr3 | 128203002 | 128217038 | 299949.32 | 309 | 0 | GATA2 | 4.85 |
| 528 | chr18 | 3246201 | 3264195 | 299780.04 | 310 | 0 | MYL12B | 8.09 |
| 529 | chr18 | 3246201 | 3264195 | 299780.04 | 310 | 0 | MYL12A | 7.06 |
| 530 | chr17 | 43970643 | 43986072 | 298551.15 | 311 | 0 | MAPT | 5.31 |
| 531 | chr7 | 141040850 | 141063779 | 297618.42 | 312 | 0 | na | na |
| 532 | chr4 | 13628729 | 13673543 | 297564.96 | 313 | 0 | BOD1L1 | 3.05 |
| 533 | chr4 | 87879905 | 87904675 | 297487.70 | 314 | 0 | AFF1 | 4.67 |
| 534 | chr1 | 145454224 | 145479494 | 296669.80 | 315 | 0 | RBM8A | 7.01 |
| 535 | chr1 | 145454224 | 145479494 | 296669.80 | 315 | 0 | LIX1L | 5.24 |
| 536 | chr1 | 145454224 | 145479494 | 296669.80 | 315 | 0 | POLR3GL | 4.96 |
| 537 | chr1 | 145454224 | 145479494 | 296669.80 | 315 | 0 | PEX11B | 4.27 |
| 538 | chr1 | 145454224 | 145479494 | 296669.80 | 315 | 0 | TXNIP | 3.38 |
| 539 | chr1 | 145454224 | 145479494 | 296669.80 | 315 | 0 | ANKRD34A | 2.86 |
| 540 | chr12 | 80972316 | 81002351 | 296445.45 | 316 | 0 | na | na |
| 541 | chr10 | 63732688 | 63780736 | 295975.68 | 317 | 0 | na | na |
| 542 | chrX | 52679092 | 52697136 | 294297.64 | 318 | 0 | SSX7 | 2.94 |
| 543 | chr17 | 7783896 | 7793162 | 294195.50 | 319 | 0 | CHD3 | 7.63 |
| 544 | chr17 | 7783896 | 7793162 | 294195.50 | 319 | 0 | TRAPPC1 | 6.86 |
| 545 | chr17 | 7783896 | 7793162 | 294195.50 | 319 | 0 | CNTROB | 4.04 |
| 546 | chr17 | 7783896 | 7793162 | 294195.50 | 319 | 0 | KCNAB3 | 3.23 |
| 547 | chr17 | 7783896 | 7793162 | 294195.50 | 319 | 0 | LSMD1 | 3.12 |
| 548 | chr17 | 7783896 | 7793162 | 294195.50 | 319 | 0 | KDM6B | 2.57 |
| 549 | chr17 | 7783896 | 7793162 | 294195.50 | 319 | 0 | CYB5D1 | 2.43 |
| 550 | chr1 | 185802965 | 185833465 | 293715.00 | 320 | 0 | na | na |
| 551 | chr1 | 44315121 | 44335188 | 293580.21 | 321 | 0 | na | na |
| 552 | chr6 | 159270274 | 159291991 | 292962.33 | 322 | 0 | EZR | 6.38 |
| 553 | chr2 | 176986662 | 177006113 | 292737.55 | 323 | 0 | HOXD8 | 3.00 |
| 554 | chr2 | 176986662 | 177006113 | 292737.55 | 323 | 0 | HOXD9 | 2.98 |
| 555 | chr2 | 176986662 | 177006113 | 292737.55 | 323 | 0 | HOXD11 | 2.26 |
| 556 | chr2 | 176986662 | 177006113 | 292737.55 | 323 | 0 | HOXD13 | 1.90 |
| 557 | chr1 | 224682353 | 224698241 | 292339.20 | 324 | 0 | na | na |
| 558 | chr5 | 38709488 | 38726899 | 291634.25 | 325 | 0 | na | na |
| 559 | chr5 | 92899177 | 92922283 | 290673.48 | 326 | 0 | NR2F1 | 4.46 |
| 560 | chr6 | 2970276 | 3000799 | 290578.96 | 327 | 0 | SERPINB6 | 4.22 |
| 561 | chr6 | 2970276 | 3000799 | 290578.96 | 327 | 0 | NQO2 | 3.44 |
| 562 | chr8 | 9940544 | 9967254 | 290337.70 | 328 | 0 | MSRA | 3.33 |
| 563 | chr8 | 128737985 | 128752119 | 289464.32 | 329 | 0 | MYC | 6.26 |
| 564 | chr19 | 39881002 | 39905011 | 289308.45 | 330 | 0 | RPS16 | 9.75 |
| 565 | chr19 | 39881002 | 39905011 | 289308.45 | 330 | 0 | SUPT5H | 5.60 |
| 566 | chr19 | 39881002 | 39905011 | 289308.45 | 330 | 0 | PAF1 | 5.07 |
| 567 | chr19 | 39881002 | 39905011 | 289308.45 | 330 | 0 | MED29 | 3.82 |
| 568 | chr19 | 39881002 | 39905011 | 289308.45 | 330 | 0 | PLEKHG2 | 3.63 |
| 569 | chr19 | 39881002 | 39905011 | 289308.45 | 330 | 0 | ZFP36 | 3.17 |
| 570 | chr21 | 33888572 | 33911999 | 289089.18 | 331 | 0 | na | na |
| 571 | chr1 | 245126394 | 245136021 | 288810.00 | 332 | 0 | na | na |
| 572 | chr1 | 209740504 | 209774300 | 288617.84 | 333 | 0 | CAMK1G | 5.19 |
| 573 | chr7 | 128363887 | 128380821 | 287708.66 | 334 | 0 | CALU | 5.93 |
| 574 | chr1 | 145012144 | 145035929 | 287322.80 | 335 | 0 | PDE4DIP | 3.77 |
| 575 | chr20 | 52352281 | 52392102 | 286312.99 | 336 | 0 | na | na |
| 576 | chr1 | 170035392 | 170067744 | 285668.16 | 337 | 0 | KIFAP3 | 4.37 |
| 577 | chr1 | 245025457 | 245030795 | 284408.64 | 338 | 0 | HNRNPU | 7.20 |
| 578 | chr1 | 245025457 | 245030795 | 284408.64 | 338 | 0 | COX20 | 4.38 |
| 579 | chr9 | 34636776 | 34666684 | 283826.92 | 339 | 0 | SIGMAR1 | 6.02 |
| 580 | chr9 | 34636776 | 34666684 | 283826.92 | 339 | 0 | IL11RA | 5.29 |
| 581 | chr9 | 34636776 | 34666684 | 283826.92 | 339 | 0 | GALT | 4.28 |
| 582 | chr9 | 34636776 | 34666684 | 283826.92 | 339 | 0 | RPP25L | 3.79 |
| 583 | chr9 | 34636776 | 34666684 | 283826.92 | 339 | 0 | DCTN3 | 3.00 |
| 584 | chr16 | 49866676 | 49891059 | 283818.12 | 340 | 0 | na | na |
| 585 | chr1 | 149887581 | 149913117 | 283704.96 | 341 | 0 | SF3B4 | 6.17 |
| 586 | chr1 | 149887581 | 149913117 | 283704.96 | 341 | 0 | SV2A | 4.77 |
| 587 | chr1 | 149887581 | 149913117 | 283704.96 | 341 | 0 | BOLA1 | 4.51 |
| 588 | chr1 | 149887581 | 149913117 | 283704.96 | 341 | 0 | HIST2H2AC | 1.90 |
| 589 | chr1 | 149887581 | 149913117 | 283704.96 | 341 | 0 | HIST2H2BE | 1.35 |
| 590 | chr8 | 42395621 | 42411807 | 283255.00 | 342 | 0 | SLC20A2 | 3.26 |
| 591 | chr5 | 95055238 | 95070846 | 282816.96 | 343 | 0 | RHOBTB3 | 4.82 |
| 592 | chr12 | 14517895 | 14558211 | 282212.00 | 344 | 0 | ATF7IP | 3.23 |
| 593 | chr8 | 130456385 | 130464895 | 282191.60 | 345 | 0 | na | na |
| 594 | chr1 | 145381082 | 145414101 | 281982.26 | 346 | 0 | TXNIP | 3.38 |
| 595 | chr4 | 17092270 | 17102092 | 280909.20 | 347 | 0 | na | na |
| 596 | chr7 | 105317132 | 105348755 | 280812.24 | 348 | 0 | ATXN7L1 | 2.38 |
| 597 | chr1 | 228326856 | 228354797 | 280248.23 | 349 | 0 | GUK1 | 5.10 |
| 598 | chr1 | 228326856 | 228354797 | 280248.23 | 349 | 0 | MRPL55 | 4.46 |
| 599 | chr1 | 228326856 | 228354797 | 280248.23 | 349 | 0 | C1orf35 | 3.60 |
| 600 | chr1 | 228326856 | 228354797 | 280248.23 | 349 | 0 | IBA57 | 1.84 |
| 601 | chr2 | 232560571 | 232576181 | 280199.50 | 350 | 0 | PTMA | 8.88 |
| 602 | chr8 | 145007431 | 145028300 | 280061.98 | 351 | 0 | GRINA | 6.19 |
| 603 | chr8 | 145007431 | 145028300 | 280061.98 | 351 | 0 | PLEC | 5.73 |
| 604 | chr8 | 145007431 | 145028300 | 280061.98 | 351 | 0 | PARP10 | 1.11 |
| 605 | chr2 | 219259978 | 219272288 | 280052.50 | 352 | 0 | CTDSP1 | 4.95 |
| 606 | chr2 | 85473344 | 85505406 | 279580.64 | 353 | 0 | na | na |
| 607 | chr9 | 125789212 | 125813221 | 279464.76 | 354 | 0 | na | na |
| 608 | chr4 | 170533018 | 170563834 | 279192.96 | 355 | 0 | CLCN3 | 4.37 |
| 609 | chr4 | 170533018 | 170563834 | 279192.96 | 355 | 0 | NEK1 | 1.91 |
| 610 | chr8 | 12888513 | 12923376 | 278206.74 | 356 | 0 | na | na |
| 611 | chr14 | 77412640 | 77429284 | 276789.72 | 357 | 0 | na | na |
| 612 | chr8 | 125738286 | 125761036 | 276412.50 | 358 | 0 | MTSS1 | 4.35 |
| 613 | chr4 | 190935210 | 190943001 | 276190.95 | 359 | 0 | na | na |
| 614 | chr12 | 89719962 | 89748741 | 275415.03 | 360 | 0 | DUSP6 | 2.52 |
| 615 | chr6 | 84075010 | 84092016 | 275327.14 | 361 | 0 | na | na |
| 616 | chr18 | 3591684 | 3606801 | 274978.23 | 362 | 0 | na | na |
| 617 | chr1 | 114390064 | 114404180 | 274838.52 | 363 | 0 | RSBN1 | 2.91 |
| 618 | chr6 | 140363593 | 140374572 | 273926.05 | 364 | 0 | na | na |
| 619 | chr19 | 42246229 | 42258222 | 273680.26 | 365 | 0 | na | na |
| 620 | chr14 | 105940251 | 105958374 | 273294.84 | 366 | 0 | CRIP2 | 6.17 |
| 621 | chr14 | 105940251 | 105958374 | 273294.84 | 366 | 0 | C14orf80 | 3.23 |
| 622 | chr14 | 105940251 | 105958374 | 273294.84 | 366 | 0 | TMEM121 | 1.69 |
| 623 | chr1 | 182572442 | 182595809 | 272926.56 | 367 | 0 | RGS16 | 2.36 |
| 624 | chr18 | 56517608 | 56535100 | 272175.52 | 368 | 0 | ZNF532 | 5.08 |
| 625 | chr16 | 73081295 | 73093754 | 271855.38 | 369 | 0 | ZFHX3 | 2.72 |
| 626 | chr1 | 213596102 | 213607126 | 271851.84 | 370 | 0 | na | na |
| 627 | chr9 | 91347286 | 91383841 | 270872.55 | 371 | 0 | na | na |
| 628 | chr1 | 44226447 | 44249474 | 270567.25 | 372 | 0 | na | na |
| 629 | chr17 | 7451581 | 7488422 | 270412.94 | 373 | 0 | EIF4A1 | 8.37 |
| 630 | chr17 | 7451581 | 7488422 | 270412.94 | 373 | 0 | MPDU1 | 5.02 |
| 631 | chr17 | 7451581 | 7488422 | 270412.94 | 373 | 0 | SENP3 | 4.93 |
| 632 | chr17 | 7451581 | 7488422 | 270412.94 | 373 | 0 | FXR2 | 4.51 |
| 633 | chr17 | 7451581 | 7488422 | 270412.94 | 373 | 0 | SNORA48 | 1.81 |
| 634 | chr17 | 7451581 | 7488422 | 270412.94 | 373 | 0 | SNORD10 | 1.76 |
| 635 | chr17 | 7451581 | 7488422 | 270412.94 | 373 | 0 | CD68 | 1.63 |
| 636 | chr4 | 139131108 | 139165388 | 270126.40 | 374 | 0 | SLC7A11 | 2.91 |
| 637 | chr13 | 60655186 | 60678307 | 270053.28 | 375 | 0 | na | na |
| 638 | chr8 | 9408337 | 9419645 | 269017.32 | 376 | 0 | TNKS | 5.09 |
| 639 | chr18 | 44176176 | 44191568 | 268744.32 | 377 | 0 | na | na |
| 640 | chr6 | 5995693 | 6010004 | 267615.70 | 378 | 0 | NRN1 | 5.44 |
| 641 | chr11 | 36391707 | 36402222 | 267606.75 | 379 | 0 | PRR5L | 4.14 |
| 642 | chr4 | 55523217 | 55546792 | 267576.25 | 380 | 0 | KIT | 5.22 |
| 643 | chr6 | 27094083 | 27115795 | 267057.60 | 381 | 0 | HIST1H2BK | 3.29 |
| 644 | chr17 | 25659199 | 25684615 | 266613.84 | 382 | 0 | na | na |
| 645 | chr1 | 45264973 | 45286743 | 266029.40 | 383 | 0 | RPS8 | 8.57 |
| 646 | chr1 | 45264973 | 45286743 | 266029.40 | 383 | 0 | PLK3 | 1.69 |
| 647 | chr8 | 103800609 | 103823459 | 265974.00 | 384 | 0 | na | na |
| 648 | chr15 | 69108893 | 69114241 | 265528.20 | 385 | 0 | ANP32A | 5.44 |
| 649 | chr14 | 75724469 | 75746559 | 265080.00 | 386 | 0 | FOS | 2.59 |
| 650 | chr1 | 16160105 | 16176938 | 264614.76 | 387 | 0 | SPEN | 4.01 |
| 651 | chr11 | 111782494 | 111812834 | 263351.20 | 388 | 0 | DIXDC1 | 3.55 |
| 652 | chr11 | 111782494 | 111812834 | 263351.20 | 388 | 0 | C11orf1 | 1.19 |
| 653 | chr1 | 170554589 | 170564955 | 263192.74 | 389 | 0 | na | na |
| 654 | chr20 | 52772506 | 52791967 | 262334.28 | 390 | 0 | CYP24A1 | 4.58 |
| 655 | chr20 | 52772506 | 52791967 | 262334.28 | 390 | 0 | PFDN4 | 4.25 |
| 656 | chr11 | 62598415 | 62625438 | 261312.41 | 391 | 0 | SLC3A2 | 6.21 |
| 657 | chr11 | 62598415 | 62625438 | 261312.41 | 391 | 0 | SNHG1 | 5.84 |
| 658 | chr11 | 62598415 | 62625438 | 261312.41 | 391 | 0 | NXF1 | 4.18 |
| 659 | chr11 | 62598415 | 62625438 | 261312.41 | 391 | 0 | WDR74 | 4.01 |
| 660 | chr11 | 62598415 | 62625438 | 261312.41 | 391 | 0 | STX5 | 3.62 |
| 661 | chr11 | 62412926 | 62458132 | 261290.68 | 392 | 0 | GANAB | 6.97 |
| 662 | chr11 | 62412926 | 62458132 | 261290.68 | 392 | 0 | UBXN1 | 5.82 |
| 663 | chr11 | 62412926 | 62458132 | 261290.68 | 392 | 0 | B3GAT3 | 4.24 |
| 664 | chr11 | 62412926 | 62458132 | 261290.68 | 392 | 0 | INTS5 | 2.78 |
| 665 | chr11 | 62412926 | 62458132 | 261290.68 | 392 | 0 | SNORA57 | 2.62 |
| 666 | chr11 | 62412926 | 62458132 | 261290.68 | 392 | 0 | METTL12 | 1.73 |
| 667 | chr11 | 62412926 | 62458132 | 261290.68 | 392 | 0 | BSCL2 | 1.68 |
| 668 | chr13 | 100943582 | 100963671 | 261157.00 | 393 | 0 | na | na |
| 669 | chr1 | 27017800 | 27024968 | 260341.76 | 394 | 0 | ARID1A | 4.44 |
| 670 | chr1 | 244996084 | 245000386 | 259969.86 | 395 | 0 | HNRNPU | 7.20 |
| 671 | chr1 | 244996084 | 245000386 | 259969.86 | 395 | 0 | COX20 | 4.38 |
| 672 | chr1 | 16467953 | 16503924 | 259710.62 | 396 | 0 | EPHA2 | 3.47 |
| 673 | chr1 | 165147382 | 165159093 | 259632.87 | 397 | 0 | na | na |
| 674 | chr5 | 154419218 | 154435166 | 259314.48 | 398 | 0 | na | na |
| 675 | chr14 | 71781625 | 71789397 | 259273.92 | 399 | 0 | na | na |
| 676 | chr15 | 96873218 | 96901034 | 259245.12 | 400 | 0 | na | na |
| 677 | chr17 | 2295075 | 2305834 | 259076.72 | 401 | 0 | MNT | 3.38 |
| 678 | chr18 | 3645806 | 3671734 | 259020.72 | 402 | 0 | na | na |
| 679 | chr1 | 241863270 | 241882606 | 258715.68 | 403 | 0 | na | na |
| 680 | chr18 | 9968387 | 10007790 | 258483.68 | 404 | 0 | na | na |
| 681 | chr5 | 172362398 | 172387291 | 258389.34 | 405 | 0 | ATP6V0E1 | 5.13 |
| 682 | chr5 | 172362398 | 172387291 | 258389.34 | 405 | 0 | RPL26L1 | 4.63 |
| 683 | chr12 | 48202234 | 48216059 | 258112.75 | 406 | 0 | HDAC7 | 3.58 |
| 684 | chr12 | 48202234 | 48216059 | 258112.75 | 406 | 0 | SLC48A1 | 1.84 |
| 685 | chr1 | 183745265 | 183754296 | 257925.36 | 407 | 0 | na | na |
| 686 | chr2 | 183774397 | 183794485 | 257126.40 | 408 | 0 | na | na |
| 687 | chr1 | 172109805 | 172125368 | 256945.13 | 409 | 0 | DNM3OS | 2.58 |
| 688 | chr1 | 172109805 | 172125368 | 256945.13 | 409 | 0 | MIR214 | 1.77 |
| 689 | chr15 | 57425671 | 57448175 | 256095.52 | 410 | 0 | na | na |
| 690 | chr2 | 29473995 | 29505456 | 255777.93 | 411 | 0 | na | na |
| 691 | chr16 | 9277770 | 9295859 | 255235.79 | 412 | 0 | na | na |
| 692 | chr1 | 150120206 | 150136619 | 255222.15 | 413 | 0 | PLEKHO1 | 4.35 |
| 693 | chr18 | 56886688 | 56914634 | 255146.98 | 414 | 0 | GRP | 7.01 |
| 694 | chr10 | 112654545 | 112680679 | 255067.84 | 415 | 0 | PDCD4 | 4.91 |
| 695 | chr10 | 112654545 | 112680679 | 255067.84 | 415 | 0 | SHOC2 | 3.92 |
| 696 | chr10 | 112654545 | 112680679 | 255067.84 | 415 | 0 | BBIP1 | 2.94 |
| 697 | chr3 | 50357790 | 50397308 | 254891.10 | 416 | 0 | IFRD2 | 4.89 |
| 698 | chr3 | 50357790 | 50397308 | 254891.10 | 416 | 0 | HYAL2 | 4.34 |
| 699 | chr3 | 50357790 | 50397308 | 254891.10 | 416 | 0 | TMEM115 | 4.24 |
| 700 | chr3 | 50357790 | 50397308 | 254891.10 | 416 | 0 | RASSF1 | 3.98 |
| 701 | chr3 | 50357790 | 50397308 | 254891.10 | 416 | 0 | CYB561D2 | 3.93 |
| 702 | chr3 | 50357790 | 50397308 | 254891.10 | 416 | 0 | NPRL2 | 3.63 |
| 703 | chr3 | 50357790 | 50397308 | 254891.10 | 416 | 0 | TUSC2 | 3.30 |
| 704 | chr3 | 50357790 | 50397308 | 254891.10 | 416 | 0 | NAT6 | 2.71 |
| 705 | chr3 | 50357790 | 50397308 | 254891.10 | 416 | 0 | HYAL3 | 1.12 |
| 706 | chr1 | 225838598 | 225846753 | 254762.20 | 417 | 0 | ENAH | 4.91 |
| 707 | chr8 | 96971961 | 96984084 | 254461.77 | 418 | 0 | na | na |
| 708 | chr1 | 36598975 | 36627372 | 254437.12 | 419 | 0 | MAP7D1 | 5.15 |
| 709 | chr1 | 36598975 | 36627372 | 254437.12 | 419 | 0 | TRAPPC3 | 3.84 |
| 710 | chr5 | 180669474 | 180689622 | 254267.76 | 420 | 0 | GNB2L1 | 7.87 |
| 711 | chr5 | 180669474 | 180689622 | 254267.76 | 420 | 0 | TRIM41 | 3.54 |
| 712 | chr5 | 180669474 | 180689622 | 254267.76 | 420 | 0 | TRIM52 | 1.77 |
| 713 | chr1 | 163116271 | 163145651 | 253843.20 | 421 | 0 | na | na |
| 714 | chr8 | 21986631 | 22000314 | 253819.65 | 422 | 0 | REEP4 | 5.36 |
| 715 | chr8 | 21986631 | 22000314 | 253819.65 | 422 | 0 | HR | 4.91 |
| 716 | chr8 | 21986631 | 22000314 | 253819.65 | 422 | 0 | FAM160B2 | 4.78 |
| 717 | chr8 | 21986631 | 22000314 | 253819.65 | 422 | 0 | NUDT18 | 3.29 |
| 718 | chr1 | 165598989 | 165621039 | 253795.50 | 423 | 0 | MGST3 | 5.51 |
| 719 | chr16 | 74574342 | 74598068 | 253393.68 | 424 | 0 | na | na |
| 720 | chr20 | 45964532 | 45989892 | 253346.40 | 425 | 0 | na | na |
| 721 | chr8 | 144678315 | 144700932 | 252631.89 | 426 | 0 | EEF1D | 5.96 |
| 722 | chr8 | 144678315 | 144700932 | 252631.89 | 426 | 0 | TSTA3 | 5.54 |
| 723 | chr8 | 144678315 | 144700932 | 252631.89 | 426 | 0 | NAPRT1 | 4.80 |
| 724 | chr8 | 144678315 | 144700932 | 252631.89 | 426 | 0 | GSDMD | 4.66 |
| 725 | chr8 | 144678315 | 144700932 | 252631.89 | 426 | 0 | PYCRL | 3.68 |
| 726 | chr8 | 144678315 | 144700932 | 252631.89 | 426 | 0 | ZNF623 | 3.27 |
| 727 | chr10 | 22604360 | 22630858 | 252525.94 | 427 | 0 | BMI1 | 4.67 |
| 728 | chr10 | 22604360 | 22630858 | 252525.94 | 427 | 0 | COMMD3 | 3.92 |
| 729 | chr3 | 194025590 | 194054898 | 252341.88 | 428 | 0 | na | na |
| 730 | chr1 | 226248564 | 226252233 | 251876.85 | 429 | 0 | H3F3AP4 | 8.39 |
| 731 | chr1 | 226248564 | 226252233 | 251876.85 | 429 | 0 | H3F3A | 6.73 |
| 732 | chr17 | 4841847 | 4854151 | 251862.88 | 430 | 0 | PFN1 | 9.36 |
| 733 | chr17 | 4841847 | 4854151 | 251862.88 | 430 | 0 | SLC25A11 | 5.45 |
| 734 | chr17 | 4841847 | 4854151 | 251862.88 | 430 | 0 | RNF167 | 5.20 |
| 735 | chr17 | 4841847 | 4854151 | 251862.88 | 430 | 0 | SPAG7 | 4.18 |
| 736 | chr17 | 4841847 | 4854151 | 251862.88 | 430 | 0 | CAMTA2 | 3.28 |
| 737 | chr17 | 4841847 | 4854151 | 251862.88 | 430 | 0 | CHRNE | 1.30 |
| 738 | chr17 | 4841847 | 4854151 | 251862.88 | 430 | 0 | ENO3 | 1.07 |
| 739 | chr6 | 110494585 | 110508950 | 251818.45 | 431 | 0 | WASF1 | 6.55 |
| 740 | chr6 | 110494585 | 110508950 | 251818.45 | 431 | 0 | CDC40 | 4.40 |
| 741 | chr8 | 26466270 | 26495126 | 251624.32 | 432 | 0 | DPYSL2 | 6.99 |
| 742 | chr1 | 185280807 | 185293003 | 251359.56 | 433 | 0 | IVNS1ABP | 6.72 |
| 743 | chr9 | 75190407 | 75203860 | 251302.04 | 434 | 0 | na | na |
| 744 | chr18 | 70418421 | 70440057 | 251193.96 | 435 | 0 | na | na |
| 745 | chr1 | 52302448 | 52317902 | 251127.50 | 436 | 0 | NRD1 | 5.91 |
| 746 | chr11 | 65879016 | 65914975 | 250634.23 | 437 | 0 | na | na |
| 747 | chr1 | 150205452 | 150210543 | 250528.11 | 438 | 0 | ANP32E | 6.83 |
| 748 | chr1 | 150205452 | 150210543 | 250528.11 | 438 | 0 | APH1A | 6.14 |
| 749 | chr1 | 150205452 | 150210543 | 250528.11 | 438 | 0 | C1orf51 | 1.40 |
| 750 | chr1 | 150205452 | 150210543 | 250528.11 | 438 | 0 | C1orf54 | 1.12 |
| 751 | chr6 | 33167068 | 33182075 | 250016.62 | 439 | 0 | RING1 | 5.37 |
| 752 | chr6 | 33167068 | 33182075 | 250016.62 | 439 | 0 | SLC39A7 | 5.31 |
| 753 | chr6 | 33167068 | 33182075 | 250016.62 | 439 | 0 | RXRB | 4.36 |
| 754 | chr6 | 33167068 | 33182075 | 250016.62 | 439 | 0 | HSD17B8 | 3.78 |
| 755 | chr6 | 33167068 | 33182075 | 250016.62 | 439 | 0 | HCG25 | 3.52 |
| 756 | chr3 | 193493127 | 193507518 | 249827.76 | 440 | 0 | na | na |
| 757 | chr2 | 49103186 | 49120837 | 247820.04 | 441 | 0 | na | na |
| 758 | chr19 | 15608338 | 15621802 | 247333.68 | 442 | 0 | CYP4F22 | 6.96 |
| 759 | chr17 | 17725302 | 17745354 | 247241.16 | 443 | 0 | SREBF1 | 4.67 |
| 760 | chr8 | 48421306 | 48438202 | 247188.48 | 444 | 0 | na | na |
| 761 | chr8 | 76657122 | 76667343 | 246837.15 | 445 | 0 | na | na |
| 762 | chr1 | 170274680 | 170286307 | 246259.86 | 446 | 0 | na | na |
| 763 | chr4 | 7873533 | 7908244 | 245406.77 | 447 | 0 | na | na |
| 764 | chr3 | 45001416 | 45018696 | 245203.20 | 448 | 0 | ZDHHC3 | 4.35 |
| 765 | chr3 | 45001416 | 45018696 | 245203.20 | 448 | 0 | EXOSC7 | 4.23 |
| 766 | chr6 | 32143011 | 32164772 | 245028.86 | 449 | 0 | RNF5 | 5.27 |
| 767 | chr6 | 32143011 | 32164772 | 245028.86 | 449 | 0 | PBX2 | 5.00 |
| 768 | chr6 | 32143011 | 32164772 | 245028.86 | 449 | 0 | PPT2 | 3.98 |
| 769 | chr6 | 32143011 | 32164772 | 245028.86 | 449 | 0 | AGPAT1 | 3.75 |
| 770 | chr6 | 32143011 | 32164772 | 245028.86 | 449 | 0 | GPSM3 | 2.46 |
| 771 | chr6 | 32143011 | 32164772 | 245028.86 | 449 | 0 | PRRT1 | 1.22 |
| 772 | chr1 | 225600538 | 225617512 | 244934.82 | 450 | 0 | LBR | 5.91 |
| 773 | chr7 | 90247375 | 90255807 | 244780.96 | 451 | 0 | na | na |
| 774 | chr10 | 118846952 | 118857226 | 244726.68 | 452 | 0 | KIAA1598 | 3.78 |
| 775 | chr10 | 118846952 | 118857226 | 244726.68 | 452 | 0 | VAX1 | 3.06 |
| 776 | chr15 | 47689376 | 47717912 | 244268.16 | 453 | 0 | na | na |
| 777 | chr12 | 58238345 | 58264537 | 244109.44 | 454 | 0 | CTDSP2 | 5.94 |
| 778 | chr14 | 95981781 | 96002307 | 244054.14 | 455 | 0 | GLRX5 | 3.70 |
| 779 | chr14 | 95981781 | 96002307 | 244054.14 | 455 | 0 | SNHG10 | 1.87 |
| 780 | chr1 | 154971225 | 154990811 | 243845.70 | 456 | 0 | SHC1 | 6.00 |
| 781 | chr1 | 154971225 | 154990811 | 243845.70 | 456 | 0 | CKS1B | 5.62 |
| 782 | chr1 | 154971225 | 154990811 | 243845.70 | 456 | 0 | PYGO2 | 5.31 |
| 783 | chr1 | 154971225 | 154990811 | 243845.70 | 456 | 0 | ADAM15 | 4.77 |
| 784 | chr1 | 154971225 | 154990811 | 243845.70 | 456 | 0 | FLAD1 | 4.03 |
| 785 | chr1 | 154971225 | 154990811 | 243845.70 | 456 | 0 | ZBTB7B | 2.95 |
| 786 | chr2 | 27433863 | 27443960 | 243741.58 | 457 | 0 | CAD | 5.45 |
| 787 | chr2 | 27433863 | 27443960 | 243741.58 | 457 | 0 | SLC5A6 | 4.71 |
| 788 | chr2 | 27433863 | 27443960 | 243741.58 | 457 | 0 | SLC30A3 | 1.39 |
| 789 | chr15 | 74206123 | 74220601 | 243375.18 | 458 | 0 | LOXL1 | 5.27 |
| 790 | chr15 | 74206123 | 74220601 | 243375.18 | 458 | 0 | LOXL1-AS1 | 2.89 |
| 791 | chr3 | 55514901 | 55527445 | 243353.60 | 459 | 0 | WNT5A | 4.37 |
| 792 | chr22 | 27282742 | 27301072 | 243239.10 | 460 | 0 | na | na |
| 793 | chr11 | 125980871 | 126002274 | 242281.96 | 461 | 0 | na | na |
| 794 | chr20 | 48769278 | 48810135 | 241873.44 | 462 | 0 | CEBPB | 3.32 |
| 795 | chr20 | 48769278 | 48810135 | 241873.44 | 462 | 0 | TMEM189 | 2.69 |
| 796 | chr19 | 13260193 | 13276755 | 241142.72 | 463 | 0 | STX10 | 5.31 |
| 797 | chr19 | 13260193 | 13276755 | 241142.72 | 463 | 0 | NACC1 | 4.49 |
| 798 | chr19 | 13260193 | 13276755 | 241142.72 | 463 | 0 | TRMT1 | 4.43 |
| 799 | chr19 | 13260193 | 13276755 | 241142.72 | 463 | 0 | IER2 | 4.18 |
| 800 | chr9 | 125829032 | 125853102 | 240940.70 | 464 | 0 | na | na |
| 801 | chr7 | 18808695 | 18829749 | 240857.76 | 465 | 0 | na | na |
| 802 | chr10 | 104613537 | 104652625 | 240391.20 | 466 | 0 | AS3MT | 5.25 |
| 803 | chr10 | 104613537 | 104652625 | 240391.20 | 466 | 0 | C10orf32 | 3.71 |
| 804 | chr10 | 104613537 | 104652625 | 240391.20 | 466 | 0 | CNNM2 | 2.29 |
| 805 | chr19 | 49121356 | 49150141 | 239779.05 | 467 | 0 | RPL18 | 8.02 |
| 806 | chr19 | 49121356 | 49150141 | 239779.05 | 467 | 0 | CA11 | 3.61 |
| 807 | chr19 | 49121356 | 49150141 | 239779.05 | 467 | 0 | SPHK2 | 3.21 |
| 808 | chr19 | 49121356 | 49150141 | 239779.05 | 467 | 0 | DBP | 2.97 |
| 809 | chr3 | 194973191 | 194992709 | 239095.50 | 468 | 0 | XXYLT1 | 3.68 |
| 810 | chr2 | 70350221 | 70371010 | 239073.50 | 469 | 0 | PCBP1 | 7.28 |
| 811 | chr2 | 70350221 | 70371010 | 239073.50 | 469 | 0 | PCBP1-AS1 | 2.19 |
| 812 | chr1 | 151952224 | 151967287 | 238748.55 | 470 | 0 | S100A10 | 6.64 |
| 813 | chr1 | 151952224 | 151967287 | 238748.55 | 470 | 0 | S100A11 | 4.81 |
| 814 | chr20 | 47495005 | 47521792 | 238672.17 | 471 | 0 | ARFGEF2 | 3.52 |
| 815 | chr3 | 71627045 | 71635515 | 238599.90 | 472 | 0 | FOXP1 | 2.40 |
| 816 | chr1 | 224007000 | 224035152 | 238447.44 | 473 | 0 | TP53BP2 | 4.61 |
| 817 | chr9 | 118172992 | 118180361 | 238387.15 | 474 | 0 | na | na |
| 818 | chr20 | 47440984 | 47459574 | 238137.90 | 475 | 0 | PREX1 | 6.60 |
| 819 | chr8 | 38238150 | 38245319 | 238082.49 | 476 | 0 | WHSC1L1 | 3.91 |
| 820 | chr8 | 38238150 | 38245319 | 238082.49 | 476 | 0 | LETM2 | 1.17 |
| 821 | chr16 | 24986958 | 25006120 | 237800.42 | 477 | 0 | ARHGAP17 | 5.91 |
| 822 | chr3 | 197832761 | 197841446 | 237708.45 | 478 | 0 | na | na |
| 823 | chr19 | 34744751 | 34766300 | 237685.47 | 479 | 0 | KIAA0355 | 3.40 |
| 824 | chr12 | 96883019 | 96904480 | 237573.27 | 480 | 0 | na | na |
| 825 | chr12 | 96785202 | 96796188 | 237187.74 | 481 | 0 | na | na |
| 826 | chr11 | 62358321 | 62390516 | 236955.20 | 482 | 0 | GANAB | 6.97 |
| 827 | chr11 | 62358321 | 62390516 | 236955.20 | 482 | 0 | MTA2 | 5.64 |
| 828 | chr11 | 62358321 | 62390516 | 236955.20 | 482 | 0 | B3GAT3 | 4.24 |
| 829 | chr11 | 62358321 | 62390516 | 236955.20 | 482 | 0 | EML3 | 3.96 |
| 830 | chr11 | 62358321 | 62390516 | 236955.20 | 482 | 0 | TUT1 | 3.20 |
| 831 | chr11 | 62358321 | 62390516 | 236955.20 | 482 | 0 | INTS5 | 2.78 |
| 832 | chr19 | 13043523 | 13076901 | 236650.02 | 483 | 0 | CALR | 8.35 |
| 833 | chr19 | 13043523 | 13076901 | 236650.02 | 483 | 0 | NFIX | 6.83 |
| 834 | chr19 | 13043523 | 13076901 | 236650.02 | 483 | 0 | RAD23A | 6.35 |
| 835 | chr19 | 13043523 | 13076901 | 236650.02 | 483 | 0 | GADD45GIP1 | 6.35 |
| 836 | chr19 | 13043523 | 13076901 | 236650.02 | 483 | 0 | FARSA | 5.81 |
| 837 | chr17 | 43223992 | 43250358 | 236503.02 | 484 | 0 | HEXIM1 | 3.55 |
| 838 | chr17 | 43223992 | 43250358 | 236503.02 | 484 | 0 | HEXIM2 | 2.08 |
| 839 | chr17 | 43223992 | 43250358 | 236503.02 | 484 | 0 | ACBD4 | 1.91 |
| 840 | chr17 | 43223992 | 43250358 | 236503.02 | 484 | 0 | PLCD3 | 1.53 |
| 841 | chr8 | 90898308 | 90921308 | 236440.00 | 485 | 0 | OSGIN2 | 4.07 |
| 842 | chr12 | 52419059 | 52445975 | 236053.32 | 486 | 0 | C12orf44 | 3.34 |
| 843 | chr12 | 52419059 | 52445975 | 236053.32 | 486 | 0 | NR4A1 | 3.32 |
| 844 | chr11 | 118954880 | 118992877 | 235961.37 | 487 | 0 | H2AFX | 6.52 |
| 845 | chr11 | 118954880 | 118992877 | 235961.37 | 487 | 0 | HYOU1 | 4.59 |
| 846 | chr11 | 118954880 | 118992877 | 235961.37 | 487 | 0 | DPAGT1 | 4.15 |
| 847 | chr11 | 118954880 | 118992877 | 235961.37 | 487 | 0 | HMBS | 3.85 |
| 848 | chr11 | 118954880 | 118992877 | 235961.37 | 487 | 0 | VPS11 | 3.30 |
| 849 | chr11 | 118954880 | 118992877 | 235961.37 | 487 | 0 | C2CD2L | 2.37 |
| 850 | chr11 | 118954880 | 118992877 | 235961.37 | 487 | 0 | HINFP | 1.79 |
| 851 | chr2 | 223954238 | 223990415 | 235874.04 | 488 | 0 | na | na |
| 852 | chr18 | 39852909 | 39870377 | 234595.24 | 489 | 0 | na | na |
| 853 | chr3 | 147109467 | 147129948 | 234507.45 | 490 | 0 | ZIC1 | 2.74 |
| 854 | chr3 | 147109467 | 147129948 | 234507.45 | 490 | 0 | ZIC4 | 1.28 |
| 855 | chr5 | 16934243 | 16945965 | 234440.00 | 491 | 0 | MYO10 | 6.27 |
| 856 | chr1 | 17215034 | 17241403 | 234156.72 | 492 | 0 | na | na |
| 857 | chr2 | 7330279 | 7346132 | 234148.81 | 493 | 0 | na | na |
| 858 | chr7 | 99678496 | 99700736 | 233075.20 | 494 | 0 | MCM7 | 7.15 |
| 859 | chr7 | 99678496 | 99700736 | 233075.20 | 494 | 0 | COPS6 | 6.70 |
| 860 | chr7 | 99678496 | 99700736 | 233075.20 | 494 | 0 | TAF6 | 4.46 |
| 861 | chr7 | 99678496 | 99700736 | 233075.20 | 494 | 0 | AP4M1 | 4.10 |
| 862 | chr7 | 99678496 | 99700736 | 233075.20 | 494 | 0 | ZNF3 | 3.35 |
| 863 | chr7 | 99678496 | 99700736 | 233075.20 | 494 | 0 | CNPY4 | 2.70 |
| 864 | chr7 | 99678496 | 99700736 | 233075.20 | 494 | 0 | ZSCAN21 | 2.44 |
| 865 | chr7 | 99678496 | 99700736 | 233075.20 | 494 | 0 | MBLAC1 | 1.66 |
| 866 | chr16 | 75271665 | 75300694 | 232812.58 | 495 | 0 | BCAR1 | 3.79 |
| 867 | chr8 | 146010432 | 146026553 | 232787.24 | 496 | 0 | RPL8 | 9.91 |
| 868 | chr8 | 146010432 | 146026553 | 232787.24 | 496 | 0 | ZNF517 | 3.07 |
| 869 | chr8 | 146010432 | 146026553 | 232787.24 | 496 | 0 | ZNF251 | 2.93 |
| 870 | chr8 | 146010432 | 146026553 | 232787.24 | 496 | 0 | ZNF34 | 1.72 |
| 871 | chr8 | 146010432 | 146026553 | 232787.24 | 496 | 0 | ZNF7 | 1.32 |
| 872 | chr17 | 38459652 | 38484746 | 232621.38 | 497 | 0 | CDC6 | 4.70 |
| 873 | chr17 | 38459652 | 38484746 | 232621.38 | 497 | 0 | RARA | 3.01 |
| 874 | chr17 | 38459652 | 38484746 | 232621.38 | 497 | 0 | GJD3 | 2.63 |
| 875 | chr12 | 16738805 | 16762857 | 231620.76 | 498 | 0 | LMO3 | 2.74 |
| 876 | chr10 | 131744223 | 131770345 | 231179.70 | 499 | 0 | EBF3 | 3.72 |
| 877 | chr12 | 57471469 | 57484159 | 230958.00 | 500 | 0 | STAT6 | 4.69 |
| 878 | chr12 | 57471469 | 57484159 | 230958.00 | 500 | 0 | NAB2 | 4.09 |
| 879 | chr12 | 57471469 | 57484159 | 230958.00 | 500 | 0 | TMEM194A | 3.93 |
| 880 | chr12 | 57471469 | 57484159 | 230958.00 | 500 | 0 | LRP1 | 3.84 |
