# Supplementary material for: Functional, chemical genomic, and super-enhancer screening identify sensitivity to cyclin D1/CDK4 pathway inhibition in Ewing sarcoma
Source: Oncotarget. 2015 Aug 18;6(30):30178–93. doi: 10.18632/oncotarget.4903 (PMC4745789; doi:10.18632/oncotarget.4903)
Supplement: Supplementary file 3 [file oncotarget-06-30178-s003.doc]

| TC71  **Supplementary Table 2.**  List of actively transcribed target genes associated with the top 500 H3K27Ac enhancers in the TC71 Ewing sarcoma cell line. The enhancers are ranked based on their AUC signal. Enhancers with no actively transcribed target gene are marked by "na" in the Target Gene Symbol field. Super-enhancers rank from 1 to 138 and they target 239 actively transcribed genes.  # | Enhancer chr | Enhancer start | Enhancer end | Enhancer AUC | Enhancer  Rank | Enhancer Is SE? | Target Gene Symbol | Target Gene TC71 RPKM |
| --- | --- | --- | --- | --- | --- | --- | --- | --- |
| 1 | chr8 | 128805788 | 128948274 | 2224206.46 | 1 | 1 | PVT1 | 3.18 |
| 2 | chr8 | 129295010 | 129341236 | 2167999.40 | 2 | 1 | na | na |
| 3 | chr22 | 46431041 | 46486559 | 1779351.90 | 3 | 1 | na | na |
| 4 | chr17 | 79359303 | 79407988 | 1767752.35 | 4 | 1 | na | na |
| 5 | chr6 | 26087114 | 26175354 | 1347424.80 | 5 | 1 | HIST1H2AC | 4.37 |
| 6 | chr6 | 26087114 | 26175354 | 1347424.80 | 5 | 1 | HIST1H2BD | 3.80 |
| 7 | chr6 | 26087114 | 26175354 | 1347424.80 | 5 | 1 | HIST1H2BC | 2.02 |
| 8 | chr8 | 127377696 | 127441429 | 1244068.16 | 6 | 1 | na | na |
| 9 | chr1 | 185614225 | 185705261 | 1238089.60 | 7 | 1 | HMCN1 | 7.11 |
| 10 | chr8 | 127563988 | 127602104 | 1164062.64 | 8 | 1 | FAM84B | 7.34 |
| 11 | chr13 | 33732914 | 33805785 | 1137516.31 | 9 | 1 | STARD13 | 5.30 |
| 12 | chr8 | 12722631 | 12813221 | 1051749.90 | 10 | 1 | na | na |
| 13 | chr1 | 145053820 | 145118488 | 1021754.40 | 11 | 1 | SEC22B | 4.42 |
| 14 | chr1 | 145053820 | 145118488 | 1021754.40 | 11 | 1 | PDE4DIP | 2.61 |
| 15 | chr11 | 69430053 | 69490398 | 980002.80 | 12 | 1 | CCND1 | 7.67 |
| 16 | chr11 | 69430053 | 69490398 | 980002.80 | 12 | 1 | ORAOV1 | 2.48 |
| 17 | chr17 | 43970687 | 44036061 | 977341.30 | 13 | 1 | MAPT | 5.09 |
| 18 | chr17 | 47048796 | 47109684 | 931586.40 | 14 | 1 | IGF2BP1 | 5.02 |
| 19 | chr1 | 38454915 | 38537208 | 906045.93 | 15 | 1 | POU3F1 | 5.40 |
| 20 | chr1 | 38454915 | 38537208 | 906045.93 | 15 | 1 | SF3A3 | 4.82 |
| 21 | chr1 | 38454915 | 38537208 | 906045.93 | 15 | 1 | FHL3 | 3.74 |
| 22 | chr1 | 38454915 | 38537208 | 906045.93 | 15 | 1 | UTP11L | 3.34 |
| 23 | chr1 | 838905 | 881008 | 877847.55 | 16 | 1 | NOC2L | 5.91 |
| 24 | chr1 | 838905 | 881008 | 877847.55 | 16 | 1 | KLHL17 | 5.76 |
| 25 | chr1 | 65353166 | 65432877 | 868849.90 | 17 | 1 | JAK1 | 6.69 |
| 26 | chr14 | 97672612 | 97704855 | 752874.05 | 18 | 1 | na | na |
| 27 | chr8 | 27040642 | 27100478 | 724613.96 | 19 | 1 | na | na |
| 28 | chr11 | 83428448 | 83461601 | 713121.03 | 20 | 1 | na | na |
| 29 | chr8 | 127819860 | 127847104 | 672109.48 | 21 | 1 | na | na |
| 30 | chr19 | 1237412 | 1276913 | 660456.72 | 22 | 1 | CIRBP | 6.57 |
| 31 | chr19 | 1237412 | 1276913 | 660456.72 | 22 | 1 | ATP5D | 5.62 |
| 32 | chr19 | 1237412 | 1276913 | 660456.72 | 22 | 1 | MIDN | 4.45 |
| 33 | chr19 | 1237412 | 1276913 | 660456.72 | 22 | 1 | C19orf24 | 2.88 |
| 34 | chr19 | 1237412 | 1276913 | 660456.72 | 22 | 1 | C19orf26 | 1.71 |
| 35 | chr12 | 54348250 | 54422087 | 634998.20 | 23 | 1 | HOXC6 | 2.97 |
| 36 | chr12 | 54348250 | 54422087 | 634998.20 | 23 | 1 | HOXC12 | 2.55 |
| 37 | chr12 | 54348250 | 54422087 | 634998.20 | 23 | 1 | HOXC10 | 2.31 |
| 38 | chr12 | 54348250 | 54422087 | 634998.20 | 23 | 1 | HOXC4 | 1.62 |
| 39 | chr12 | 54348250 | 54422087 | 634998.20 | 23 | 1 | HOXC11 | 1.61 |
| 40 | chr12 | 54348250 | 54422087 | 634998.20 | 23 | 1 | HOXC9 | 1.45 |
| 41 | chr12 | 54348250 | 54422087 | 634998.20 | 23 | 1 | HOTAIR | 1.23 |
| 42 | chr13 | 100608435 | 100645094 | 620270.28 | 24 | 1 | ZIC2 | 4.38 |
| 43 | chr13 | 100608435 | 100645094 | 620270.28 | 24 | 1 | ZIC5 | 2.74 |
| 44 | chr14 | 102968025 | 103022180 | 591914.15 | 25 | 1 | ANKRD9 | 2.40 |
| 45 | chr9 | 15837448 | 15885620 | 589625.28 | 26 | 1 | na | na |
| 46 | chr8 | 128738098 | 128756540 | 574652.72 | 27 | 1 | MYC | 7.85 |
| 47 | chr14 | 42070888 | 42139064 | 552907.36 | 28 | 1 | LRFN5 | 3.23 |
| 48 | chr17 | 46792956 | 46808516 | 550201.60 | 29 | 1 | HOXB13 | 3.31 |
| 49 | chr1 | 144988869 | 145040149 | 527671.20 | 30 | 1 | na | na |
| 50 | chr13 | 46675509 | 46702115 | 523073.96 | 31 | 1 | na | na |
| 51 | chr19 | 42745935 | 42790637 | 518096.18 | 32 | 1 | ERF | 5.30 |
| 52 | chr19 | 42745935 | 42790637 | 518096.18 | 32 | 1 | PAFAH1B3 | 4.83 |
| 53 | chr19 | 42745935 | 42790637 | 518096.18 | 32 | 1 | CIC | 3.97 |
| 54 | chr19 | 42745935 | 42790637 | 518096.18 | 32 | 1 | GSK3A | 3.32 |
| 55 | chr19 | 42745935 | 42790637 | 518096.18 | 32 | 1 | ZNF526 | 2.12 |
| 56 | chr19 | 42745935 | 42790637 | 518096.18 | 32 | 1 | DEDD2 | 2.12 |
| 57 | chr19 | 42745935 | 42790637 | 518096.18 | 32 | 1 | TMEM145 | 1.52 |
| 58 | chr1 | 97431100 | 97470340 | 516398.40 | 33 | 1 | na | na |
| 59 | chr11 | 795673 | 860573 | 515955.00 | 34 | 1 | RPLP2 | 10.00 |
| 60 | chr11 | 795673 | 860573 | 515955.00 | 34 | 1 | POLR2L | 7.17 |
| 61 | chr11 | 795673 | 860573 | 515955.00 | 34 | 1 | CD151 | 6.94 |
| 62 | chr11 | 795673 | 860573 | 515955.00 | 34 | 1 | PNPLA2 | 5.83 |
| 63 | chr11 | 795673 | 860573 | 515955.00 | 34 | 1 | TSPAN4 | 5.40 |
| 64 | chr11 | 795673 | 860573 | 515955.00 | 34 | 1 | PIDD | 4.24 |
| 65 | chr11 | 795673 | 860573 | 515955.00 | 34 | 1 | SLC25A22 | 3.28 |
| 66 | chr11 | 795673 | 860573 | 515955.00 | 34 | 1 | SNORA52 | 2.86 |
| 67 | chr13 | 64642764 | 64688685 | 513855.99 | 35 | 1 | na | na |
| 68 | chr1 | 51424942 | 51448760 | 504703.42 | 36 | 1 | CDKN2C | 2.91 |
| 69 | chr1 | 51424942 | 51448760 | 504703.42 | 36 | 1 | FAF1 | 2.84 |
| 70 | chr19 | 49990162 | 50019336 | 493915.82 | 37 | 1 | RPS11 | 10.44 |
| 71 | chr19 | 49990162 | 50019336 | 493915.82 | 37 | 1 | RPL13A | 9.85 |
| 72 | chr19 | 49990162 | 50019336 | 493915.82 | 37 | 1 | RPL13AP5 | 9.13 |
| 73 | chr19 | 49990162 | 50019336 | 493915.82 | 37 | 1 | FCGRT | 8.31 |
| 74 | chr19 | 49990162 | 50019336 | 493915.82 | 37 | 1 | RCN3 | 6.18 |
| 75 | chr19 | 49990162 | 50019336 | 493915.82 | 37 | 1 | PIH1D1 | 5.40 |
| 76 | chr19 | 49990162 | 50019336 | 493915.82 | 37 | 1 | ALDH16A1 | 4.11 |
| 77 | chr19 | 49990162 | 50019336 | 493915.82 | 37 | 1 | SNORD35B | 2.98 |
| 78 | chr19 | 49990162 | 50019336 | 493915.82 | 37 | 1 | SNORD32A | 1.82 |
| 79 | chr19 | 49990162 | 50019336 | 493915.82 | 37 | 1 | SNORD35A | 1.53 |
| 80 | chr19 | 49990162 | 50019336 | 493915.82 | 37 | 1 | SNORD33 | 1.39 |
| 81 | chr10 | 21805594 | 21824937 | 493439.93 | 38 | 1 | MLLT10 | 2.90 |
| 82 | chr6 | 41594173 | 41625301 | 489954.72 | 39 | 1 | MDFI | 5.39 |
| 83 | chr1 | 23874949 | 23904689 | 488925.60 | 40 | 1 | ID3 | 5.73 |
| 84 | chr1 | 23874949 | 23904689 | 488925.60 | 40 | 1 | E2F2 | 3.06 |
| 85 | chr7 | 5460138 | 5470091 | 484611.57 | 41 | 1 | TNRC18 | 4.58 |
| 86 | chr8 | 70722881 | 70750625 | 477474.24 | 42 | 1 | SLCO5A1 | 6.04 |
| 87 | chr8 | 37637808 | 37685395 | 466828.47 | 43 | 1 | GPR124 | 5.70 |
| 88 | chr8 | 37637808 | 37685395 | 466828.47 | 43 | 1 | BRF2 | 3.31 |
| 89 | chr6 | 27773973 | 27808347 | 463361.52 | 44 | 1 | HIST1H4J | 1.18 |
| 90 | chr6 | 27773973 | 27808347 | 463361.52 | 44 | 1 | HIST1H4K | 1.14 |
| 91 | chr6 | 27773973 | 27808347 | 463361.52 | 44 | 1 | HIST1H2BN | 1.13 |
| 92 | chr6 | 27773973 | 27808347 | 463361.52 | 44 | 1 | HIST1H2AK | 1.06 |
| 93 | chr6 | 27773973 | 27808347 | 463361.52 | 44 | 1 | HIST1H3H | 1.00 |
| 94 | chr8 | 127297642 | 127325518 | 458838.96 | 45 | 1 | na | na |
| 95 | chr21 | 46327138 | 46360760 | 453897.00 | 46 | 1 | ITGB2 | 7.99 |
| 96 | chr21 | 46327138 | 46360760 | 453897.00 | 46 | 1 | FAM207A | 4.34 |
| 97 | chr21 | 46327138 | 46360760 | 453897.00 | 46 | 1 | C21orf67 | 2.86 |
| 98 | chr12 | 12866960 | 12880043 | 451232.67 | 47 | 1 | CDKN1B | 3.67 |
| 99 | chr12 | 12866960 | 12880043 | 451232.67 | 47 | 1 | APOLD1 | 1.62 |
| 100 | chr12 | 12866960 | 12880043 | 451232.67 | 47 | 1 | GPR19 | 1.49 |
| 101 | chr11 | 65243635 | 65267694 | 446535.04 | 48 | 1 | MALAT1 | 5.10 |
| 102 | chr11 | 65243635 | 65267694 | 446535.04 | 48 | 1 | SCYL1 | 4.19 |
| 103 | chr1 | 116891900 | 116921072 | 442830.96 | 49 | 1 | ATP1A1 | 10.19 |
| 104 | chr17 | 46671549 | 46704938 | 441402.58 | 50 | 1 | HOXB9 | 3.49 |
| 105 | chr17 | 46671549 | 46704938 | 441402.58 | 50 | 1 | HOXB6 | 2.91 |
| 106 | chr17 | 46671549 | 46704938 | 441402.58 | 50 | 1 | HOXB-AS3 | 2.26 |
| 107 | chr17 | 46671549 | 46704938 | 441402.58 | 50 | 1 | HOXB4 | 1.76 |
| 108 | chr17 | 46671549 | 46704938 | 441402.58 | 50 | 1 | HOXB3 | 1.44 |
| 109 | chr17 | 46671549 | 46704938 | 441402.58 | 50 | 1 | HOXB8 | 1.36 |
| 110 | chr2 | 176944772 | 176973189 | 440463.50 | 51 | 1 | HOXD13 | 4.59 |
| 111 | chr2 | 176944772 | 176973189 | 440463.50 | 51 | 1 | HOXD11 | 2.86 |
| 112 | chr2 | 176944772 | 176973189 | 440463.50 | 51 | 1 | EVX2 | 1.35 |
| 113 | chr11 | 74138317 | 74181332 | 438322.85 | 52 | 1 | KCNE3 | 4.39 |
| 114 | chr11 | 74138317 | 74181332 | 438322.85 | 52 | 1 | LIPT2 | 1.53 |
| 115 | chr10 | 26957997 | 26989148 | 435802.49 | 53 | 1 | PDSS1 | 3.98 |
| 116 | chr13 | 46615690 | 46636734 | 434558.60 | 54 | 1 | ZC3H13 | 6.47 |
| 117 | chr13 | 46615690 | 46636734 | 434558.60 | 54 | 1 | CPB2-AS1 | 2.08 |
| 118 | chr10 | 32659110 | 32692615 | 421827.95 | 55 | 1 | EPC1 | 3.25 |
| 119 | chr8 | 37549093 | 37562424 | 421392.91 | 56 | 1 | ZNF703 | 5.56 |
| 120 | chr1 | 201210031 | 201254923 | 417944.52 | 57 | 1 | PKP1 | 6.54 |
| 121 | chr17 | 74692690 | 74734995 | 415858.15 | 58 | 1 | SRSF2 | 7.50 |
| 122 | chr17 | 74692690 | 74734995 | 415858.15 | 58 | 1 | MXRA7 | 5.14 |
| 123 | chr17 | 74692690 | 74734995 | 415858.15 | 58 | 1 | MFSD11 | 4.20 |
| 124 | chr17 | 74692690 | 74734995 | 415858.15 | 58 | 1 | METTL23 | 3.80 |
| 125 | chr17 | 74692690 | 74734995 | 415858.15 | 58 | 1 | JMJD6 | 1.83 |
| 126 | chr17 | 74692690 | 74734995 | 415858.15 | 58 | 1 | MIR636 | 1.26 |
| 127 | chr2 | 145265105 | 145282703 | 414080.94 | 59 | 1 | ZEB2 | 3.39 |
| 128 | chr2 | 145265105 | 145282703 | 414080.94 | 59 | 1 | ZEB2-AS1 | 1.82 |
| 129 | chr7 | 116131780 | 116168629 | 413814.27 | 60 | 1 | CAV1 | 7.46 |
| 130 | chr7 | 116131780 | 116168629 | 413814.27 | 60 | 1 | CAV2 | 3.59 |
| 131 | chr1 | 28831484 | 28846339 | 413860.30 | 61 | 1 | SNHG3 | 6.55 |
| 132 | chr1 | 28831484 | 28846339 | 413860.30 | 61 | 1 | RCC1 | 4.65 |
| 133 | chr1 | 28831484 | 28846339 | 413860.30 | 61 | 1 | TRNAU1AP | 2.25 |
| 134 | chr6 | 140364808 | 140404527 | 413474.79 | 62 | 1 | na | na |
| 135 | chr14 | 105931983 | 105958227 | 407831.76 | 63 | 1 | CRIP2 | 5.82 |
| 136 | chr14 | 105931983 | 105958227 | 407831.76 | 63 | 1 | TMEM121 | 3.02 |
| 137 | chr14 | 105931983 | 105958227 | 407831.76 | 63 | 1 | C14orf80 | 2.42 |
| 138 | chr10 | 71798257 | 71817564 | 406991.56 | 64 | 1 | H2AFY2 | 6.53 |
| 139 | chr11 | 61581657 | 61604520 | 406046.88 | 65 | 1 | FADS2 | 7.33 |
| 140 | chr11 | 61581657 | 61604520 | 406046.88 | 65 | 1 | FADS1 | 6.25 |
| 141 | chr11 | 61581657 | 61604520 | 406046.88 | 65 | 1 | FEN1 | 5.82 |
| 142 | chr11 | 61581657 | 61604520 | 406046.88 | 65 | 1 | MIR611 | 2.45 |
| 143 | chr18 | 46428073 | 46479503 | 401668.30 | 66 | 1 | SMAD7 | 2.19 |
| 144 | chr15 | 40360882 | 40401938 | 401117.12 | 67 | 1 | BMF | 3.83 |
| 145 | chr18 | 10454328 | 10478484 | 397124.64 | 68 | 1 | APCDD1 | 7.42 |
| 146 | chr9 | 132242677 | 132263076 | 395944.59 | 69 | 1 | na | na |
| 147 | chr11 | 34065118 | 34076862 | 392836.80 | 70 | 1 | CAPRIN1 | 6.24 |
| 148 | chr19 | 56135499 | 56168361 | 392700.90 | 71 | 1 | U2AF2 | 6.13 |
| 149 | chr19 | 56135499 | 56168361 | 392700.90 | 71 | 1 | ZNF581 | 4.58 |
| 150 | chr19 | 56135499 | 56168361 | 392700.90 | 71 | 1 | EPN1 | 4.35 |
| 151 | chr19 | 56135499 | 56168361 | 392700.90 | 71 | 1 | ZNF580 | 3.91 |
| 152 | chr19 | 56135499 | 56168361 | 392700.90 | 71 | 1 | CCDC106 | 3.53 |
| 153 | chr19 | 56135499 | 56168361 | 392700.90 | 71 | 1 | ZNF784 | 2.23 |
| 154 | chr11 | 10556981 | 10581695 | 389245.50 | 72 | 1 | RNF141 | 4.38 |
| 155 | chr8 | 70769071 | 70789070 | 386780.66 | 73 | 1 | na | na |
| 156 | chr17 | 79437775 | 79457671 | 384589.68 | 74 | 1 | na | na |
| 157 | chr9 | 4652949 | 4680570 | 381169.80 | 75 | 1 | CDC37L1 | 2.56 |
| 158 | chr9 | 4652949 | 4680570 | 381169.80 | 75 | 1 | SPATA6L | 1.87 |
| 159 | chr18 | 61031350 | 61047919 | 380092.86 | 76 | 1 | KDSR | 7.31 |
| 160 | chr19 | 12885904 | 12918345 | 378910.88 | 77 | 1 | PRDX2 | 6.36 |
| 161 | chr19 | 12885904 | 12918345 | 378910.88 | 77 | 1 | RNASEH2A | 5.36 |
| 162 | chr19 | 12885904 | 12918345 | 378910.88 | 77 | 1 | JUNB | 3.45 |
| 163 | chr19 | 12885904 | 12918345 | 378910.88 | 77 | 1 | HOOK2 | 3.14 |
| 164 | chr19 | 12885904 | 12918345 | 378910.88 | 77 | 1 | MAST1 | 2.04 |
| 165 | chr19 | 13949281 | 13977291 | 374493.70 | 78 | 1 | na | na |
| 166 | chr11 | 62598420 | 62625723 | 373505.04 | 79 | 1 | SLC3A2 | 7.71 |
| 167 | chr11 | 62598420 | 62625723 | 373505.04 | 79 | 1 | SNHG1 | 7.54 |
| 168 | chr11 | 62598420 | 62625723 | 373505.04 | 79 | 1 | WDR74 | 4.85 |
| 169 | chr11 | 62598420 | 62625723 | 373505.04 | 79 | 1 | STX5 | 3.02 |
| 170 | chr19 | 18524984 | 18549221 | 373249.80 | 80 | 1 | ISYNA1 | 8.20 |
| 171 | chr19 | 18524984 | 18549221 | 373249.80 | 80 | 1 | SSBP4 | 6.63 |
| 172 | chr12 | 109231845 | 109256560 | 370230.70 | 81 | 1 | SSH1 | 3.21 |
| 173 | chr10 | 29697944 | 29737681 | 369951.47 | 82 | 1 | na | na |
| 174 | chr11 | 16624606 | 16641086 | 364372.80 | 83 | 1 | na | na |
| 175 | chr7 | 16741335 | 16795303 | 355649.12 | 84 | 1 | TSPAN13 | 8.25 |
| 176 | chr12 | 57471768 | 57489482 | 355697.12 | 85 | 1 | TMEM194A | 5.33 |
| 177 | chr12 | 57471768 | 57489482 | 355697.12 | 85 | 1 | LRP1 | 5.05 |
| 178 | chr12 | 57471768 | 57489482 | 355697.12 | 85 | 1 | STAT6 | 5.00 |
| 179 | chr12 | 57471768 | 57489482 | 355697.12 | 85 | 1 | NAB2 | 4.81 |
| 180 | chr11 | 65184357 | 65198079 | 354302.04 | 86 | 1 | FRMD8 | 3.31 |
| 181 | chr11 | 65184357 | 65198079 | 354302.04 | 86 | 1 | NEAT1 | 3.08 |
| 182 | chr11 | 65184357 | 65198079 | 354302.04 | 86 | 1 | SLC25A45 | 1.71 |
| 183 | chr13 | 34097071 | 34122013 | 352430.46 | 87 | 1 | na | na |
| 184 | chr2 | 176992721 | 177023513 | 352260.48 | 88 | 1 | HOXD8 | 2.33 |
| 185 | chr2 | 176992721 | 177023513 | 352260.48 | 88 | 1 | HOXD-AS2 | 2.06 |
| 186 | chr6 | 10401874 | 10435207 | 348996.51 | 89 | 1 | TFAP2A | 2.29 |
| 187 | chr8 | 81776106 | 81796774 | 347222.40 | 90 | 1 | ZNF704 | 4.22 |
| 188 | chr2 | 223904785 | 223928148 | 346473.29 | 91 | 1 | KCNE4 | 6.99 |
| 189 | chr7 | 18556841 | 18593943 | 346161.66 | 92 | 1 | HDAC9 | 2.23 |
| 190 | chr19 | 1847293 | 1885536 | 345716.72 | 93 | 1 | SCAMP4 | 4.91 |
| 191 | chr19 | 1847293 | 1885536 | 345716.72 | 93 | 1 | REXO1 | 3.56 |
| 192 | chr19 | 1847293 | 1885536 | 345716.72 | 93 | 1 | KLF16 | 3.23 |
| 193 | chr19 | 1847293 | 1885536 | 345716.72 | 93 | 1 | ADAT3 | 1.88 |
| 194 | chr3 | 44992054 | 45029372 | 345564.68 | 94 | 1 | EXOSC7 | 4.50 |
| 195 | chr3 | 44992054 | 45029372 | 345564.68 | 94 | 1 | ZDHHC3 | 4.05 |
| 196 | chr19 | 13260276 | 13279271 | 343999.45 | 95 | 1 | TRMT1 | 6.40 |
| 197 | chr19 | 13260276 | 13279271 | 343999.45 | 95 | 1 | IER2 | 4.95 |
| 198 | chr19 | 13260276 | 13279271 | 343999.45 | 95 | 1 | STX10 | 4.91 |
| 199 | chr19 | 13260276 | 13279271 | 343999.45 | 95 | 1 | NACC1 | 3.81 |
| 200 | chr9 | 14906689 | 14937644 | 340505.00 | 96 | 1 | FREM1 | 5.07 |
| 201 | chr15 | 78009439 | 78040034 | 340216.40 | 97 | 1 | na | na |
| 202 | chr20 | 4446179 | 4471895 | 339194.04 | 98 | 1 | na | na |
| 203 | chr17 | 79649851 | 79680224 | 335317.92 | 99 | 1 | HGS | 5.79 |
| 204 | chr17 | 79649851 | 79680224 | 335317.92 | 99 | 1 | ARL16 | 4.88 |
| 205 | chr17 | 79649851 | 79680224 | 335317.92 | 99 | 1 | SLC25A10 | 1.29 |
| 206 | chr17 | 79315119 | 79324556 | 334541.65 | 100 | 1 | na | na |
| 207 | chr8 | 125484255 | 125500582 | 333397.34 | 101 | 1 | RNF139 | 6.23 |
| 208 | chr8 | 125484255 | 125500582 | 333397.34 | 101 | 1 | TRMT12 | 3.36 |
| 209 | chr6 | 31762047 | 31804497 | 330685.50 | 102 | 1 | C6orf48 | 6.27 |
| 210 | chr6 | 31762047 | 31804497 | 330685.50 | 102 | 1 | VARS | 5.60 |
| 211 | chr6 | 31762047 | 31804497 | 330685.50 | 102 | 1 | HSPA1B | 5.16 |
| 212 | chr6 | 31762047 | 31804497 | 330685.50 | 102 | 1 | NEU1 | 4.76 |
| 213 | chr6 | 31762047 | 31804497 | 330685.50 | 102 | 1 | LSM2 | 4.20 |
| 214 | chr6 | 31762047 | 31804497 | 330685.50 | 102 | 1 | SNORD48 | 4.10 |
| 215 | chr14 | 97619365 | 97655754 | 330412.12 | 103 | 1 | na | na |
| 216 | chr4 | 175118707 | 175140170 | 329027.79 | 104 | 1 | na | na |
| 217 | chr17 | 80053340 | 80065245 | 328339.90 | 105 | 1 | FASN | 6.70 |
| 218 | chr17 | 80053340 | 80065245 | 328339.90 | 105 | 1 | DUS1L | 5.61 |
| 219 | chr17 | 80053340 | 80065245 | 328339.90 | 105 | 1 | RFNG | 5.20 |
| 220 | chr17 | 80053340 | 80065245 | 328339.90 | 105 | 1 | GPS1 | 5.00 |
| 221 | chr10 | 69833777 | 69880236 | 324283.82 | 106 | 1 | HERC4 | 2.09 |
| 222 | chr10 | 69833777 | 69880236 | 324283.82 | 106 | 1 | MYPN | 1.27 |
| 223 | chr14 | 97528660 | 97544148 | 324163.84 | 107 | 1 | na | na |
| 224 | chr8 | 112350658 | 112382583 | 312545.75 | 108 | 1 | na | na |
| 225 | chr16 | 67046690 | 67067535 | 311632.75 | 109 | 1 | CBFB | 4.08 |
| 226 | chr16 | 67046690 | 67067535 | 311632.75 | 109 | 1 | CES4A | 1.36 |
| 227 | chr10 | 115605697 | 115618760 | 311683.18 | 110 | 1 | DCLRE1A | 4.68 |
| 228 | chr10 | 115605697 | 115618760 | 311683.18 | 110 | 1 | NHLRC2 | 2.36 |
| 229 | chr6 | 27094219 | 27115646 | 310048.69 | 111 | 1 | HIST1H2BK | 5.69 |
| 230 | chr6 | 27094219 | 27115646 | 310048.69 | 111 | 1 | HIST1H2BJ | 1.11 |
| 231 | chr8 | 94739067 | 94753948 | 306399.79 | 112 | 1 | FAM92A1 | 4.03 |
| 232 | chr8 | 94739067 | 94753948 | 306399.79 | 112 | 1 | TMEM67 | 3.69 |
| 233 | chr8 | 94739067 | 94753948 | 306399.79 | 112 | 1 | RBM12B | 2.52 |
| 234 | chr6 | 32935398 | 32941105 | 304011.89 | 113 | 1 | BRD2 | 5.70 |
| 235 | chr1 | 27018134 | 27024905 | 301918.89 | 114 | 1 | ARID1A | 4.51 |
| 236 | chr19 | 18390487 | 18416372 | 301819.10 | 115 | 1 | LSM4 | 5.32 |
| 237 | chr19 | 18390487 | 18416372 | 301819.10 | 115 | 1 | JUND | 4.84 |
| 238 | chr19 | 18390487 | 18416372 | 301819.10 | 115 | 1 | PGPEP1 | 1.98 |
| 239 | chr10 | 43891049 | 43916828 | 301614.30 | 116 | 1 | HNRNPF | 5.63 |
| 240 | chr10 | 71689055 | 71699999 | 301069.44 | 117 | 1 | na | na |
| 241 | chr12 | 66453989 | 66466300 | 300880.84 | 118 | 1 | na | na |
| 242 | chr6 | 26194444 | 26206840 | 300850.92 | 119 | 1 | na | na |
| 243 | chr8 | 127476965 | 127517947 | 297939.14 | 120 | 1 | na | na |
| 244 | chr8 | 103800188 | 103823262 | 293039.80 | 121 | 1 | na | na |
| 245 | chr15 | 93350938 | 93376211 | 292914.07 | 122 | 1 | na | na |
| 246 | chr6 | 131689098 | 131709803 | 292354.60 | 123 | 1 | na | na |
| 247 | chr9 | 14673331 | 14694063 | 291077.28 | 124 | 1 | ZDHHC21 | 5.46 |
| 248 | chr19 | 59054764 | 59087742 | 290865.96 | 125 | 1 | TRIM28 | 7.20 |
| 249 | chr19 | 59054764 | 59087742 | 290865.96 | 125 | 1 | UBE2M | 4.90 |
| 250 | chr19 | 59054764 | 59087742 | 290865.96 | 125 | 1 | CHMP2A | 4.26 |
| 251 | chr19 | 59054764 | 59087742 | 290865.96 | 125 | 1 | MZF1 | 3.37 |
| 252 | chr19 | 59054764 | 59087742 | 290865.96 | 125 | 1 | ZBTB45 | 2.50 |
| 253 | chr19 | 59054764 | 59087742 | 290865.96 | 125 | 1 | SLC27A5 | 1.08 |
| 254 | chr17 | 44264222 | 44273014 | 290399.76 | 126 | 1 | KANSL1 | 3.20 |
| 255 | chr2 | 219260061 | 219272299 | 290407.74 | 127 | 1 | CTDSP1 | 4.83 |
| 256 | chr14 | 77492135 | 77509116 | 290205.29 | 128 | 1 | IRF2BPL | 3.70 |
| 257 | chr7 | 23507679 | 23516610 | 289632.33 | 129 | 1 | IGF2BP3 | 3.10 |
| 258 | chr1 | 154927986 | 154956785 | 288565.98 | 130 | 1 | SHC1 | 5.99 |
| 259 | chr1 | 154927986 | 154956785 | 288565.98 | 130 | 1 | CKS1B | 4.13 |
| 260 | chr1 | 154927986 | 154956785 | 288565.98 | 130 | 1 | PBXIP1 | 4.07 |
| 261 | chr1 | 154927986 | 154956785 | 288565.98 | 130 | 1 | PMVK | 4.03 |
| 262 | chr1 | 154927986 | 154956785 | 288565.98 | 130 | 1 | PYGO2 | 3.85 |
| 263 | chr1 | 154927986 | 154956785 | 288565.98 | 130 | 1 | FLAD1 | 3.47 |
| 264 | chr13 | 98620140 | 98634525 | 288419.25 | 131 | 1 | na | na |
| 265 | chr9 | 98267491 | 98279215 | 287355.24 | 132 | 1 | PTCH1 | 2.90 |
| 266 | chr12 | 16746523 | 16764836 | 286781.58 | 133 | 1 | LMO3 | 3.36 |
| 267 | chr4 | 175183160 | 175207254 | 285995.78 | 134 | 1 | CEP44 | 4.09 |
| 268 | chr4 | 175183160 | 175207254 | 285995.78 | 134 | 1 | FBXO8 | 2.58 |
| 269 | chr19 | 506207 | 533817 | 284935.20 | 135 | 1 | CDC34 | 5.52 |
| 270 | chr19 | 506207 | 533817 | 284935.20 | 135 | 1 | TPGS1 | 2.97 |
| 271 | chr8 | 32404115 | 32441940 | 284444.00 | 136 | 1 | NRG1 | 2.94 |
| 272 | chr17 | 79817540 | 79830561 | 284248.43 | 137 | 1 | P4HB | 8.33 |
| 273 | chr17 | 79817540 | 79830561 | 284248.43 | 137 | 1 | ARHGDIA | 6.65 |
| 274 | chr17 | 79817540 | 79830561 | 284248.43 | 137 | 1 | ALYREF | 5.76 |
| 275 | chr17 | 79817540 | 79830561 | 284248.43 | 137 | 1 | ANAPC11 | 3.98 |
| 276 | chr17 | 79817540 | 79830561 | 284248.43 | 137 | 1 | FAM195B | 2.78 |
| 277 | chr2 | 232570486 | 232579636 | 280539.00 | 138 | 1 | PTMA | 8.17 |
| 278 | chr1 | 60309208 | 60323031 | 279915.75 | 139 | 0 | HOOK1 | 4.88 |
| 279 | chr8 | 37584803 | 37595617 | 279109.34 | 140 | 0 | ERLIN2 | 4.32 |
| 280 | chr8 | 37584803 | 37595617 | 279109.34 | 140 | 0 | PROSC | 3.54 |
| 281 | chr1 | 16160112 | 16177455 | 279048.87 | 141 | 0 | SPEN | 4.29 |
| 282 | chr1 | 45265122 | 45309200 | 278572.96 | 142 | 0 | RPS8 | 8.59 |
| 283 | chr1 | 45265122 | 45309200 | 278572.96 | 142 | 0 | SNORD46 | 1.82 |
| 284 | chr1 | 45265122 | 45309200 | 278572.96 | 142 | 0 | PTCH2 | 1.74 |
| 285 | chr1 | 45265122 | 45309200 | 278572.96 | 142 | 0 | BTBD19 | 1.37 |
| 286 | chr1 | 45265122 | 45309200 | 278572.96 | 142 | 0 | PLK3 | 1.34 |
| 287 | chr1 | 153918070 | 153964225 | 277853.10 | 143 | 0 | RPS27 | 7.71 |
| 288 | chr1 | 153918070 | 153964225 | 277853.10 | 143 | 0 | SLC39A1 | 6.03 |
| 289 | chr1 | 153918070 | 153964225 | 277853.10 | 143 | 0 | JTB | 5.85 |
| 290 | chr1 | 153918070 | 153964225 | 277853.10 | 143 | 0 | CREB3L4 | 3.79 |
| 291 | chr1 | 153918070 | 153964225 | 277853.10 | 143 | 0 | DENND4B | 3.75 |
| 292 | chr1 | 153918070 | 153964225 | 277853.10 | 143 | 0 | CRTC2 | 3.71 |
| 293 | chr1 | 153918070 | 153964225 | 277853.10 | 143 | 0 | RAB13 | 3.32 |
| 294 | chr1 | 153918070 | 153964225 | 277853.10 | 143 | 0 | GATAD2B | 2.94 |
| 295 | chr2 | 8816417 | 8826599 | 277764.96 | 144 | 0 | ID2 | 4.53 |
| 296 | chr8 | 112663460 | 112676273 | 277657.71 | 145 | 0 | na | na |
| 297 | chr2 | 70311592 | 70316828 | 276879.68 | 146 | 0 | PCBP1 | 6.72 |
| 298 | chr2 | 70311592 | 70316828 | 276879.68 | 146 | 0 | PCBP1-AS1 | 2.86 |
| 299 | chr2 | 30337574 | 30357037 | 274817.56 | 147 | 0 | na | na |
| 300 | chr8 | 129036921 | 129067109 | 274710.80 | 148 | 0 | na | na |
| 301 | chr20 | 30175279 | 30198922 | 273785.94 | 149 | 0 | ID1 | 4.77 |
| 302 | chr19 | 49121586 | 49158192 | 273080.76 | 150 | 0 | RPL18 | 8.33 |
| 303 | chr19 | 49121586 | 49158192 | 273080.76 | 150 | 0 | CA11 | 4.78 |
| 304 | chr19 | 49121586 | 49158192 | 273080.76 | 150 | 0 | DBP | 2.92 |
| 305 | chr19 | 49121586 | 49158192 | 273080.76 | 150 | 0 | SPHK2 | 1.77 |
| 306 | chr1 | 156697854 | 156722654 | 272800.00 | 151 | 0 | HDGF | 5.68 |
| 307 | chr1 | 156697854 | 156722654 | 272800.00 | 151 | 0 | MRPL24 | 4.37 |
| 308 | chr1 | 156697854 | 156722654 | 272800.00 | 151 | 0 | PRCC | 4.25 |
| 309 | chr1 | 156697854 | 156722654 | 272800.00 | 151 | 0 | RRNAD1 | 3.03 |
| 310 | chr1 | 156697854 | 156722654 | 272800.00 | 151 | 0 | ISG20L2 | 2.51 |
| 311 | chr8 | 101961889 | 101966365 | 271961.76 | 152 | 0 | YWHAZ | 5.69 |
| 312 | chr1 | 52303390 | 52317881 | 270546.97 | 153 | 0 | NRD1 | 6.07 |
| 313 | chr11 | 67033928 | 67057902 | 270426.72 | 154 | 0 | ADRBK1 | 4.73 |
| 314 | chr11 | 67033928 | 67057902 | 270426.72 | 154 | 0 | KDM2A | 3.72 |
| 315 | chr11 | 67033928 | 67057902 | 270426.72 | 154 | 0 | ANKRD13D | 3.49 |
| 316 | chr6 | 26020504 | 26033586 | 270404.94 | 155 | 0 | na | na |
| 317 | chr3 | 76853017 | 76877110 | 269600.67 | 156 | 0 | na | na |
| 318 | chr20 | 4225017 | 4232275 | 267457.30 | 157 | 0 | ADRA1D | 6.85 |
| 319 | chr20 | 52195744 | 52241038 | 267234.60 | 158 | 0 | na | na |
| 320 | chr13 | 31584359 | 31598111 | 267063.84 | 159 | 0 | na | na |
| 321 | chr10 | 118890188 | 118898875 | 266604.03 | 160 | 0 | KIAA1598 | 2.84 |
| 322 | chr10 | 118890188 | 118898875 | 266604.03 | 160 | 0 | VAX1 | 1.78 |
| 323 | chr10 | 71718508 | 71737697 | 265767.65 | 161 | 0 | na | na |
| 324 | chr9 | 100744187 | 100748312 | 265237.50 | 162 | 0 | ANP32B | 6.46 |
| 325 | chr19 | 10513232 | 10543867 | 264992.75 | 163 | 0 | CDC37 | 7.05 |
| 326 | chr19 | 10513232 | 10543867 | 264992.75 | 163 | 0 | TYK2 | 4.26 |
| 327 | chr19 | 10513232 | 10543867 | 264992.75 | 163 | 0 | PDE4A | 3.20 |
| 328 | chr19 | 10513232 | 10543867 | 264992.75 | 163 | 0 | MIR1181 | 1.16 |
| 329 | chr6 | 139688414 | 139697888 | 264703.56 | 164 | 0 | CITED2 | 6.98 |
| 330 | chr2 | 70350196 | 70370789 | 263796.33 | 165 | 0 | na | na |
| 331 | chr13 | 100943795 | 100959435 | 263064.80 | 166 | 0 | na | na |
| 332 | chr4 | 15002084 | 15009131 | 261161.82 | 167 | 0 | CPEB2 | 3.38 |
| 333 | chr9 | 118353887 | 118361774 | 260901.96 | 168 | 0 | na | na |
| 334 | chr5 | 87956229 | 87976633 | 260151.00 | 169 | 0 | LINC00461 | 1.12 |
| 335 | chr6 | 157097522 | 157101629 | 259932.03 | 170 | 0 | ARID1B | 3.50 |
| 336 | chr14 | 22108907 | 22127732 | 259785.00 | 171 | 0 | na | na |
| 337 | chr11 | 62413227 | 62448498 | 259947.27 | 172 | 0 | GANAB | 8.39 |
| 338 | chr11 | 62413227 | 62448498 | 259947.27 | 172 | 0 | UBXN1 | 5.55 |
| 339 | chr11 | 62413227 | 62448498 | 259947.27 | 172 | 0 | SNORA57 | 2.93 |
| 340 | chr11 | 62413227 | 62448498 | 259947.27 | 172 | 0 | BSCL2 | 2.15 |
| 341 | chr11 | 62413227 | 62448498 | 259947.27 | 172 | 0 | INTS5 | 2.07 |
| 342 | chr11 | 62413227 | 62448498 | 259947.27 | 172 | 0 | METTL12 | 1.96 |
| 343 | chr6 | 32143142 | 32165142 | 259600.00 | 173 | 0 | PPT2 | 5.20 |
| 344 | chr6 | 32143142 | 32165142 | 259600.00 | 173 | 0 | PBX2 | 5.02 |
| 345 | chr6 | 32143142 | 32165142 | 259600.00 | 173 | 0 | RNF5 | 4.96 |
| 346 | chr6 | 32143142 | 32165142 | 259600.00 | 173 | 0 | AGPAT1 | 3.32 |
| 347 | chr6 | 32143142 | 32165142 | 259600.00 | 173 | 0 | PRRT1 | 2.20 |
| 348 | chr6 | 32143142 | 32165142 | 259600.00 | 173 | 0 | GPSM3 | 1.70 |
| 349 | chr14 | 105863744 | 105888633 | 257850.04 | 174 | 0 | MTA1 | 5.86 |
| 350 | chr14 | 105863744 | 105888633 | 257850.04 | 174 | 0 | TEX22 | 1.32 |
| 351 | chr13 | 101161144 | 101183189 | 257485.60 | 175 | 0 | na | na |
| 352 | chr16 | 29815743 | 29828710 | 257265.28 | 176 | 0 | MAZ | 6.27 |
| 353 | chr16 | 29815743 | 29828710 | 257265.28 | 176 | 0 | KIF22 | 4.57 |
| 354 | chr16 | 29815743 | 29828710 | 257265.28 | 176 | 0 | PRRT2 | 3.48 |
| 355 | chr10 | 74003742 | 74034432 | 257182.20 | 177 | 0 | DDIT4 | 4.82 |
| 356 | chr10 | 74003742 | 74034432 | 257182.20 | 177 | 0 | ANAPC16 | 2.98 |
| 357 | chr10 | 74003742 | 74034432 | 257182.20 | 177 | 0 | ASCC1 | 2.40 |
| 358 | chr11 | 62358304 | 62389831 | 255999.24 | 178 | 0 | B3GAT3 | 4.83 |
| 359 | chr11 | 62358304 | 62389831 | 255999.24 | 178 | 0 | MTA2 | 4.69 |
| 360 | chr11 | 62358304 | 62389831 | 255999.24 | 178 | 0 | EML3 | 4.15 |
| 361 | chr11 | 62358304 | 62389831 | 255999.24 | 178 | 0 | TUT1 | 3.64 |
| 362 | chr11 | 62358304 | 62389831 | 255999.24 | 178 | 0 | ROM1 | 1.07 |
| 363 | chr1 | 17215039 | 17241386 | 254512.02 | 179 | 0 | na | na |
| 364 | chr1 | 6661211 | 6685993 | 254263.32 | 180 | 0 | PHF13 | 4.06 |
| 365 | chr1 | 6661211 | 6685993 | 254263.32 | 180 | 0 | KLHL21 | 3.48 |
| 366 | chr1 | 6661211 | 6685993 | 254263.32 | 180 | 0 | ZBTB48 | 3.20 |
| 367 | chr1 | 6661211 | 6685993 | 254263.32 | 180 | 0 | THAP3 | 2.16 |
| 368 | chr3 | 193851552 | 193860448 | 253447.04 | 181 | 0 | HES1 | 5.05 |
| 369 | chr3 | 197676046 | 197689469 | 252889.32 | 182 | 0 | RPL35A | 7.83 |
| 370 | chr3 | 197676046 | 197689469 | 252889.32 | 182 | 0 | LMLN | 3.45 |
| 371 | chr3 | 197676046 | 197689469 | 252889.32 | 182 | 0 | IQCG | 1.50 |
| 372 | chr1 | 234735063 | 234749621 | 252726.88 | 183 | 0 | IRF2BP2 | 3.31 |
| 373 | chr6 | 26520285 | 26554380 | 252643.95 | 184 | 0 | HMGN4 | 3.94 |
| 374 | chr6 | 26520285 | 26554380 | 252643.95 | 184 | 0 | HCG11 | 2.83 |
| 375 | chr15 | 40571782 | 40584270 | 252132.72 | 185 | 0 | ANKRD63 | 3.15 |
| 376 | chr20 | 49383397 | 49411318 | 251568.21 | 186 | 0 | BCAS4 | 1.65 |
| 377 | chr13 | 30968797 | 30997263 | 251354.78 | 187 | 0 | na | na |
| 378 | chr19 | 49605352 | 49632654 | 250905.38 | 188 | 0 | SNRNP70 | 6.86 |
| 379 | chr19 | 49605352 | 49632654 | 250905.38 | 188 | 0 | TRPM4 | 4.17 |
| 380 | chr19 | 49605352 | 49632654 | 250905.38 | 188 | 0 | PPFIA3 | 3.44 |
| 381 | chr19 | 49605352 | 49632654 | 250905.38 | 188 | 0 | LIN7B | 2.94 |
| 382 | chr16 | 74625468 | 74652555 | 250554.75 | 189 | 0 | GLG1 | 9.20 |
| 383 | chr10 | 115801818 | 115810060 | 248578.72 | 190 | 0 | ADRB1 | 4.92 |
| 384 | chr17 | 56001824 | 56033328 | 248251.52 | 191 | 0 | VEZF1 | 3.46 |
| 385 | chr7 | 115850205 | 115880803 | 248149.78 | 192 | 0 | TES | 3.71 |
| 386 | chr1 | 161123142 | 161172949 | 247540.79 | 193 | 0 | B4GALT3 | 5.12 |
| 387 | chr1 | 161123142 | 161172949 | 247540.79 | 193 | 0 | NDUFS2 | 4.83 |
| 388 | chr1 | 161123142 | 161172949 | 247540.79 | 193 | 0 | UFC1 | 3.82 |
| 389 | chr1 | 161123142 | 161172949 | 247540.79 | 193 | 0 | USP21 | 3.66 |
| 390 | chr1 | 161123142 | 161172949 | 247540.79 | 193 | 0 | PPOX | 3.28 |
| 391 | chr1 | 161123142 | 161172949 | 247540.79 | 193 | 0 | DEDD | 3.04 |
| 392 | chr1 | 161123142 | 161172949 | 247540.79 | 193 | 0 | ADAMTS4 | 2.63 |
| 393 | chr1 | 161123142 | 161172949 | 247540.79 | 193 | 0 | APOA2 | 1.43 |
| 394 | chr1 | 161123142 | 161172949 | 247540.79 | 193 | 0 | TOMM40L | 1.38 |
| 395 | chr17 | 57695130 | 57713391 | 245610.45 | 194 | 0 | CLTC | 5.95 |
| 396 | chr17 | 79868612 | 79895755 | 245101.29 | 195 | 0 | PYCR1 | 6.29 |
| 397 | chr17 | 79868612 | 79895755 | 245101.29 | 195 | 0 | SIRT7 | 3.46 |
| 398 | chr17 | 79868612 | 79895755 | 245101.29 | 195 | 0 | PCYT2 | 2.98 |
| 399 | chr17 | 79868612 | 79895755 | 245101.29 | 195 | 0 | MAFG | 1.27 |
| 400 | chr12 | 14536389 | 14570479 | 244084.40 | 196 | 0 | ATF7IP | 3.21 |
| 401 | chr15 | 69109052 | 69114214 | 244007.74 | 197 | 0 | ANP32A | 4.54 |
| 402 | chr1 | 149855577 | 149872881 | 242948.16 | 198 | 0 | SV2A | 4.73 |
| 403 | chr1 | 149855577 | 149872881 | 242948.16 | 198 | 0 | SF3B4 | 4.64 |
| 404 | chr1 | 149855577 | 149872881 | 242948.16 | 198 | 0 | BOLA1 | 3.47 |
| 405 | chr1 | 149855577 | 149872881 | 242948.16 | 198 | 0 | HIST2H2AA4 | 2.49 |
| 406 | chr1 | 149855577 | 149872881 | 242948.16 | 198 | 0 | HIST2H2BC | 2.16 |
| 407 | chr1 | 149855577 | 149872881 | 242948.16 | 198 | 0 | HIST2H2AA3 | 2.10 |
| 408 | chr1 | 149855577 | 149872881 | 242948.16 | 198 | 0 | HIST2H2BE | 1.89 |
| 409 | chr1 | 149855577 | 149872881 | 242948.16 | 198 | 0 | HIST2H2AC | 1.57 |
| 410 | chr1 | 149855577 | 149872881 | 242948.16 | 198 | 0 | MTMR11 | 1.11 |
| 411 | chr8 | 112444040 | 112458247 | 242513.49 | 199 | 0 | na | na |
| 412 | chr9 | 14716144 | 14734958 | 242136.18 | 200 | 0 | na | na |
| 413 | chr4 | 1856677 | 1874494 | 241064.01 | 201 | 0 | LETM1 | 4.80 |
| 414 | chr4 | 1856677 | 1874494 | 241064.01 | 201 | 0 | WHSC1 | 3.59 |
| 415 | chr11 | 83513015 | 83532078 | 240193.80 | 202 | 0 | na | na |
| 416 | chr4 | 160685204 | 160703294 | 239149.80 | 203 | 0 | na | na |
| 417 | chr19 | 56091287 | 56118329 | 237969.60 | 204 | 0 | ZNF579 | 3.00 |
| 418 | chr19 | 56091287 | 56118329 | 237969.60 | 204 | 0 | FIZ1 | 2.60 |
| 419 | chr19 | 56091287 | 56118329 | 237969.60 | 204 | 0 | ZNF524 | 1.51 |
| 420 | chr16 | 74572239 | 74612034 | 237178.20 | 205 | 0 | na | na |
| 421 | chr12 | 98885005 | 98914836 | 235963.21 | 206 | 0 | TMPO | 4.72 |
| 422 | chr8 | 125738359 | 125768622 | 235748.77 | 207 | 0 | MTSS1 | 2.31 |
| 423 | chr11 | 75045572 | 75063562 | 235848.90 | 208 | 0 | ARRB1 | 3.30 |
| 424 | chr12 | 58227919 | 58241068 | 235104.12 | 209 | 0 | CTDSP2 | 5.44 |
| 425 | chr22 | 23862036 | 23876992 | 234958.76 | 210 | 0 | na | na |
| 426 | chr19 | 54959339 | 54985316 | 234052.77 | 211 | 0 | LENG8 | 6.13 |
| 427 | chr19 | 54959339 | 54985316 | 234052.77 | 211 | 0 | CDC42EP5 | 4.44 |
| 428 | chr6 | 13906161 | 13935724 | 232956.44 | 212 | 0 | RNF182 | 2.74 |
| 429 | chr9 | 89145217 | 89179273 | 232943.04 | 213 | 0 | na | na |
| 430 | chr6 | 24376236 | 24389355 | 232731.06 | 214 | 0 | MRS2 | 3.95 |
| 431 | chr6 | 24376236 | 24389355 | 232731.06 | 214 | 0 | DCDC2 | 3.71 |
| 432 | chr3 | 77062673 | 77091915 | 232181.48 | 215 | 0 | ROBO2 | 5.37 |
| 433 | chr20 | 45980385 | 45990170 | 231904.50 | 216 | 0 | ZMYND8 | 3.77 |
| 434 | chr8 | 119098588 | 119124976 | 231158.88 | 217 | 0 | EXT1 | 4.38 |
| 435 | chr10 | 103873817 | 103894221 | 231177.32 | 218 | 0 | NOLC1 | 6.17 |
| 436 | chr10 | 103873817 | 103894221 | 231177.32 | 218 | 0 | LDB1 | 5.21 |
| 437 | chr10 | 103873817 | 103894221 | 231177.32 | 218 | 0 | PPRC1 | 4.33 |
| 438 | chr6 | 27857491 | 27871300 | 230748.39 | 219 | 0 | HIST1H2BO | 1.27 |
| 439 | chr1 | 36838953 | 36866956 | 230464.69 | 220 | 0 | STK40 | 3.33 |
| 440 | chr1 | 36838953 | 36866956 | 230464.69 | 220 | 0 | LSM10 | 3.22 |
| 441 | chr5 | 140873543 | 140905719 | 229736.64 | 221 | 0 | PCDHGC3 | 5.56 |
| 442 | chr8 | 129131606 | 129146865 | 227816.87 | 222 | 0 | na | na |
| 443 | chr2 | 139567398 | 139585852 | 227353.28 | 223 | 0 | na | na |
| 444 | chr19 | 40926566 | 40951204 | 227162.36 | 224 | 0 | BLVRB | 3.63 |
| 445 | chr19 | 40926566 | 40951204 | 227162.36 | 224 | 0 | SERTAD3 | 2.73 |
| 446 | chr19 | 40926566 | 40951204 | 227162.36 | 224 | 0 | SERTAD1 | 1.72 |
| 447 | chr11 | 16365139 | 16379274 | 226866.75 | 225 | 0 | na | na |
| 448 | chr3 | 197832854 | 197841288 | 226874.60 | 226 | 0 | na | na |
| 449 | chr17 | 73774921 | 73782863 | 226347.00 | 227 | 0 | H3F3B | 6.19 |
| 450 | chr17 | 73774921 | 73782863 | 226347.00 | 227 | 0 | UNK | 4.04 |
| 451 | chr17 | 73774921 | 73782863 | 226347.00 | 227 | 0 | GALK1 | 3.61 |
| 452 | chr4 | 1713099 | 1724456 | 225777.16 | 228 | 0 | TACC3 | 4.81 |
| 453 | chr4 | 1713099 | 1724456 | 225777.16 | 228 | 0 | SLBP | 4.39 |
| 454 | chr4 | 1713099 | 1724456 | 225777.16 | 228 | 0 | TMEM129 | 4.22 |
| 455 | chr8 | 56299461 | 56315661 | 224532.00 | 229 | 0 | na | na |
| 456 | chr1 | 150185847 | 150209750 | 223732.08 | 230 | 0 | ANP32E | 5.23 |
| 457 | chr19 | 58891468 | 58920808 | 223570.80 | 231 | 0 | RPS5 | 9.87 |
| 458 | chr19 | 58891468 | 58920808 | 223570.80 | 231 | 0 | ZNF584 | 2.18 |
| 459 | chr19 | 58891468 | 58920808 | 223570.80 | 231 | 0 | ZNF497 | 2.04 |
| 460 | chr19 | 58891468 | 58920808 | 223570.80 | 231 | 0 | A1BG | 1.98 |
| 461 | chr19 | 58891468 | 58920808 | 223570.80 | 231 | 0 | ZNF132 | 1.34 |
| 462 | chr6 | 151709625 | 151714467 | 223458.30 | 232 | 0 | ZBTB2 | 2.90 |
| 463 | chr20 | 3765912 | 3802225 | 221872.43 | 233 | 0 | CENPB | 4.66 |
| 464 | chr20 | 3765912 | 3802225 | 221872.43 | 233 | 0 | CDC25B | 4.42 |
| 465 | chr20 | 3765912 | 3802225 | 221872.43 | 233 | 0 | C20orf27 | 4.19 |
| 466 | chr20 | 3765912 | 3802225 | 221872.43 | 233 | 0 | MAVS | 3.43 |
| 467 | chr20 | 3765912 | 3802225 | 221872.43 | 233 | 0 | AP5S1 | 2.18 |
| 468 | chr16 | 67183790 | 67226849 | 221323.26 | 234 | 0 | E2F4 | 4.09 |
| 469 | chr16 | 67183790 | 67226849 | 221323.26 | 234 | 0 | HSF4 | 3.88 |
| 470 | chr16 | 67183790 | 67226849 | 221323.26 | 234 | 0 | KIAA0895L | 3.87 |
| 471 | chr16 | 67183790 | 67226849 | 221323.26 | 234 | 0 | FBXL8 | 3.16 |
| 472 | chr16 | 67183790 | 67226849 | 221323.26 | 234 | 0 | TRADD | 2.50 |
| 473 | chr16 | 67183790 | 67226849 | 221323.26 | 234 | 0 | NOL3 | 2.11 |
| 474 | chr16 | 67183790 | 67226849 | 221323.26 | 234 | 0 | B3GNT9 | 1.19 |
| 475 | chr12 | 46118369 | 46125245 | 221063.40 | 235 | 0 | ARID2 | 3.08 |
| 476 | chr9 | 14976433 | 14994726 | 219698.93 | 236 | 0 | na | na |
| 477 | chr20 | 21489962 | 21503523 | 219688.20 | 237 | 0 | NKX2-2 | 3.44 |
| 478 | chr14 | 23769478 | 23792153 | 219040.50 | 238 | 0 | SLC22A17 | 5.61 |
| 479 | chr14 | 23769478 | 23792153 | 219040.50 | 238 | 0 | BCL2L2 | 3.11 |
| 480 | chr14 | 23769478 | 23792153 | 219040.50 | 238 | 0 | BCL2L2-PABPN1 | 1.78 |
| 481 | chr14 | 23769478 | 23792153 | 219040.50 | 238 | 0 | PPP1R3E | 1.22 |
| 482 | chr14 | 23769478 | 23792153 | 219040.50 | 238 | 0 | HOMEZ | 1.03 |
| 483 | chr1 | 154971478 | 154990772 | 218986.90 | 239 | 0 | ADAM15 | 4.24 |
| 484 | chr1 | 154971478 | 154990772 | 218986.90 | 239 | 0 | ZBTB7B | 2.68 |
| 485 | chr6 | 5996190 | 6009823 | 218945.98 | 240 | 0 | NRN1 | 6.52 |
| 486 | chr8 | 127917957 | 127925993 | 218498.84 | 241 | 0 | na | na |
| 487 | chr18 | 3245621 | 3263963 | 218269.80 | 242 | 0 | MYL12B | 5.78 |
| 488 | chr18 | 3245621 | 3263963 | 218269.80 | 242 | 0 | MYL12A | 5.44 |
| 489 | chr11 | 118955170 | 118992829 | 218045.61 | 243 | 0 | HYOU1 | 5.80 |
| 490 | chr11 | 118955170 | 118992829 | 218045.61 | 243 | 0 | DPAGT1 | 5.71 |
| 491 | chr11 | 118955170 | 118992829 | 218045.61 | 243 | 0 | H2AFX | 4.77 |
| 492 | chr11 | 118955170 | 118992829 | 218045.61 | 243 | 0 | HMBS | 3.28 |
| 493 | chr11 | 118955170 | 118992829 | 218045.61 | 243 | 0 | VPS11 | 3.01 |
| 494 | chr11 | 118955170 | 118992829 | 218045.61 | 243 | 0 | C2CD2L | 2.68 |
| 495 | chr11 | 118955170 | 118992829 | 218045.61 | 243 | 0 | HINFP | 1.99 |
| 496 | chr19 | 47612398 | 47617333 | 217929.60 | 244 | 0 | SAE1 | 5.00 |
| 497 | chr19 | 47612398 | 47617333 | 217929.60 | 244 | 0 | ZC3H4 | 3.75 |
| 498 | chr19 | 2041046 | 2062854 | 217643.84 | 245 | 0 | BTBD2 | 4.76 |
| 499 | chr19 | 2041046 | 2062854 | 217643.84 | 245 | 0 | MKNK2 | 3.75 |
| 500 | chr19 | 2041046 | 2062854 | 217643.84 | 245 | 0 | MOB3A | 2.42 |
| 501 | chr19 | 2041046 | 2062854 | 217643.84 | 245 | 0 | IZUMO4 | 1.00 |
| 502 | chr9 | 131445601 | 131466477 | 217527.92 | 246 | 0 | SET | 6.32 |
| 503 | chr9 | 131445601 | 131466477 | 217527.92 | 246 | 0 | WDR34 | 4.91 |
| 504 | chr9 | 131445601 | 131466477 | 217527.92 | 246 | 0 | PKN3 | 3.67 |
| 505 | chr9 | 131445601 | 131466477 | 217527.92 | 246 | 0 | ZDHHC12 | 2.85 |
| 506 | chr19 | 3358862 | 3370505 | 217374.81 | 247 | 0 | NFIC | 3.47 |
| 507 | chr12 | 56109172 | 56140501 | 217423.26 | 248 | 0 | CD63 | 6.79 |
| 508 | chr12 | 56109172 | 56140501 | 217423.26 | 248 | 0 | METTL7B | 4.47 |
| 509 | chr12 | 56109172 | 56140501 | 217423.26 | 248 | 0 | GDF11 | 3.85 |
| 510 | chr12 | 56109172 | 56140501 | 217423.26 | 248 | 0 | ITGA7 | 3.26 |
| 511 | chr10 | 64563719 | 64578483 | 217326.08 | 249 | 0 | EGR2 | 4.47 |
| 512 | chr10 | 64563719 | 64578483 | 217326.08 | 249 | 0 | ADO | 4.09 |
| 513 | chr19 | 13043491 | 13076486 | 217107.10 | 250 | 0 | CALR | 9.12 |
| 514 | chr19 | 13043491 | 13076486 | 217107.10 | 250 | 0 | FARSA | 5.95 |
| 515 | chr19 | 13043491 | 13076486 | 217107.10 | 250 | 0 | RAD23A | 5.73 |
| 516 | chr19 | 13043491 | 13076486 | 217107.10 | 250 | 0 | GADD45GIP1 | 5.63 |
| 517 | chr19 | 13043491 | 13076486 | 217107.10 | 250 | 0 | NFIX | 2.03 |
| 518 | chr12 | 66216041 | 66221947 | 216454.90 | 251 | 0 | HMGA2 | 3.68 |
| 519 | chr1 | 107787257 | 107810461 | 216493.32 | 252 | 0 | na | na |
| 520 | chr8 | 56439226 | 56447812 | 216453.06 | 253 | 0 | na | na |
| 521 | chr14 | 22882633 | 22903527 | 216043.96 | 254 | 0 | na | na |
| 522 | chr19 | 41103465 | 41121151 | 215769.20 | 255 | 0 | LTBP4 | 7.08 |
| 523 | chr19 | 41103465 | 41121151 | 215769.20 | 255 | 0 | SHKBP1 | 4.28 |
| 524 | chr12 | 56497571 | 56522829 | 215450.74 | 256 | 0 | RPL41 | 9.21 |
| 525 | chr12 | 56497571 | 56522829 | 215450.74 | 256 | 0 | ESYT1 | 6.65 |
| 526 | chr12 | 56497571 | 56522829 | 215450.74 | 256 | 0 | PA2G4 | 6.48 |
| 527 | chr12 | 56497571 | 56522829 | 215450.74 | 256 | 0 | MYL6 | 5.45 |
| 528 | chr12 | 56497571 | 56522829 | 215450.74 | 256 | 0 | MYL6B | 4.07 |
| 529 | chr12 | 56497571 | 56522829 | 215450.74 | 256 | 0 | ZC3H10 | 2.07 |
| 530 | chr1 | 17518845 | 17540682 | 214220.97 | 257 | 0 | na | na |
| 531 | chr17 | 61775455 | 61780361 | 213901.60 | 258 | 0 | LIMD2 | 5.73 |
| 532 | chr9 | 132717094 | 132732227 | 212769.98 | 259 | 0 | na | na |
| 533 | chr9 | 14312735 | 14323556 | 212632.65 | 260 | 0 | NFIB | 1.44 |
| 534 | chr17 | 61509300 | 61525757 | 211472.45 | 261 | 0 | CYB561 | 3.58 |
| 535 | chr17 | 61509300 | 61525757 | 211472.45 | 261 | 0 | ACE | 1.49 |
| 536 | chr11 | 64877918 | 64903267 | 210903.68 | 262 | 0 | FAU | 7.36 |
| 537 | chr11 | 64877918 | 64903267 | 210903.68 | 262 | 0 | TM7SF2 | 5.54 |
| 538 | chr11 | 64877918 | 64903267 | 210903.68 | 262 | 0 | SYVN1 | 4.97 |
| 539 | chr11 | 64877918 | 64903267 | 210903.68 | 262 | 0 | VPS51 | 4.93 |
| 540 | chr11 | 64877918 | 64903267 | 210903.68 | 262 | 0 | MRPL49 | 3.79 |
| 541 | chr11 | 64877918 | 64903267 | 210903.68 | 262 | 0 | CDCA5 | 3.77 |
| 542 | chr11 | 64877918 | 64903267 | 210903.68 | 262 | 0 | ZNHIT2 | 3.06 |
| 543 | chr11 | 64877918 | 64903267 | 210903.68 | 262 | 0 | ZFPL1 | 3.03 |
| 544 | chr13 | 98595992 | 98606183 | 210647.97 | 263 | 0 | na | na |
| 545 | chr2 | 134274821 | 134291217 | 210360.68 | 264 | 0 | NCKAP5 | 2.26 |
| 546 | chr6 | 26054099 | 26068251 | 209166.56 | 265 | 0 | HIST1H1C | 3.85 |
| 547 | chr6 | 26054099 | 26068251 | 209166.56 | 265 | 0 | HFE | 2.03 |
| 548 | chr2 | 218866640 | 218883360 | 209167.20 | 266 | 0 | na | na |
| 549 | chr3 | 50358046 | 50397076 | 208810.50 | 267 | 0 | IFRD2 | 5.36 |
| 550 | chr3 | 50358046 | 50397076 | 208810.50 | 267 | 0 | TMEM115 | 5.07 |
| 551 | chr3 | 50358046 | 50397076 | 208810.50 | 267 | 0 | HYAL2 | 4.81 |
| 552 | chr3 | 50358046 | 50397076 | 208810.50 | 267 | 0 | CYB561D2 | 4.29 |
| 553 | chr3 | 50358046 | 50397076 | 208810.50 | 267 | 0 | NPRL2 | 3.84 |
| 554 | chr3 | 50358046 | 50397076 | 208810.50 | 267 | 0 | RASSF1 | 2.83 |
| 555 | chr3 | 50358046 | 50397076 | 208810.50 | 267 | 0 | TUSC2 | 2.76 |
| 556 | chr3 | 50358046 | 50397076 | 208810.50 | 267 | 0 | NAT6 | 2.61 |
| 557 | chr3 | 50358046 | 50397076 | 208810.50 | 267 | 0 | HYAL3 | 1.66 |
| 558 | chr2 | 27578421 | 27604547 | 208485.48 | 268 | 0 | PPM1G | 6.40 |
| 559 | chr2 | 27578421 | 27604547 | 208485.48 | 268 | 0 | SNX17 | 5.22 |
| 560 | chr2 | 27578421 | 27604547 | 208485.48 | 268 | 0 | EIF2B4 | 4.30 |
| 561 | chr2 | 27578421 | 27604547 | 208485.48 | 268 | 0 | GTF3C2 | 4.13 |
| 562 | chr2 | 27578421 | 27604547 | 208485.48 | 268 | 0 | MPV17 | 3.31 |
| 563 | chr2 | 27578421 | 27604547 | 208485.48 | 268 | 0 | ZNF513 | 2.69 |
| 564 | chr11 | 118779901 | 118801077 | 207736.56 | 269 | 0 | na | na |
| 565 | chr12 | 79310305 | 79333561 | 207210.96 | 270 | 0 | na | na |
| 566 | chr19 | 39881118 | 39904611 | 206973.33 | 271 | 0 | RPS16 | 9.93 |
| 567 | chr19 | 39881118 | 39904611 | 206973.33 | 271 | 0 | SUPT5H | 5.41 |
| 568 | chr19 | 39881118 | 39904611 | 206973.33 | 271 | 0 | PAF1 | 4.34 |
| 569 | chr19 | 39881118 | 39904611 | 206973.33 | 271 | 0 | PLEKHG2 | 3.84 |
| 570 | chr19 | 39881118 | 39904611 | 206973.33 | 271 | 0 | MED29 | 3.05 |
| 571 | chr19 | 39881118 | 39904611 | 206973.33 | 271 | 0 | ZFP36 | 1.91 |
| 572 | chr17 | 75522181 | 75525508 | 206673.24 | 272 | 0 | na | na |
| 573 | chr2 | 30369642 | 30387913 | 206645.01 | 273 | 0 | YPEL5 | 5.85 |
| 574 | chr12 | 54068542 | 54073610 | 205811.48 | 274 | 0 | ATP5G2 | 6.92 |
| 575 | chr20 | 49340433 | 49361663 | 205718.70 | 275 | 0 | PARD6B | 2.09 |
| 576 | chr8 | 81656909 | 81687446 | 205514.01 | 276 | 0 | na | na |
| 577 | chr14 | 99729254 | 99741214 | 205353.20 | 277 | 0 | BCL11B | 3.06 |
| 578 | chr3 | 176768757 | 176790597 | 204640.80 | 278 | 0 | na | na |
| 579 | chr1 | 236849605 | 236869372 | 204193.11 | 279 | 0 | ACTN2 | 4.56 |
| 580 | chr17 | 77769808 | 77788304 | 204010.88 | 280 | 0 | CBX2 | 4.59 |
| 581 | chr17 | 77769808 | 77788304 | 204010.88 | 280 | 0 | CBX8 | 3.29 |
| 582 | chr1 | 36614431 | 36627394 | 203648.73 | 281 | 0 | MAP7D1 | 3.66 |
| 583 | chr1 | 36614431 | 36627394 | 203648.73 | 281 | 0 | TRAPPC3 | 2.90 |
| 584 | chr5 | 180669704 | 180689405 | 203314.32 | 282 | 0 | GNB2L1 | 7.72 |
| 585 | chr5 | 180669704 | 180689405 | 203314.32 | 282 | 0 | TRIM41 | 3.29 |
| 586 | chr5 | 180669704 | 180689405 | 203314.32 | 282 | 0 | TRIM52 | 2.38 |
| 587 | chr12 | 54973703 | 54998249 | 203240.88 | 283 | 0 | PPP1R1A | 4.06 |
| 588 | chr12 | 54973703 | 54998249 | 203240.88 | 283 | 0 | PDE1B | 2.46 |
| 589 | chr3 | 49043761 | 49066981 | 202246.20 | 284 | 0 | IMPDH2 | 7.42 |
| 590 | chr3 | 49043761 | 49066981 | 202246.20 | 284 | 0 | WDR6 | 6.18 |
| 591 | chr3 | 49043761 | 49066981 | 202246.20 | 284 | 0 | NDUFAF3 | 5.01 |
| 592 | chr3 | 49043761 | 49066981 | 202246.20 | 284 | 0 | P4HTM | 4.28 |
| 593 | chr3 | 49043761 | 49066981 | 202246.20 | 284 | 0 | DALRD3 | 4.01 |
| 594 | chr6 | 131443886 | 131458398 | 202007.04 | 285 | 0 | AKAP7 | 3.90 |
| 595 | chr2 | 85751433 | 85767863 | 201596.10 | 286 | 0 | MAT2A | 7.71 |
| 596 | chr2 | 85751433 | 85767863 | 201596.10 | 286 | 0 | GGCX | 4.22 |
| 597 | chr6 | 31619633 | 31634477 | 201136.20 | 287 | 0 | PRRC2A | 6.07 |
| 598 | chr6 | 31619633 | 31634477 | 201136.20 | 287 | 0 | BAG6 | 5.96 |
| 599 | chr6 | 31619633 | 31634477 | 201136.20 | 287 | 0 | CSNK2B | 5.37 |
| 600 | chr6 | 31619633 | 31634477 | 201136.20 | 287 | 0 | GPANK1 | 3.03 |
| 601 | chr6 | 31619633 | 31634477 | 201136.20 | 287 | 0 | C6orf47 | 2.43 |
| 602 | chr6 | 31619633 | 31634477 | 201136.20 | 287 | 0 | AIF1 | 1.45 |
| 603 | chr20 | 32250968 | 32274364 | 200737.68 | 288 | 0 | E2F1 | 4.84 |
| 604 | chr20 | 32250968 | 32274364 | 200737.68 | 288 | 0 | NECAB3 | 3.80 |
| 605 | chr20 | 32250968 | 32274364 | 200737.68 | 288 | 0 | PXMP4 | 2.24 |
| 606 | chr6 | 33238779 | 33268175 | 200480.72 | 289 | 0 | RPS18 | 11.21 |
| 607 | chr6 | 33238779 | 33268175 | 200480.72 | 289 | 0 | PFDN6 | 4.98 |
| 608 | chr6 | 33238779 | 33268175 | 200480.72 | 289 | 0 | WDR46 | 4.93 |
| 609 | chr6 | 33238779 | 33268175 | 200480.72 | 289 | 0 | RGL2 | 4.42 |
| 610 | chr6 | 33238779 | 33268175 | 200480.72 | 289 | 0 | VPS52 | 4.11 |
| 611 | chr6 | 33238779 | 33268175 | 200480.72 | 289 | 0 | DAXX | 4.11 |
| 612 | chr6 | 33238779 | 33268175 | 200480.72 | 289 | 0 | TAPBP | 4.04 |
| 613 | chr6 | 33238779 | 33268175 | 200480.72 | 289 | 0 | HCG25 | 3.41 |
| 614 | chr6 | 33238779 | 33268175 | 200480.72 | 289 | 0 | ZBTB22 | 2.16 |
| 615 | chr2 | 68837533 | 68875561 | 200027.28 | 290 | 0 | PROKR1 | 5.30 |
| 616 | chr19 | 49699401 | 49729203 | 199673.40 | 291 | 0 | na | na |
| 617 | chr12 | 111836158 | 111850230 | 199259.52 | 292 | 0 | SH2B3 | 4.24 |
| 618 | chr12 | 111836158 | 111850230 | 199259.52 | 292 | 0 | FAM109A | 1.33 |
| 619 | chr8 | 77585804 | 77598051 | 199136.22 | 293 | 0 | ZFHX4 | 3.23 |
| 620 | chr6 | 42748665 | 42752252 | 198719.80 | 294 | 0 | TBCC | 3.60 |
| 621 | chr14 | 103051477 | 103061215 | 198655.20 | 295 | 0 | RCOR1 | 5.01 |
| 622 | chr19 | 12831494 | 12848790 | 198558.08 | 296 | 0 | C19orf43 | 6.51 |
| 623 | chr19 | 12831494 | 12848790 | 198558.08 | 296 | 0 | TNPO2 | 5.48 |
| 624 | chr19 | 12831494 | 12848790 | 198558.08 | 296 | 0 | DHPS | 5.16 |
| 625 | chr19 | 12831494 | 12848790 | 198558.08 | 296 | 0 | ASNA1 | 4.76 |
| 626 | chr19 | 12831494 | 12848790 | 198558.08 | 296 | 0 | FBXW9 | 2.92 |
| 627 | chr12 | 19776685 | 19796688 | 198429.76 | 297 | 0 | na | na |
| 628 | chr14 | 100841103 | 100859513 | 198275.70 | 298 | 0 | WARS | 6.13 |
| 629 | chr14 | 100841103 | 100859513 | 198275.70 | 298 | 0 | WDR25 | 1.37 |
| 630 | chr1 | 156073377 | 156096528 | 197941.05 | 299 | 0 | MEX3A | 4.01 |
| 631 | chr1 | 156073377 | 156096528 | 197941.05 | 299 | 0 | LMNA | 3.86 |
| 632 | chr10 | 115742104 | 115750813 | 197345.94 | 300 | 0 | na | na |
| 633 | chr20 | 61405452 | 61437382 | 197327.40 | 301 | 0 | OGFR | 3.47 |
| 634 | chr17 | 43221140 | 43250756 | 197242.56 | 302 | 0 | HEXIM1 | 3.30 |
| 635 | chr17 | 43221140 | 43250756 | 197242.56 | 302 | 0 | ACBD4 | 1.51 |
| 636 | chr17 | 43221140 | 43250756 | 197242.56 | 302 | 0 | HEXIM2 | 1.28 |
| 637 | chr17 | 43221140 | 43250756 | 197242.56 | 302 | 0 | PLCD3 | 1.27 |
| 638 | chr14 | 77412914 | 77428987 | 196894.25 | 303 | 0 | na | na |
| 639 | chr6 | 159420683 | 159440717 | 196533.54 | 304 | 0 | TAGAP | 3.15 |
| 640 | chr6 | 159420683 | 159440717 | 196533.54 | 304 | 0 | RSPH3 | 1.26 |
| 641 | chr1 | 8936866 | 8950486 | 196400.40 | 305 | 0 | ENO1 | 8.49 |
| 642 | chr8 | 12603900 | 12615712 | 196079.20 | 306 | 0 | LONRF1 | 3.54 |
| 643 | chr19 | 50073721 | 50095874 | 194503.34 | 307 | 0 | NOSIP | 5.61 |
| 644 | chr19 | 50073721 | 50095874 | 194503.34 | 307 | 0 | PRR12 | 3.55 |
| 645 | chr3 | 176912600 | 176917219 | 193905.62 | 308 | 0 | TBL1XR1 | 3.59 |
| 646 | chr20 | 1929359 | 1946420 | 193812.96 | 309 | 0 | na | na |
| 647 | chr9 | 89254399 | 89267858 | 193405.83 | 310 | 0 | na | na |
| 648 | chr10 | 26026918 | 26036793 | 193155.00 | 311 | 0 | na | na |
| 649 | chr1 | 226295405 | 226315900 | 192448.05 | 312 | 0 | na | na |
| 650 | chr1 | 162357165 | 162369164 | 192223.98 | 313 | 0 | NOS1AP | 1.46 |
| 651 | chrX | 39955752 | 39969394 | 191942.94 | 314 | 0 | na | na |
| 652 | chr16 | 30064030 | 30088155 | 191793.75 | 315 | 0 | ALDOA | 6.44 |
| 653 | chr16 | 30064030 | 30088155 | 191793.75 | 315 | 0 | PPP4C | 4.48 |
| 654 | chr16 | 30064030 | 30088155 | 191793.75 | 315 | 0 | YPEL3 | 1.79 |
| 655 | chr15 | 96873219 | 96884987 | 190406.24 | 316 | 0 | na | na |
| 656 | chr8 | 125548116 | 125553417 | 190305.90 | 317 | 0 | NDUFB9 | 9.43 |
| 657 | chr8 | 125548116 | 125553417 | 190305.90 | 317 | 0 | TATDN1 | 4.68 |
| 658 | chr3 | 47821351 | 47845877 | 190321.76 | 318 | 0 | SMARCC1 | 5.66 |
| 659 | chr3 | 47821351 | 47845877 | 190321.76 | 318 | 0 | DHX30 | 5.06 |
| 660 | chr8 | 6109342 | 6123495 | 189508.67 | 319 | 0 | na | na |
| 661 | chr17 | 73256751 | 73268303 | 189106.24 | 320 | 0 | MRPS7 | 5.49 |
| 662 | chr17 | 73256751 | 73268303 | 189106.24 | 320 | 0 | GGA3 | 4.32 |
| 663 | chr17 | 73256751 | 73268303 | 189106.24 | 320 | 0 | SLC25A19 | 2.88 |
| 664 | chr17 | 73256751 | 73268303 | 189106.24 | 320 | 0 | MIF4GD | 2.39 |
| 665 | chr20 | 19192142 | 19211302 | 188726.00 | 321 | 0 | SLC24A3 | 4.37 |
| 666 | chr8 | 101732329 | 101735703 | 188100.50 | 322 | 0 | PABPC1 | 9.43 |
| 667 | chr2 | 242625213 | 242642773 | 187540.80 | 323 | 0 | DTYMK | 4.20 |
| 668 | chr2 | 242625213 | 242642773 | 187540.80 | 323 | 0 | ING5 | 3.33 |
| 669 | chr2 | 242625213 | 242642773 | 187540.80 | 323 | 0 | D2HGDH | 3.26 |
| 670 | chr15 | 50645016 | 50649037 | 187418.81 | 324 | 0 | GABPB1 | 2.16 |
| 671 | chr17 | 46079096 | 46102121 | 187423.50 | 325 | 0 | CDK5RAP3 | 6.76 |
| 672 | chr6 | 140190168 | 140206160 | 187106.40 | 326 | 0 | na | na |
| 673 | chr18 | 56657573 | 56680846 | 186649.46 | 327 | 0 | na | na |
| 674 | chr1 | 157950313 | 157973388 | 185984.50 | 328 | 0 | KIRREL | 5.43 |
| 675 | chr16 | 2254727 | 2274468 | 185960.22 | 329 | 0 | MLST8 | 4.21 |
| 676 | chr16 | 2254727 | 2274468 | 185960.22 | 329 | 0 | ECI1 | 3.73 |
| 677 | chr16 | 2254727 | 2274468 | 185960.22 | 329 | 0 | E4F1 | 3.65 |
| 678 | chr16 | 2254727 | 2274468 | 185960.22 | 329 | 0 | PGP | 2.33 |
| 679 | chr16 | 2254727 | 2274468 | 185960.22 | 329 | 0 | CASKIN1 | 2.01 |
| 680 | chr17 | 61903626 | 61921030 | 185526.64 | 330 | 0 | PSMC5 | 5.96 |
| 681 | chr17 | 61903626 | 61921030 | 185526.64 | 330 | 0 | FTSJ3 | 5.94 |
| 682 | chr17 | 61903626 | 61921030 | 185526.64 | 330 | 0 | SMARCD2 | 4.67 |
| 683 | chr9 | 16704080 | 16728208 | 185061.76 | 331 | 0 | na | na |
| 684 | chr1 | 155213116 | 155247829 | 184673.16 | 332 | 0 | SCAMP3 | 5.84 |
| 685 | chr1 | 155213116 | 155247829 | 184673.16 | 332 | 0 | CLK2 | 5.30 |
| 686 | chr1 | 155213116 | 155247829 | 184673.16 | 332 | 0 | FDPS | 4.96 |
| 687 | chr1 | 155213116 | 155247829 | 184673.16 | 332 | 0 | FAM189B | 4.54 |
| 688 | chr1 | 155213116 | 155247829 | 184673.16 | 332 | 0 | GBA | 3.97 |
| 689 | chr1 | 155213116 | 155247829 | 184673.16 | 332 | 0 | HCN3 | 2.11 |
| 690 | chr16 | 30660782 | 30672453 | 184635.22 | 333 | 0 | SRCAP | 3.80 |
| 691 | chr16 | 30660782 | 30672453 | 184635.22 | 333 | 0 | PRR14 | 3.28 |
| 692 | chr7 | 122605105 | 122613311 | 184306.76 | 334 | 0 | na | na |
| 693 | chr1 | 8657575 | 8668915 | 183934.80 | 335 | 0 | na | na |
| 694 | chr17 | 73025943 | 73044043 | 183896.00 | 336 | 0 | ATP5H | 6.79 |
| 695 | chr17 | 73025943 | 73044043 | 183896.00 | 336 | 0 | ICT1 | 4.26 |
| 696 | chr17 | 73025943 | 73044043 | 183896.00 | 336 | 0 | KCTD2 | 3.23 |
| 697 | chr17 | 73025943 | 73044043 | 183896.00 | 336 | 0 | SLC16A5 | 2.75 |
| 698 | chr9 | 123690942 | 123715970 | 183705.52 | 337 | 0 | na | na |
| 699 | chr18 | 56529477 | 56539535 | 182955.02 | 338 | 0 | ZNF532 | 4.08 |
| 700 | chr1 | 61541963 | 61550286 | 182856.31 | 339 | 0 | NFIA | 1.50 |
| 701 | chr7 | 83764416 | 83787150 | 182099.34 | 340 | 0 | SEMA3A | 2.50 |
| 702 | chr9 | 91924514 | 91935404 | 181645.20 | 341 | 0 | CKS2 | 4.87 |
| 703 | chr9 | 91924514 | 91935404 | 181645.20 | 341 | 0 | SECISBP2 | 2.96 |
| 704 | chr2 | 178127463 | 178131754 | 181552.21 | 342 | 0 | NFE2L2 | 3.30 |
| 705 | chr9 | 89559635 | 89564081 | 181352.34 | 343 | 0 | GAS1 | 6.35 |
| 706 | chr17 | 7464567 | 7488127 | 181412.00 | 344 | 0 | EIF4A1 | 7.49 |
| 707 | chr17 | 7464567 | 7488127 | 181412.00 | 344 | 0 | MPDU1 | 4.85 |
| 708 | chr17 | 7464567 | 7488127 | 181412.00 | 344 | 0 | SENP3 | 3.61 |
| 709 | chr17 | 7464567 | 7488127 | 181412.00 | 344 | 0 | FXR2 | 3.48 |
| 710 | chr17 | 7464567 | 7488127 | 181412.00 | 344 | 0 | SNORD10 | 2.63 |
| 711 | chr17 | 7464567 | 7488127 | 181412.00 | 344 | 0 | SNORA48 | 2.17 |
| 712 | chr17 | 7464567 | 7488127 | 181412.00 | 344 | 0 | CD68 | 1.63 |
| 713 | chr3 | 47553842 | 47584462 | 180964.20 | 345 | 0 | na | na |
| 714 | chr8 | 95906308 | 95918308 | 180720.00 | 346 | 0 | CCNE2 | 2.67 |
| 715 | chr15 | 66992675 | 66999854 | 179690.37 | 347 | 0 | SMAD6 | 2.65 |
| 716 | chr12 | 7023570 | 7053947 | 179528.07 | 348 | 0 | PHB2 | 6.45 |
| 717 | chr12 | 7023570 | 7053947 | 179528.07 | 348 | 0 | ATN1 | 4.83 |
| 718 | chr12 | 7023570 | 7053947 | 179528.07 | 348 | 0 | C12orf57 | 4.83 |
| 719 | chr12 | 7023570 | 7053947 | 179528.07 | 348 | 0 | SCARNA12 | 3.57 |
| 720 | chr12 | 7023570 | 7053947 | 179528.07 | 348 | 0 | ENO2 | 3.56 |
| 721 | chr12 | 7023570 | 7053947 | 179528.07 | 348 | 0 | EMG1 | 2.93 |
| 722 | chr12 | 42313388 | 42335695 | 179348.28 | 349 | 0 | na | na |
| 723 | chr17 | 48784071 | 48798184 | 178952.84 | 350 | 0 | LUC7L3 | 5.82 |
| 724 | chr17 | 48784071 | 48798184 | 178952.84 | 350 | 0 | ANKRD40 | 3.57 |
| 725 | chr1 | 241681835 | 241697565 | 178692.80 | 351 | 0 | FH | 6.11 |
| 726 | chr1 | 241681835 | 241697565 | 178692.80 | 351 | 0 | KMO | 4.14 |
| 727 | chr8 | 126439261 | 126444488 | 178606.59 | 352 | 0 | TRIB1 | 3.32 |
| 728 | chr2 | 143298028 | 143315909 | 178452.38 | 353 | 0 | na | na |
| 729 | chr22 | 29662120 | 29665531 | 178224.75 | 354 | 0 | na | na |
| 730 | chr17 | 75224206 | 75243754 | 178277.76 | 355 | 0 | 9-Sep | 4.67 |
| 731 | chr16 | 2509483 | 2525654 | 178204.42 | 356 | 0 | C16orf59 | 3.32 |
| 732 | chr16 | 2509483 | 2525654 | 178204.42 | 356 | 0 | CCNF | 3.22 |
| 733 | chr16 | 2509483 | 2525654 | 178204.42 | 356 | 0 | TBC1D24 | 2.51 |
| 734 | chr16 | 2509483 | 2525654 | 178204.42 | 356 | 0 | NTN3 | 2.44 |
| 735 | chr8 | 145114247 | 145160001 | 177983.06 | 357 | 0 | CYC1 | 6.68 |
| 736 | chr8 | 145114247 | 145160001 | 177983.06 | 357 | 0 | GPAA1 | 5.62 |
| 737 | chr8 | 145114247 | 145160001 | 177983.06 | 357 | 0 | OPLAH | 4.91 |
| 738 | chr8 | 145114247 | 145160001 | 177983.06 | 357 | 0 | MAF1 | 3.94 |
| 739 | chr8 | 145114247 | 145160001 | 177983.06 | 357 | 0 | EXOSC4 | 3.34 |
| 740 | chr8 | 145114247 | 145160001 | 177983.06 | 357 | 0 | SHARPIN | 3.28 |
| 741 | chr2 | 27433791 | 27441613 | 177559.40 | 358 | 0 | SLC5A6 | 6.64 |
| 742 | chr2 | 27433791 | 27441613 | 177559.40 | 358 | 0 | CAD | 6.19 |
| 743 | chr6 | 30684012 | 30690165 | 177329.46 | 359 | 0 | TUBB | 8.82 |
| 744 | chr6 | 30684012 | 30690165 | 177329.46 | 359 | 0 | FLOT1 | 4.56 |
| 745 | chr6 | 30684012 | 30690165 | 177329.46 | 359 | 0 | NRM | 4.44 |
| 746 | chr6 | 30684012 | 30690165 | 177329.46 | 359 | 0 | MDC1 | 3.84 |
| 747 | chr6 | 30684012 | 30690165 | 177329.46 | 359 | 0 | DHX16 | 3.70 |
| 748 | chr6 | 30684012 | 30690165 | 177329.46 | 359 | 0 | IER3 | 2.31 |
| 749 | chr6 | 30684012 | 30690165 | 177329.46 | 359 | 0 | PPP1R18 | 2.24 |
| 750 | chr9 | 118623268 | 118630064 | 177171.72 | 360 | 0 | na | na |
| 751 | chr7 | 121939224 | 121947476 | 176510.28 | 361 | 0 | FEZF1-AS1 | 4.55 |
| 752 | chr7 | 121939224 | 121947476 | 176510.28 | 361 | 0 | FEZF1 | 3.76 |
| 753 | chr20 | 17945550 | 17951223 | 176316.84 | 362 | 0 | SNX5 | 4.04 |
| 754 | chr14 | 23445241 | 23479957 | 176357.28 | 363 | 0 | PSMB5 | 5.91 |
| 755 | chr14 | 23445241 | 23479957 | 176357.28 | 363 | 0 | HAUS4 | 4.09 |
| 756 | chr14 | 23445241 | 23479957 | 176357.28 | 363 | 0 | AJUBA | 2.60 |
| 757 | chr14 | 23445241 | 23479957 | 176357.28 | 363 | 0 | C14orf93 | 1.71 |
| 758 | chr20 | 30134716 | 30162348 | 176292.16 | 364 | 0 | HM13 | 5.73 |
| 759 | chr20 | 30134716 | 30162348 | 176292.16 | 364 | 0 | HM13-AS1 | 1.12 |
| 760 | chr4 | 108075113 | 108094521 | 175642.40 | 365 | 0 | na | na |
| 761 | chr6 | 141954580 | 141965019 | 175479.59 | 366 | 0 | na | na |
| 762 | chr12 | 122237607 | 122243103 | 175377.36 | 367 | 0 | SETD1B | 2.10 |
| 763 | chr8 | 42391905 | 42411109 | 175332.52 | 368 | 0 | SLC20A2 | 3.89 |
| 764 | chr19 | 50369588 | 50381453 | 174771.45 | 369 | 0 | PTOV1 | 5.73 |
| 765 | chr19 | 50369588 | 50381453 | 174771.45 | 369 | 0 | PNKP | 4.41 |
| 766 | chr19 | 50369588 | 50381453 | 174771.45 | 369 | 0 | TBC1D17 | 3.67 |
| 767 | chr19 | 50369588 | 50381453 | 174771.45 | 369 | 0 | AKT1S1 | 3.31 |
| 768 | chr8 | 21404323 | 21411861 | 174730.84 | 370 | 0 | na | na |
| 769 | chr15 | 93443099 | 93461406 | 174099.57 | 371 | 0 | CHD2 | 3.21 |
| 770 | chr1 | 151019822 | 151043897 | 174062.25 | 372 | 0 | MLLT11 | 3.75 |
| 771 | chr1 | 151019822 | 151043897 | 174062.25 | 372 | 0 | CDC42SE1 | 3.50 |
| 772 | chr1 | 151019822 | 151043897 | 174062.25 | 372 | 0 | C1orf56 | 2.70 |
| 773 | chr1 | 151019822 | 151043897 | 174062.25 | 372 | 0 | GABPB2 | 1.49 |
| 774 | chr19 | 51222006 | 51244675 | 173871.23 | 373 | 0 | CLEC11A | 6.96 |
| 775 | chr19 | 51222006 | 51244675 | 173871.23 | 373 | 0 | SHANK1 | 2.30 |
| 776 | chr11 | 64007675 | 64019821 | 173687.80 | 374 | 0 | FKBP2 | 4.91 |
| 777 | chr11 | 64007675 | 64019821 | 173687.80 | 374 | 0 | PPP1R14B | 4.61 |
| 778 | chr11 | 64007675 | 64019821 | 173687.80 | 374 | 0 | VEGFB | 4.27 |
| 779 | chr11 | 64007675 | 64019821 | 173687.80 | 374 | 0 | PLCB3 | 3.73 |
| 780 | chr11 | 64007675 | 64019821 | 173687.80 | 374 | 0 | TRPT1 | 3.73 |
| 781 | chr11 | 64007675 | 64019821 | 173687.80 | 374 | 0 | DNAJC4 | 3.13 |
| 782 | chr11 | 64007675 | 64019821 | 173687.80 | 374 | 0 | NUDT22 | 2.70 |
| 783 | chr11 | 64007675 | 64019821 | 173687.80 | 374 | 0 | GPR137 | 2.60 |
| 784 | chr11 | 64007675 | 64019821 | 173687.80 | 374 | 0 | BAD | 2.49 |
| 785 | chr8 | 32065258 | 32085227 | 173530.61 | 375 | 0 | na | na |
| 786 | chr9 | 137288416 | 137306261 | 173274.95 | 376 | 0 | na | na |
| 787 | chr22 | 24541515 | 24557956 | 172959.32 | 377 | 0 | na | na |
| 788 | chrX | 40030055 | 40036645 | 171537.70 | 378 | 0 | BCOR | 4.36 |
| 789 | chr11 | 66078805 | 66086777 | 171318.28 | 379 | 0 | CD248 | 7.71 |
| 790 | chr11 | 66078805 | 66086777 | 171318.28 | 379 | 0 | B3GNT1 | 6.15 |
| 791 | chr11 | 66078805 | 66086777 | 171318.28 | 379 | 0 | RAB1B | 5.21 |
| 792 | chr11 | 66078805 | 66086777 | 171318.28 | 379 | 0 | BRMS1 | 3.62 |
| 793 | chr11 | 66078805 | 66086777 | 171318.28 | 379 | 0 | CNIH2 | 1.23 |
| 794 | chr2 | 183771635 | 183785975 | 171076.20 | 380 | 0 | na | na |
| 795 | chr2 | 43446468 | 43455094 | 171053.58 | 381 | 0 | ZFP36L2 | 2.52 |
| 796 | chr16 | 30405527 | 30441506 | 170900.25 | 382 | 0 | DCTPP1 | 3.96 |
| 797 | chr16 | 30405527 | 30441506 | 170900.25 | 382 | 0 | SEPHS2 | 3.70 |
| 798 | chr16 | 30405527 | 30441506 | 170900.25 | 382 | 0 | TBC1D10B | 3.47 |
| 799 | chr16 | 30405527 | 30441506 | 170900.25 | 382 | 0 | ZNF48 | 2.27 |
| 800 | chr16 | 30405527 | 30441506 | 170900.25 | 382 | 0 | ZNF771 | 1.27 |
| 801 | chr16 | 65152871 | 65160032 | 171004.68 | 383 | 0 | CDH11 | 5.60 |
| 802 | chr6 | 70571699 | 70578541 | 170981.58 | 384 | 0 | COL19A1 | 2.71 |
| 803 | chr3 | 34302579 | 34319551 | 170568.60 | 385 | 0 | na | na |
| 804 | chr2 | 145073192 | 145090969 | 170481.43 | 386 | 0 | na | na |
| 805 | chr16 | 30568740 | 30598037 | 170215.57 | 387 | 0 | ZNF785 | 2.48 |
| 806 | chr16 | 30568740 | 30598037 | 170215.57 | 387 | 0 | ZNF764 | 2.29 |
| 807 | chr16 | 30568740 | 30598037 | 170215.57 | 387 | 0 | ZNF689 | 2.02 |
| 808 | chr16 | 30568740 | 30598037 | 170215.57 | 387 | 0 | ZNF688 | 1.69 |
| 809 | chr8 | 125382470 | 125385874 | 170268.08 | 388 | 0 | TMEM65 | 3.25 |
| 810 | chr9 | 127623137 | 127633273 | 170183.44 | 389 | 0 | RPL35 | 7.17 |
| 811 | chr9 | 127623137 | 127633273 | 170183.44 | 389 | 0 | ARPC5L | 2.58 |
| 812 | chr18 | 3446744 | 3452958 | 169704.34 | 390 | 0 | TGIF1 | 1.70 |
| 813 | chr3 | 136467592 | 136472942 | 169488.00 | 391 | 0 | STAG1 | 2.48 |
| 814 | chr8 | 121455877 | 121459010 | 169369.98 | 392 | 0 | na | na |
| 815 | chr7 | 150054149 | 150077149 | 168820.00 | 393 | 0 | RARRES2 | 6.18 |
| 816 | chr7 | 150054149 | 150077149 | 168820.00 | 393 | 0 | REPIN1 | 4.20 |
| 817 | chr7 | 150054149 | 150077149 | 168820.00 | 393 | 0 | LRRC61 | 3.44 |
| 818 | chr7 | 150054149 | 150077149 | 168820.00 | 393 | 0 | ZNF775 | 1.12 |
| 819 | chr17 | 62492058 | 62503874 | 168850.64 | 394 | 0 | DDX5 | 7.58 |
| 820 | chr17 | 62492058 | 62503874 | 168850.64 | 394 | 0 | CEP95 | 4.14 |
| 821 | chr17 | 62492058 | 62503874 | 168850.64 | 394 | 0 | POLG2 | 3.99 |
| 822 | chr7 | 99678436 | 99699903 | 168086.61 | 395 | 0 | MCM7 | 6.56 |
| 823 | chr7 | 99678436 | 99699903 | 168086.61 | 395 | 0 | COPS6 | 5.38 |
| 824 | chr7 | 99678436 | 99699903 | 168086.61 | 395 | 0 | AP4M1 | 3.26 |
| 825 | chr7 | 99678436 | 99699903 | 168086.61 | 395 | 0 | TAF6 | 3.25 |
| 826 | chr7 | 99678436 | 99699903 | 168086.61 | 395 | 0 | CNPY4 | 3.01 |
| 827 | chr7 | 99678436 | 99699903 | 168086.61 | 395 | 0 | ZNF3 | 3.01 |
| 828 | chr7 | 99678436 | 99699903 | 168086.61 | 395 | 0 | ZSCAN21 | 1.73 |
| 829 | chr15 | 99403361 | 99418370 | 167950.71 | 396 | 0 | na | na |
| 830 | chr6 | 16946006 | 16964792 | 167758.98 | 397 | 0 | na | na |
| 831 | chr7 | 26238686 | 26242898 | 167637.60 | 398 | 0 | HNRNPA2B1 | 7.54 |
| 832 | chr7 | 26238686 | 26242898 | 167637.60 | 398 | 0 | CBX3 | 4.46 |
| 833 | chr7 | 26238686 | 26242898 | 167637.60 | 398 | 0 | NFE2L3 | 1.64 |
| 834 | chr2 | 183896177 | 183904152 | 167554.75 | 399 | 0 | NCKAP1 | 5.44 |
| 835 | chr8 | 145732946 | 145754596 | 167138.00 | 400 | 0 | RECQL4 | 4.45 |
| 836 | chr8 | 145732946 | 145754596 | 167138.00 | 400 | 0 | MFSD3 | 3.22 |
| 837 | chr8 | 145732946 | 145754596 | 167138.00 | 400 | 0 | LRRC14 | 1.99 |
| 838 | chr8 | 145732946 | 145754596 | 167138.00 | 400 | 0 | C8orf82 | 1.97 |
| 839 | chr10 | 74078840 | 74093758 | 167081.60 | 401 | 0 | DNAJB12 | 3.88 |
| 840 | chr1 | 17556865 | 17565834 | 166733.71 | 402 | 0 | na | na |
| 841 | chr6 | 35686327 | 35700953 | 166590.14 | 403 | 0 | FKBP5 | 1.72 |
| 842 | chr1 | 155162897 | 155179924 | 166524.06 | 404 | 0 | MTX1 | 3.67 |
| 843 | chr1 | 155162897 | 155179924 | 166524.06 | 404 | 0 | THBS3 | 3.64 |
| 844 | chr1 | 155162897 | 155179924 | 166524.06 | 404 | 0 | GBAP1 | 2.65 |
| 845 | chr1 | 155162897 | 155179924 | 166524.06 | 404 | 0 | TRIM46 | 1.75 |
| 846 | chr1 | 155162897 | 155179924 | 166524.06 | 404 | 0 | MUC1 | 1.26 |
| 847 | chr8 | 96280198 | 96283670 | 166308.80 | 405 | 0 | C8orf37 | 1.59 |
| 848 | chr10 | 104158883 | 104195996 | 165895.11 | 406 | 0 | CUEDC2 | 5.15 |
| 849 | chr10 | 104158883 | 104195996 | 165895.11 | 406 | 0 | FBXL15 | 3.44 |
| 850 | chr10 | 104158883 | 104195996 | 165895.11 | 406 | 0 | NFKB2 | 2.62 |
| 851 | chr10 | 104158883 | 104195996 | 165895.11 | 406 | 0 | C10orf95 | 2.40 |
| 852 | chr10 | 104158883 | 104195996 | 165895.11 | 406 | 0 | TMEM180 | 2.25 |
| 853 | chr10 | 104158883 | 104195996 | 165895.11 | 406 | 0 | PSD | 2.18 |
| 854 | chr2 | 174827516 | 174831202 | 165833.14 | 407 | 0 | SP3 | 2.70 |
| 855 | chr13 | 111357822 | 111368962 | 165651.80 | 408 | 0 | CARS2 | 3.32 |
| 856 | chr13 | 111357822 | 111368962 | 165651.80 | 408 | 0 | ING1 | 1.79 |
| 857 | chr1 | 107682513 | 107690087 | 165188.94 | 409 | 0 | NTNG1 | 4.04 |
| 858 | chr18 | 46353143 | 46378379 | 165295.80 | 410 | 0 | na | na |
| 859 | chr17 | 65353931 | 65375096 | 164875.35 | 411 | 0 | PSMD12 | 3.57 |
| 860 | chr17 | 65353931 | 65375096 | 164875.35 | 411 | 0 | PITPNC1 | 1.54 |
| 861 | chr1 | 199119570 | 199133398 | 164968.04 | 412 | 0 | na | na |
| 862 | chr6 | 33167349 | 33176939 | 164660.30 | 413 | 0 | SLC39A7 | 6.53 |
| 863 | chr6 | 33167349 | 33176939 | 164660.30 | 413 | 0 | RING1 | 4.94 |
| 864 | chr6 | 33167349 | 33176939 | 164660.30 | 413 | 0 | RXRB | 4.81 |
| 865 | chr6 | 33167349 | 33176939 | 164660.30 | 413 | 0 | HSD17B8 | 3.84 |
| 866 | chr2 | 200319917 | 200328488 | 163877.52 | 414 | 0 | SATB2 | 2.11 |
| 867 | chr19 | 1406738 | 1416656 | 163349.46 | 415 | 0 | RPS15 | 9.35 |
| 868 | chr19 | 1406738 | 1416656 | 163349.46 | 415 | 0 | DAZAP1 | 5.51 |
| 869 | chr19 | 1406738 | 1416656 | 163349.46 | 415 | 0 | NDUFS7 | 3.88 |
| 870 | chr19 | 1406738 | 1416656 | 163349.46 | 415 | 0 | GAMT | 3.61 |
| 871 | chr9 | 16615855 | 16629017 | 163208.80 | 416 | 0 | na | na |
| 872 | chr19 | 566798 | 577394 | 162966.48 | 417 | 0 | BSG | 9.16 |
| 873 | chr19 | 566798 | 577394 | 162966.48 | 417 | 0 | HCN2 | 3.52 |
| 874 | chr11 | 83386494 | 83406310 | 162689.36 | 418 | 0 | DLG2 | 3.02 |
| 875 | chr19 | 1590596 | 1606552 | 162591.64 | 419 | 0 | MBD3 | 5.22 |
| 876 | chr19 | 1590596 | 1606552 | 162591.64 | 419 | 0 | UQCR11 | 4.38 |
| 877 | chr19 | 1590596 | 1606552 | 162591.64 | 419 | 0 | MEX3D | 2.08 |
| 878 | chr12 | 57022942 | 57040703 | 162335.54 | 420 | 0 | ATP5B | 8.24 |
| 879 | chr12 | 57022942 | 57040703 | 162335.54 | 420 | 0 | BAZ2A | 4.17 |
| 880 | chr20 | 20714784 | 20725378 | 162300.08 | 421 | 0 | RALGAPA2 | 2.23 |
| 881 | chr1 | 241714269 | 241722312 | 162227.31 | 422 | 0 | na | na |
| 882 | chr16 | 57878982 | 57897172 | 162072.90 | 423 | 0 | na | na |
| 883 | chr19 | 7967414 | 7991218 | 162105.24 | 424 | 0 | TIMM44 | 5.66 |
| 884 | chr19 | 7967414 | 7991218 | 162105.24 | 424 | 0 | MAP2K7 | 3.96 |
| 885 | chr19 | 7967414 | 7991218 | 162105.24 | 424 | 0 | SNAPC2 | 3.76 |
| 886 | chr19 | 7967414 | 7991218 | 162105.24 | 424 | 0 | CTXN1 | 3.04 |
| 887 | chr19 | 7967414 | 7991218 | 162105.24 | 424 | 0 | LRRC8E | 1.33 |
| 888 | chr19 | 7967414 | 7991218 | 162105.24 | 424 | 0 | TGFBR3L | 1.14 |
| 889 | chr20 | 270349 | 291211 | 161471.88 | 425 | 0 | NRSN2 | 4.60 |
| 890 | chr20 | 270349 | 291211 | 161471.88 | 425 | 0 | SOX12 | 4.44 |
| 891 | chr20 | 270349 | 291211 | 161471.88 | 425 | 0 | ZCCHC3 | 4.11 |
| 892 | chr20 | 270349 | 291211 | 161471.88 | 425 | 0 | C20orf96 | 3.51 |
| 893 | chr17 | 46124184 | 46134697 | 161374.55 | 426 | 0 | NFE2L1 | 6.34 |
| 894 | chr17 | 46124184 | 46134697 | 161374.55 | 426 | 0 | CBX1 | 4.42 |
| 895 | chr21 | 15578884 | 15589819 | 161400.60 | 427 | 0 | RBM11 | 5.04 |
| 896 | chr21 | 15578884 | 15589819 | 161400.60 | 427 | 0 | LIPI | 4.95 |
| 897 | chr18 | 3830690 | 3844392 | 160724.46 | 428 | 0 | na | na |
| 898 | chr16 | 25809440 | 25822509 | 160487.32 | 429 | 0 | na | na |
| 899 | chr11 | 129871098 | 129874113 | 160156.80 | 430 | 0 | na | na |
| 900 | chr9 | 37983082 | 37999333 | 160072.35 | 431 | 0 | na | na |
| 901 | chr9 | 86593804 | 86596873 | 159894.90 | 432 | 0 | na | na |
| 902 | chr3 | 197806060 | 197817962 | 159724.84 | 433 | 0 | ANKRD18DP | 2.64 |
| 903 | chr6 | 70479533 | 70487759 | 159584.40 | 434 | 0 | LMBRD1 | 5.51 |
| 904 | chr2 | 10259861 | 10264024 | 159401.27 | 435 | 0 | RRM2 | 4.94 |
| 905 | chr12 | 120719898 | 120740596 | 159167.62 | 436 | 0 | PXN | 3.65 |
| 906 | chr7 | 89836078 | 89842715 | 159221.63 | 437 | 0 | STEAP2 | 4.77 |
| 907 | chr19 | 40646754 | 40654889 | 159201.95 | 438 | 0 | MAP3K10 | 2.89 |
| 908 | chr6 | 107435318 | 107440570 | 159135.60 | 439 | 0 | BEND3 | 1.72 |
| 909 | chr14 | 21565499 | 21575700 | 158931.58 | 440 | 0 | ARHGEF40 | 4.66 |
| 910 | chr14 | 21565499 | 21575700 | 158931.58 | 440 | 0 | ZNF219 | 1.46 |
| 911 | chr17 | 79631710 | 79635395 | 158897.20 | 441 | 0 | OXLD1 | 4.98 |
| 912 | chr17 | 79631710 | 79635395 | 158897.20 | 441 | 0 | CCDC137 | 4.72 |
| 913 | chr17 | 79631710 | 79635395 | 158897.20 | 441 | 0 | NPLOC4 | 3.65 |
| 914 | chr6 | 31703491 | 31708851 | 158066.40 | 442 | 0 | CLIC1 | 5.54 |
| 915 | chr6 | 31703491 | 31708851 | 158066.40 | 442 | 0 | ABHD16A | 4.75 |
| 916 | chr6 | 31703491 | 31708851 | 158066.40 | 442 | 0 | DDAH2 | 2.11 |
| 917 | chr6 | 31703491 | 31708851 | 158066.40 | 442 | 0 | MSH5 | 2.10 |
| 918 | chr7 | 100463295 | 100473666 | 158054.04 | 443 | 0 | SRRT | 6.05 |
| 919 | chr7 | 100463295 | 100473666 | 158054.04 | 443 | 0 | TRIP6 | 5.31 |
| 920 | chr7 | 100463295 | 100473666 | 158054.04 | 443 | 0 | EPHB4 | 5.18 |
| 921 | chr7 | 100463295 | 100473666 | 158054.04 | 443 | 0 | SLC12A9 | 3.75 |
| 922 | chr11 | 10422475 | 10437227 | 157993.92 | 444 | 0 | na | na |
| 923 | chr8 | 117878327 | 117887769 | 157870.24 | 445 | 0 | RAD21 | 5.65 |
| 924 | chr16 | 89766860 | 89789367 | 157774.07 | 446 | 0 | ZNF276 | 4.32 |
| 925 | chr16 | 89766860 | 89789367 | 157774.07 | 446 | 0 | CDK10 | 3.72 |
| 926 | chr16 | 89766860 | 89789367 | 157774.07 | 446 | 0 | SPATA2L | 1.86 |
| 927 | chr6 | 20401020 | 20404991 | 157370.73 | 447 | 0 | E2F3 | 3.22 |
| 928 | chr7 | 136075888 | 136084579 | 157307.10 | 448 | 0 | na | na |
| 929 | chr17 | 57904926 | 57916690 | 157284.68 | 449 | 0 | na | na |
| 930 | chr17 | 7136909 | 7156293 | 157204.24 | 450 | 0 | ACADVL | 6.46 |
| 931 | chr17 | 7136909 | 7156293 | 157204.24 | 450 | 0 | CTDNEP1 | 5.21 |
| 932 | chr17 | 7136909 | 7156293 | 157204.24 | 450 | 0 | GABARAP | 3.88 |
| 933 | chr17 | 7136909 | 7156293 | 157204.24 | 450 | 0 | DVL2 | 3.69 |
| 934 | chr17 | 7136909 | 7156293 | 157204.24 | 450 | 0 | DLG4 | 3.48 |
| 935 | chr17 | 7136909 | 7156293 | 157204.24 | 450 | 0 | PHF23 | 2.96 |
| 936 | chr17 | 7136909 | 7156293 | 157204.24 | 450 | 0 | SLC2A4 | 1.02 |
| 937 | chr9 | 139000909 | 139012011 | 156871.26 | 451 | 0 | C9orf69 | 3.29 |
| 938 | chr9 | 139000909 | 139012011 | 156871.26 | 451 | 0 | NACC2 | 2.21 |
| 939 | chr19 | 2423884 | 2428613 | 156813.64 | 452 | 0 | TIMM13 | 7.07 |
| 940 | chr19 | 2423884 | 2428613 | 156813.64 | 452 | 0 | LMNB2 | 4.76 |
| 941 | chr19 | 2423884 | 2428613 | 156813.64 | 452 | 0 | GADD45B | 1.15 |
| 942 | chr3 | 44036242 | 44041295 | 156643.00 | 453 | 0 | na | na |
| 943 | chr12 | 46765247 | 46778449 | 156443.70 | 454 | 0 | SLC38A2 | 7.65 |
| 944 | chr1 | 46766499 | 46771092 | 156162.00 | 455 | 0 | UQCRH | 5.97 |
| 945 | chr1 | 46766499 | 46771092 | 156162.00 | 455 | 0 | LRRC41 | 3.76 |
| 946 | chr1 | 46766499 | 46771092 | 156162.00 | 455 | 0 | NSUN4 | 2.41 |
| 947 | chr1 | 41826972 | 41848667 | 155987.05 | 456 | 0 | na | na |
| 948 | chr17 | 27037513 | 27056115 | 156070.78 | 457 | 0 | RPL23A | 6.52 |
| 949 | chr17 | 27037513 | 27056115 | 156070.78 | 457 | 0 | RAB34 | 4.15 |
| 950 | chr17 | 27037513 | 27056115 | 156070.78 | 457 | 0 | TLCD1 | 3.17 |
| 951 | chr17 | 27037513 | 27056115 | 156070.78 | 457 | 0 | TRAF4 | 2.42 |
| 952 | chr17 | 27037513 | 27056115 | 156070.78 | 457 | 0 | PROCA1 | 1.05 |
| 953 | chr7 | 100025797 | 100034772 | 155985.50 | 458 | 0 | MEPCE | 4.29 |
| 954 | chr7 | 100025797 | 100034772 | 155985.50 | 458 | 0 | TSC22D4 | 4.00 |
| 955 | chr7 | 100025797 | 100034772 | 155985.50 | 458 | 0 | PPP1R35 | 3.63 |
| 956 | chr6 | 33377361 | 33399763 | 155917.92 | 459 | 0 | CUTA | 6.47 |
| 957 | chr6 | 33377361 | 33399763 | 155917.92 | 459 | 0 | KIFC1 | 4.27 |
| 958 | chr6 | 33377361 | 33399763 | 155917.92 | 459 | 0 | SYNGAP1 | 3.83 |
| 959 | chr6 | 33377361 | 33399763 | 155917.92 | 459 | 0 | PHF1 | 3.25 |
| 960 | chr6 | 33377361 | 33399763 | 155917.92 | 459 | 0 | ZBTB9 | 3.11 |
| 961 | chr1 | 245025536 | 245029004 | 155678.52 | 460 | 0 | HNRNPU | 6.72 |
| 962 | chr1 | 245025536 | 245029004 | 155678.52 | 460 | 0 | COX20 | 3.72 |
| 963 | chr6 | 86351296 | 86354749 | 155523.12 | 461 | 0 | SNHG5 | 7.07 |
| 964 | chr6 | 86351296 | 86354749 | 155523.12 | 461 | 0 | SYNCRIP | 4.64 |
| 965 | chr6 | 86351296 | 86354749 | 155523.12 | 461 | 0 | SNX14 | 3.01 |
| 966 | chr1 | 145382199 | 145413668 | 155142.17 | 462 | 0 | TXNIP | 4.20 |
| 967 | chr8 | 57114924 | 57125182 | 155203.54 | 463 | 0 | CHCHD7 | 3.62 |
| 968 | chr8 | 57114924 | 57125182 | 155203.54 | 463 | 0 | PLAG1 | 2.86 |
| 969 | chr17 | 48226558 | 48238966 | 154851.84 | 464 | 0 | PPP1R9B | 4.70 |
| 970 | chr17 | 48226558 | 48238966 | 154851.84 | 464 | 0 | COL1A1 | 2.09 |
| 971 | chr17 | 48226558 | 48238966 | 154851.84 | 464 | 0 | SAMD14 | 1.49 |
| 972 | chr16 | 30537427 | 30547707 | 154611.20 | 465 | 0 | ZNF768 | 2.51 |
| 973 | chr7 | 135956859 | 135964008 | 154203.93 | 466 | 0 | na | na |
| 974 | chr1 | 150227919 | 150245619 | 154167.00 | 467 | 0 | APH1A | 5.63 |
| 975 | chr1 | 150227919 | 150245619 | 154167.00 | 467 | 0 | MRPS21 | 5.47 |
| 976 | chr1 | 150227919 | 150245619 | 154167.00 | 467 | 0 | CA14 | 4.32 |
| 977 | chr1 | 150227919 | 150245619 | 154167.00 | 467 | 0 | C1orf54 | 4.23 |
| 978 | chr12 | 16869714 | 16881640 | 153964.66 | 468 | 0 | na | na |
| 979 | chr11 | 62553968 | 62573735 | 153589.59 | 469 | 0 | TMEM179B | 6.24 |
| 980 | chr11 | 62553968 | 62573735 | 153589.59 | 469 | 0 | NXF1 | 4.52 |
| 981 | chr11 | 62553968 | 62573735 | 153589.59 | 469 | 0 | POLR2G | 4.52 |
| 982 | chr11 | 62553968 | 62573735 | 153589.59 | 469 | 0 | TMEM223 | 4.29 |
| 983 | chr11 | 62553968 | 62573735 | 153589.59 | 469 | 0 | TAF6L | 3.72 |
| 984 | chr11 | 62553968 | 62573735 | 153589.59 | 469 | 0 | ZBTB3 | 1.59 |
| 985 | chr12 | 53885967 | 53897219 | 153364.76 | 470 | 0 | PCBP2 | 6.30 |
| 986 | chr12 | 53885967 | 53897219 | 153364.76 | 470 | 0 | TARBP2 | 3.85 |
| 987 | chr12 | 53885967 | 53897219 | 153364.76 | 470 | 0 | MAP3K12 | 3.81 |
| 988 | chr17 | 42294352 | 42299484 | 153395.48 | 471 | 0 | UBTF | 4.80 |
| 989 | chr17 | 42294352 | 42299484 | 153395.48 | 471 | 0 | ATXN7L3 | 3.48 |
| 990 | chr17 | 42294352 | 42299484 | 153395.48 | 471 | 0 | TMUB2 | 2.98 |
| 991 | chr13 | 103045988 | 103054844 | 153208.80 | 472 | 0 | na | na |
| 992 | chr14 | 69258853 | 69262994 | 153092.77 | 473 | 0 | ZFP36L1 | 2.31 |
| 993 | chr21 | 38737691 | 38740914 | 153060.27 | 474 | 0 | DYRK1A | 2.55 |
| 994 | chr13 | 93878310 | 93883133 | 153033.79 | 475 | 0 | GPC6 | 4.29 |
| 995 | chr11 | 65624447 | 65640573 | 152874.48 | 476 | 0 | CFL1 | 5.81 |
| 996 | chr11 | 65624447 | 65640573 | 152874.48 | 476 | 0 | FIBP | 4.52 |
| 997 | chr11 | 65624447 | 65640573 | 152874.48 | 476 | 0 | CCDC85B | 4.19 |
| 998 | chr11 | 65624447 | 65640573 | 152874.48 | 476 | 0 | MUS81 | 3.79 |
| 999 | chr11 | 65624447 | 65640573 | 152874.48 | 476 | 0 | EFEMP2 | 3.31 |
| 1000 | chr16 | 49887678 | 49893454 | 152717.44 | 477 | 0 | na | na |
| 1001 | chr7 | 30376175 | 30394086 | 152780.83 | 478 | 0 | na | na |
| 1002 | chr12 | 109529542 | 109549889 | 152195.56 | 479 | 0 | UNG | 5.03 |
| 1003 | chr12 | 109529542 | 109549889 | 152195.56 | 479 | 0 | ALKBH2 | 4.91 |
| 1004 | chr12 | 109529542 | 109549889 | 152195.56 | 479 | 0 | USP30 | 3.06 |
| 1005 | chr11 | 37285972 | 37302483 | 152231.42 | 480 | 0 | na | na |
| 1006 | chr13 | 39610596 | 39626235 | 152167.47 | 481 | 0 | NHLRC3 | 3.99 |
| 1007 | chr13 | 39610596 | 39626235 | 152167.47 | 481 | 0 | PROSER1 | 3.43 |
| 1008 | chr22 | 41840554 | 41844593 | 151947.18 | 482 | 0 | ACO2 | 5.94 |
| 1009 | chr22 | 41840554 | 41844593 | 151947.18 | 482 | 0 | PHF5A | 3.70 |
| 1010 | chr22 | 41840554 | 41844593 | 151947.18 | 482 | 0 | TOB2 | 3.61 |
| 1011 | chr3 | 100327830 | 100335217 | 151802.85 | 483 | 0 | na | na |
| 1012 | chr6 | 146054759 | 146057597 | 151776.24 | 484 | 0 | na | na |
| 1013 | chr17 | 17727119 | 17753099 | 150943.80 | 485 | 0 | SREBF1 | 3.85 |
| 1014 | chr2 | 74681539 | 74710645 | 150769.08 | 486 | 0 | WDR54 | 5.04 |
| 1015 | chr2 | 74681539 | 74710645 | 150769.08 | 486 | 0 | MOGS | 5.03 |
| 1016 | chr2 | 74681539 | 74710645 | 150769.08 | 486 | 0 | INO80B | 4.69 |
| 1017 | chr2 | 74681539 | 74710645 | 150769.08 | 486 | 0 | TTC31 | 3.62 |
| 1018 | chr2 | 74681539 | 74710645 | 150769.08 | 486 | 0 | WBP1 | 3.59 |
| 1019 | chr2 | 74681539 | 74710645 | 150769.08 | 486 | 0 | RTKN | 2.91 |
| 1020 | chr2 | 74681539 | 74710645 | 150769.08 | 486 | 0 | PCGF1 | 2.67 |
| 1021 | chr2 | 74681539 | 74710645 | 150769.08 | 486 | 0 | CCDC142 | 2.23 |
| 1022 | chr4 | 190935449 | 190942919 | 150744.60 | 487 | 0 | na | na |
| 1023 | chr11 | 110220455 | 110234528 | 150721.83 | 488 | 0 | na | na |
| 1024 | chr13 | 34007184 | 34022069 | 150487.35 | 489 | 0 | na | na |
| 1025 | chr9 | 123630416 | 123640716 | 150483.00 | 490 | 0 | PSMD5 | 2.99 |
| 1026 | chr9 | 123630416 | 123640716 | 150483.00 | 490 | 0 | PHF19 | 2.93 |
| 1027 | chr13 | 60969918 | 60974106 | 150223.56 | 491 | 0 | TDRD3 | 2.26 |
| 1028 | chr17 | 79478607 | 79485264 | 149982.21 | 492 | 0 | ACTG1 | 9.44 |
| 1029 | chr17 | 79478607 | 79485264 | 149982.21 | 492 | 0 | C17orf70 | 4.03 |
| 1030 | chr7 | 104652205 | 104656346 | 149945.61 | 493 | 0 | na | na |
| 1031 | chr20 | 57463501 | 57468034 | 149951.64 | 494 | 0 | na | na |
| 1032 | chr2 | 241496385 | 241508799 | 149961.12 | 495 | 0 | RNPEPL1 | 3.73 |
| 1033 | chr2 | 241496385 | 241508799 | 149961.12 | 495 | 0 | CAPN10 | 3.05 |
| 1034 | chr2 | 241496385 | 241508799 | 149961.12 | 495 | 0 | ANKMY1 | 2.01 |
| 1035 | chr2 | 241496385 | 241508799 | 149961.12 | 495 | 0 | DUSP28 | 1.66 |
| 1036 | chr9 | 139733994 | 139744193 | 149619.33 | 496 | 0 | EDF1 | 6.00 |
| 1037 | chr9 | 139733994 | 139744193 | 149619.33 | 496 | 0 | PHPT1 | 4.88 |
| 1038 | chr9 | 139733994 | 139744193 | 149619.33 | 496 | 0 | MAMDC4 | 3.50 |
| 1039 | chr9 | 139733994 | 139744193 | 149619.33 | 496 | 0 | C9orf172 | 1.99 |
| 1040 | chr9 | 139733994 | 139744193 | 149619.33 | 496 | 0 | TRAF2 | 1.85 |
| 1041 | chr16 | 2008636 | 2034859 | 149471.10 | 497 | 0 | RPS2 | 9.66 |
| 1042 | chr16 | 2008636 | 2034859 | 149471.10 | 497 | 0 | NPW | 7.83 |
| 1043 | chr16 | 2008636 | 2034859 | 149471.10 | 497 | 0 | NDUFB10 | 5.66 |
| 1044 | chr16 | 2008636 | 2034859 | 149471.10 | 497 | 0 | TBL3 | 4.68 |
| 1045 | chr16 | 2008636 | 2034859 | 149471.10 | 497 | 0 | ZNF598 | 4.49 |
| 1046 | chr16 | 2008636 | 2034859 | 149471.10 | 497 | 0 | SNORA10 | 3.30 |
| 1047 | chr16 | 2008636 | 2034859 | 149471.10 | 497 | 0 | SNORA64 | 3.27 |
| 1048 | chr16 | 2008636 | 2034859 | 149471.10 | 497 | 0 | GFER | 1.44 |
| 1049 | chr16 | 2008636 | 2034859 | 149471.10 | 497 | 0 | SNHG9 | 1.38 |
| 1050 | chr16 | 2008636 | 2034859 | 149471.10 | 497 | 0 | MSRB1 | 1.28 |
| 1051 | chr6 | 34191833 | 34217486 | 149556.99 | 498 | 0 | HMGA1 | 6.44 |
| 1052 | chr6 | 34191833 | 34217486 | 149556.99 | 498 | 0 | C6orf1 | 1.61 |
| 1053 | chr12 | 125398560 | 125405948 | 149533.12 | 499 | 0 | UBC | 8.07 |
| 1054 | chr3 | 156323156 | 156333087 | 149461.55 | 500 | 0 | na | na |
