# Supplementary material for: Functional, chemical genomic, and super-enhancer screening identify sensitivity to cyclin D1/CDK4 pathway inhibition in Ewing sarcoma
Source: Oncotarget. 2015 Aug 18;6(30):30178–93. doi: 10.18632/oncotarget.4903 (PMC4745789; doi:10.18632/oncotarget.4903)
Supplement: Supplementary file 4 [file oncotarget-06-30178-s004.doc]

| Rank  **Supplementary Table 4**. List of 425 significant Ewing Sarcoma gene dependencies identified based on the ATARiS method in the Achilles v2.4.3 data. The dependencies were estimated for five Ewing cell lines (A673, EW8, EWS502, TC71, CADO-ES-1) vs all other 211 tumor cell lines. Significance was assessed based on the adjusted P-value cut-off 0.05. | ATARiS_Solution | Gene | Signal to noise | P-value.adj |
| --- | --- | --- | --- | --- |
| 1 | SHPRH_1_00011 | SHPRH | -1.44 | 0.00 |
| 2 | LSAMP_1_00111 | LSAMP | -1.39 | 0.00 |
| 3 | DNAJC16_1_1001 | DNAJC16 | -1.28 | 0.00 |
| 4 | STRN4_1_10011 | STRN4 | -1.27 | 0.00 |
| 5 | BARHL1_1_01011 | BARHL1 | -1.19 | 0.00 |
| 6 | FRMPD2P1_1_11 | FRMPD2P1 | -1.19 | 0.00 |
| 7 | DCLRE1B_1_00111 | DCLRE1B | -1.18 | 0.00 |
| 8 | RNF17_1_001101011 | RNF17 | -1.17 | 0.00 |
| 9 | WTAP_1_01110 | WTAP | -1.16 | 0.00 |
| 10 | EPHB1_1_10010 | EPHB1 | -1.16 | 0.00 |
| 11 | AGFG1_1_10001 | AGFG1 | -1.14 | 0.00 |
| 12 | S100A10_1_01101 | S100A10 | -1.13 | 0.00 |
| 13 | WFDC10B_1_01111 | WFDC10B | -1.11 | 0.00 |
| 14 | IMPACT_1_1011 | IMPACT | -1.10 | 0.00 |
| 15 | CKLF_1_1111 | CKLF | -1.10 | 0.00 |
| 16 | IL28A_1_0011101 | IL28A | -1.10 | 0.00 |
| 17 | KRT3_2_0000111 | KRT3 | -1.10 | 0.00 |
| 18 | AKAP10_1_1101 | AKAP10 | -1.09 | 0.00 |
| 19 | GPR135_1_0011 | GPR135 | -1.08 | 0.00 |
| 20 | SLC12A3_1_0111 | SLC12A3 | -1.08 | 0.00 |
| 21 | TRPM2_1_00110 | TRPM2 | -1.07 | 0.00 |
| 22 | PPP2R4_1_1011 | PPP2R4 | -1.07 | 0.00 |
| 23 | SPR_1_10101 | SPR | -1.05 | 0.00 |
| 24 | HIST1H2AM_1_0110 | HIST1H2AM | -0.99 | 0.00 |
| 25 | SMAD4_1_1011111 | SMAD4 | -0.99 | 0.00 |
| 26 | OR2G3_1_11001 | OR2G3 | -0.97 | 0.00 |
| 27 | ACTR6_2_01001 | ACTR6 | -0.95 | 0.00 |
| 28 | LTBR_1_11111 | LTBR | -0.95 | 0.00 |
| 29 | ESRRA_1_11110 | ESRRA | -0.95 | 0.00 |
| 30 | MOCS2_1_1110 | MOCS2 | -0.94 | 0.00 |
| 31 | IL28B_1_0011010 | IL28B | -0.94 | 0.00 |
| 32 | INSM2_1_01110 | INSM2 | -0.93 | 0.00 |
| 33 | GSK3A_1_0110010101 | GSK3A | -0.93 | 0.00 |
| 34 | ATP1A3_1_01001 | ATP1A3 | -0.92 | 0.00 |
| 35 | RPS6KL1_1_01110 | RPS6KL1 | -0.92 | 0.00 |
| 36 | SLC5A3_1_00111 | SLC5A3 | -0.91 | 0.00 |
| 37 | HIST1H2AK_1_0101010 | HIST1H2AK | -0.91 | 0.00 |
| 38 | SATL1_1_00011 | SATL1 | -0.91 | 0.00 |
| 39 | BATF_1_110 | BATF | -0.90 | 0.00 |
| 40 | UGT1A7_1_0001011 | UGT1A7 | -0.90 | 0.00 |
| 41 | SNX2_1_0110 | SNX2 | -0.90 | 0.00 |
| 42 | AANAT_1_10101 | AANAT | -0.89 | 0.00 |
| 43 | MAPT_1_111 | MAPT | -0.89 | 0.00 |
| 44 | TRIM43_1_10111 | TRIM43 | -0.87 | 0.01 |
| 45 | TRIM43B_1_10111 | TRIM43B | -0.87 | 0.01 |
| 46 | MYNN_1_10100 | MYNN | -0.87 | 0.00 |
| 47 | C17orf85_1_1010 | C17orf85 | -0.87 | 0.00 |
| 48 | RBMS1_1_01111 | RBMS1 | -0.87 | 0.00 |
| 49 | IL16_1_01001 | IL16 | -0.86 | 0.00 |
| 50 | CITED2_1_11011 | CITED2 | -0.86 | 0.00 |
| 51 | KRT28_1_00101 | KRT28 | -0.85 | 0.00 |
| 52 | PIK3CB_1_11111001 | PIK3CB | -0.85 | 0.00 |
| 53 | KAT2B_1_11100 | KAT2B | -0.85 | 0.00 |
| 54 | COBL_1_00111 | COBL | -0.85 | 0.00 |
| 55 | DNAJC10_1_10011 | DNAJC10 | -0.85 | 0.01 |
| 56 | ARFGAP3_1_01101 | ARFGAP3 | -0.85 | 0.00 |
| 57 | PPAP2C_2_1010 | PPAP2C | -0.85 | 0.00 |
| 58 | MECP2_1_1101 | MECP2 | -0.84 | 0.00 |
| 59 | HSD17B1_1_1111 | HSD17B1 | -0.84 | 0.00 |
| 60 | DIAPH2_1_0011 | DIAPH2 | -0.84 | 0.00 |
| 61 | ANXA4_1_01011 | ANXA4 | -0.84 | 0.00 |
| 62 | PRKAR2A_1_01010 | PRKAR2A | -0.84 | 0.00 |
| 63 | NMNAT1_1_101111 | NMNAT1 | -0.84 | 0.00 |
| 64 | CD99_1_10101 | CD99 | -0.84 | 0.00 |
| 65 | UGT1A4_1_10100011 | UGT1A4 | -0.83 | 0.00 |
| 66 | TPO_1_01001 | TPO | -0.83 | 0.01 |
| 67 | TCN2_1_11101 | TCN2 | -0.83 | 0.00 |
| 68 | PSEN2_1_1011 | PSEN2 | -0.82 | 0.01 |
| 69 | VTA1_1_111 | VTA1 | -0.82 | 0.01 |
| 70 | RGSL1_1_01000001 | RGSL1 | -0.82 | 0.00 |
| 71 | CECR1_1_10001 | CECR1 | -0.81 | 0.00 |
| 72 | MKRN1_1_100110 | MKRN1 | -0.80 | 0.00 |
| 73 | ZNF573_1_101 | ZNF573 | -0.80 | 0.01 |
| 74 | SGPP2_1_00110 | SGPP2 | -0.79 | 0.01 |
| 75 | CRADD_1_1011 | CRADD | -0.79 | 0.00 |
| 76 | DAP_1_0111 | DAP | -0.79 | 0.01 |
| 77 | INTS6_1_00011 | INTS6 | -0.79 | 0.00 |
| 78 | RAB15_1_00011 | RAB15 | -0.79 | 0.01 |
| 79 | IDH3B_1_11011 | IDH3B | -0.79 | 0.00 |
| 80 | DAB1_1_01111 | DAB1 | -0.78 | 0.00 |
| 81 | BMP2K_1_010101 | BMP2K | -0.78 | 0.00 |
| 82 | GPR143_1_011 | GPR143 | -0.78 | 0.00 |
| 83 | SSBP4_1_111 | SSBP4 | -0.78 | 0.01 |
| 84 | COL7A1_1_1111 | COL7A1 | -0.78 | 0.00 |
| 85 | RALGAPA1_1_11010 | RALGAPA1 | -0.78 | 0.00 |
| 86 | PCK1_1_01100 | PCK1 | -0.77 | 0.01 |
| 87 | SLC6A16_1_10111 | SLC6A16 | -0.77 | 0.00 |
| 88 | FECH_1_01111 | FECH | -0.77 | 0.00 |
| 89 | PRB3_1_110111 | PRB3 | -0.77 | 0.01 |
| 90 | LILRA3_1_00101 | LILRA3 | -0.77 | 0.01 |
| 91 | LOC100653070_1_00101 | LOC100653070 | -0.77 | 0.01 |
| 92 | RGS18_1_00111 | RGS18 | -0.77 | 0.01 |
| 93 | HIST1H2BB_1_101011111 | HIST1H2BB | -0.76 | 0.01 |
| 94 | AMY2B_1_1001101101 | AMY2B | -0.76 | 0.00 |
| 95 | BIRC2_1_01111 | BIRC2 | -0.76 | 0.01 |
| 96 | CCND1_1_001101 | CCND1 | -0.76 | 0.01 |
| 97 | AGPS_1_1011 | AGPS | -0.76 | 0.01 |
| 98 | HNRNPH1_1_1111 | HNRNPH1 | -0.76 | 0.01 |
| 99 | DHX38_1_11101 | DHX38 | -0.76 | 0.01 |
| 100 | RNF112_1_10110 | RNF112 | -0.75 | 0.01 |
| 101 | GPR151_1_01001 | GPR151 | -0.75 | 0.01 |
| 102 | KIAA1804_1_11101 | KIAA1804 | -0.75 | 0.01 |
| 103 | CBX2_1_11110 | CBX2 | -0.74 | 0.01 |
| 104 | FLJ40852_1_01111 | FLJ40852 | -0.74 | 0.01 |
| 105 | TGIF2_1_1110 | TGIF2 | -0.74 | 0.00 |
| 106 | TMEM9_1_01111 | TMEM9 | -0.73 | 0.01 |
| 107 | NUMA1_1_0111 | NUMA1 | -0.73 | 0.00 |
| 108 | RABL2B_1_001001011 | RABL2B | -0.73 | 0.01 |
| 109 | RABL2A_1_0101011 | RABL2A | -0.73 | 0.01 |
| 110 | PIP4K2C_1_00111 | PIP4K2C | -0.73 | 0.01 |
| 111 | DTX3L_1_10110 | DTX3L | -0.73 | 0.01 |
| 112 | ISL1_1_00011 | ISL1 | -0.73 | 0.01 |
| 113 | PARP10_1_1001 | PARP10 | -0.73 | 0.00 |
| 114 | PCDHA10_1_100011 | PCDHA10 | -0.73 | 0.01 |
| 115 | IRF2BPL_1_0101 | IRF2BPL | -0.73 | 0.01 |
| 116 | IL2RA_2_10110 | IL2RA | -0.72 | 0.01 |
| 117 | GLS2_1_10010 | GLS2 | -0.72 | 0.01 |
| 118 | SLC1A5_1_01101 | SLC1A5 | -0.72 | 0.01 |
| 119 | ACBD3_1_11001 | ACBD3 | -0.72 | 0.01 |
| 120 | WIF1_1_1111 | WIF1 | -0.72 | 0.02 |
| 121 | POMC_1_10110 | POMC | -0.72 | 0.01 |
| 122 | QDPR_1_110 | QDPR | -0.72 | 0.01 |
| 123 | LIMD1_1_000101 | LIMD1 | -0.71 | 0.01 |
| 124 | TOX2_1_01111 | TOX2 | -0.71 | 0.01 |
| 125 | MELK_1_10111 | MELK | -0.71 | 0.01 |
| 126 | P2RY11_1_11111 | P2RY11 | -0.71 | 0.01 |
| 127 | PPAN-P2RY11_1_11111 | PPAN-P2RY11 | -0.71 | 0.01 |
| 128 | SETBP1_1_00101 | SETBP1 | -0.70 | 0.00 |
| 129 | NEIL2_1_11111 | NEIL2 | -0.70 | 0.01 |
| 130 | FFAR2_1_10101 | FFAR2 | -0.70 | 0.01 |
| 131 | POC1B-GALNT4_1_101111 | POC1B-GALNT4 | -0.70 | 0.00 |
| 132 | SSBP2_1_01110 | SSBP2 | -0.70 | 0.02 |
| 133 | PIP5KL1_1_10110 | PIP5KL1 | -0.70 | 0.01 |
| 134 | DLX6_1_0010100001 | DLX6 | -0.70 | 0.01 |
| 135 | UVRAG_1_0101 | UVRAG | -0.70 | 0.03 |
| 136 | MMP14_1_10011 | MMP14 | -0.69 | 0.01 |
| 137 | SGIP1_1_11100 | SGIP1 | -0.69 | 0.01 |
| 138 | TMLHE_1_00101 | TMLHE | -0.69 | 0.01 |
| 139 | LALBA_1_11111 | LALBA | -0.69 | 0.02 |
| 140 | ITGB1_1_1011 | ITGB1 | -0.69 | 0.01 |
| 141 | IGF1R_1_1011110101111 | IGF1R | -0.69 | 0.00 |
| 142 | SACS_1_11101 | SACS | -0.68 | 0.01 |
| 143 | NFKBIA_1_1111 | NFKBIA | -0.68 | 0.01 |
| 144 | HCFC2_1_11001 | HCFC2 | -0.68 | 0.01 |
| 145 | PROC_1_11111 | PROC | -0.68 | 0.01 |
| 146 | TCEA3_1_0101 | TCEA3 | -0.68 | 0.01 |
| 147 | ZNF91_1_0100001 | ZNF91 | -0.68 | 0.01 |
| 148 | WSB2_1_11111 | WSB2 | -0.68 | 0.01 |
| 149 | CD96_1_10001 | CD96 | -0.67 | 0.02 |
| 150 | TM7SF2_1_01010 | TM7SF2 | -0.67 | 0.01 |
| 151 | THRA_1_10111 | THRA | -0.67 | 0.01 |
| 152 | MLL_1_1100 | MLL | -0.67 | 0.02 |
| 153 | IFNA5_1_11011111010 | IFNA5 | -0.67 | 0.02 |
| 154 | CTPS2_1_01110 | CTPS2 | -0.67 | 0.00 |
| 155 | CYP3A7_1_011000 | CYP3A7 | -0.67 | 0.01 |
| 156 | CYP3A7-CYP3AP1_1_011000 | CYP3A7-CYP3AP1 | -0.67 | 0.01 |
| 157 | LCN15_1_101 | LCN15 | -0.67 | 0.01 |
| 158 | FAF2_1_1010 | FAF2 | -0.66 | 0.02 |
| 159 | CDKN1A_1_11111 | CDKN1A | -0.66 | 0.01 |
| 160 | RNF24_1_1011 | RNF24 | -0.66 | 0.01 |
| 161 | RPP21_1_1111 | RPP21 | -0.66 | 0.01 |
| 162 | TRIM39-RPP21_1_1100110 | TRIM39-RPP21 | -0.66 | 0.01 |
| 163 | SULT1A1_1_1110100100 | SULT1A1 | -0.66 | 0.02 |
| 164 | RBM22_1_0111 | RBM22 | -0.66 | 0.01 |
| 165 | CPA6_1_11011 | CPA6 | -0.66 | 0.01 |
| 166 | AKAP3_1_1011 | AKAP3 | -0.66 | 0.01 |
| 167 | ABHD14A_1_10110 | ABHD14A | -0.66 | 0.01 |
| 168 | HTR2B_1_00110 | HTR2B | -0.66 | 0.01 |
| 169 | SRCRB4D_1_1111 | SRCRB4D | -0.66 | 0.02 |
| 170 | RAC3_1_10011 | RAC3 | -0.66 | 0.02 |
| 171 | USO1_1_00011 | USO1 | -0.65 | 0.01 |
| 172 | SPTLC1_1_00101 | SPTLC1 | -0.65 | 0.01 |
| 173 | CYP3A5_1_011110 | CYP3A5 | -0.65 | 0.02 |
| 174 | IKZF4_1_11001 | IKZF4 | -0.65 | 0.01 |
| 175 | HNRNPA3P1_1_0111111011 | HNRNPA3P1 | -0.65 | 0.01 |
| 176 | IFNA6_1_1001 | IFNA6 | -0.65 | 0.02 |
| 177 | UBB_1_1100 | UBB | -0.65 | 0.01 |
| 178 | PTGFR_1_01110 | PTGFR | -0.65 | 0.01 |
| 179 | OR8G5_1_01111 | OR8G5 | -0.65 | 0.01 |
| 180 | CLTA_1_01111 | CLTA | -0.64 | 0.01 |
| 181 | CDKL4_1_1011 | CDKL4 | -0.64 | 0.01 |
| 182 | SLCO4A1_1_00110 | SLCO4A1 | -0.64 | 0.01 |
| 183 | SLC12A2_1_01011 | SLC12A2 | -0.64 | 0.02 |
| 184 | HLA-DMB_1_1111 | HLA-DMB | -0.64 | 0.00 |
| 185 | RRAGB_1_1011 | RRAGB | -0.64 | 0.02 |
| 186 | CHD9_1_00011 | CHD9 | -0.64 | 0.01 |
| 187 | IQGAP2_1_1011 | IQGAP2 | -0.64 | 0.03 |
| 188 | ZCCHC9_1_0111 | ZCCHC9 | -0.64 | 0.02 |
| 189 | IL12RB1_1_100111 | IL12RB1 | -0.63 | 0.02 |
| 190 | HIST1H2BL_1_10111 | HIST1H2BL | -0.63 | 0.01 |
| 191 | CDK4_1_11101 | CDK4 | -0.63 | 0.01 |
| 192 | GAP43_1_10001 | GAP43 | -0.63 | 0.02 |
| 193 | PAPL_1_01011 | PAPL | -0.63 | 0.03 |
| 194 | LYRM2_1_00101 | LYRM2 | -0.63 | 0.02 |
| 195 | KIF5B_1_11111 | KIF5B | -0.63 | 0.02 |
| 196 | SLC17A7_1_01010 | SLC17A7 | -0.63 | 0.01 |
| 197 | SS18L1_1_01101 | SS18L1 | -0.63 | 0.02 |
| 198 | PROKR2_1_10011 | PROKR2 | -0.63 | 0.02 |
| 199 | ATP5F1_1_1111 | ATP5F1 | -0.63 | 0.01 |
| 200 | ABL1_1_1110001111 | ABL1 | -0.62 | 0.01 |
| 201 | CLK2_1_11111111 | CLK2 | -0.62 | 0.01 |
| 202 | MAP3K14_1_0101 | MAP3K14 | -0.62 | 0.01 |
| 203 | RETSAT_1_01100 | RETSAT | -0.62 | 0.02 |
| 204 | FRMPD2_1_11001 | FRMPD2 | -0.62 | 0.01 |
| 205 | AKR7A2_1_110111110 | AKR7A2 | -0.62 | 0.03 |
| 206 | SRI_1_00110 | SRI | -0.62 | 0.03 |
| 207 | OPN1MW_1_000011 | OPN1MW | -0.62 | 0.02 |
| 208 | OPN1MW2_1_000011 | OPN1MW2 | -0.62 | 0.02 |
| 209 | SIRT5_1_11000 | SIRT5 | -0.62 | 0.03 |
| 210 | STAMBPL1_1_11101 | STAMBPL1 | -0.62 | 0.01 |
| 211 | DRD2_1_10001 | DRD2 | -0.62 | 0.01 |
| 212 | CCDC12_1_111 | CCDC12 | -0.61 | 0.04 |
| 213 | ITGA3_1_11111 | ITGA3 | -0.61 | 0.02 |
| 214 | DDHD2_1_11111 | DDHD2 | -0.61 | 0.01 |
| 215 | FGG_1_10111 | FGG | -0.61 | 0.02 |
| 216 | POFUT2_1_1110 | POFUT2 | -0.61 | 0.02 |
| 217 | KRT7_1_00100011 | KRT7 | -0.61 | 0.03 |
| 218 | LPPR1_1_10010 | LPPR1 | -0.61 | 0.02 |
| 219 | KIR3DX1_1_1011 | KIR3DX1 | -0.61 | 0.03 |
| 220 | PLA2G2E_1_10011 | PLA2G2E | -0.61 | 0.02 |
| 221 | TMX3_1_1100 | TMX3 | -0.60 | 0.03 |
| 222 | OR52L1_1_0011 | OR52L1 | -0.60 | 0.02 |
| 223 | SIRT7_1_1110 | SIRT7 | -0.60 | 0.02 |
| 224 | CCND2_1_00111 | CCND2 | -0.60 | 0.03 |
| 225 | ARPC1B_1_1100 | ARPC1B | -0.60 | 0.02 |
| 226 | GGT1_1_1010001110 | GGT1 | -0.60 | 0.02 |
| 227 | SRPRB_1_1101 | SRPRB | -0.60 | 0.03 |
| 228 | IFNA21_1_001101000 | IFNA21 | -0.60 | 0.03 |
| 229 | AKR1E2_1_00111 | AKR1E2 | -0.60 | 0.02 |
| 230 | TUBB4A_1_10110 | TUBB4A | -0.60 | 0.03 |
| 231 | KIAA0430_1_10100 | KIAA0430 | -0.60 | 0.02 |
| 232 | LOC399753_1_0010111 | LOC399753 | -0.60 | 0.01 |
| 233 | FANCD2_2_00110 | FANCD2 | -0.60 | 0.03 |
| 234 | CHI3L1_1_00011 | CHI3L1 | -0.60 | 0.02 |
| 235 | SLC10A6_1_01001 | SLC10A6 | -0.60 | 0.01 |
| 236 | TLE3_1_01111 | TLE3 | -0.60 | 0.02 |
| 237 | POLD1_1_11 | POLD1 | -0.59 | 0.02 |
| 238 | POLA2_1_11001 | POLA2 | -0.59 | 0.02 |
| 239 | NUDT13_1_111 | NUDT13 | -0.59 | 0.01 |
| 240 | HK3_1_11111 | HK3 | -0.59 | 0.03 |
| 241 | TIMP3_1_01110 | TIMP3 | -0.59 | 0.02 |
| 242 | SEC24D_1_1011 | SEC24D | -0.59 | 0.02 |
| 243 | PITPNM1_1_1001 | PITPNM1 | -0.59 | 0.03 |
| 244 | FGF3_1_0111 | FGF3 | -0.59 | 0.04 |
| 245 | SLC2A1_1_1111 | SLC2A1 | -0.59 | 0.03 |
| 246 | HCAR2_1_010011 | HCAR2 | -0.58 | 0.03 |
| 247 | CCR1_1_00011 | CCR1 | -0.58 | 0.04 |
| 248 | LOC442075_1_1101 | LOC442075 | -0.58 | 0.02 |
| 249 | MDM2_1_1111 | MDM2 | -0.58 | 0.05 |
| 250 | PSKH2_1_10011 | PSKH2 | -0.58 | 0.02 |
| 251 | LZIC_1_00101 | LZIC | -0.58 | 0.02 |
| 252 | TWF1_1_1101 | TWF1 | -0.58 | 0.02 |
| 253 | CALB1_1_11110 | CALB1 | -0.58 | 0.02 |
| 254 | HNRNPA3_1_011111001 | HNRNPA3 | -0.58 | 0.02 |
| 255 | SENP5_1_01111 | SENP5 | -0.58 | 0.02 |
| 256 | OR1B1_1_01010 | OR1B1 | -0.58 | 0.03 |
| 257 | MXRA5_1_110 | MXRA5 | -0.58 | 0.02 |
| 258 | USP17L3_1_1000100 | USP17L3 | -0.58 | 0.04 |
| 259 | USP17L1P_1_10000100 | USP17L1P | -0.58 | 0.04 |
| 260 | SQRDL_1_1111 | SQRDL | -0.58 | 0.04 |
| 261 | TNFRSF17_1_01011 | TNFRSF17 | -0.57 | 0.03 |
| 262 | KISS1R_1_1101 | KISS1R | -0.57 | 0.03 |
| 263 | ZNF101_1_10011 | ZNF101 | -0.57 | 0.02 |
| 264 | ST13_2_1000001 | ST13 | -0.57 | 0.03 |
| 265 | ST13P4_1_10001 | ST13P4 | -0.57 | 0.03 |
| 266 | KCTD8_1_111 | KCTD8 | -0.57 | 0.02 |
| 267 | PMF1_1_111 | PMF1 | -0.57 | 0.03 |
| 268 | P2RY10_1_1011 | P2RY10 | -0.57 | 0.04 |
| 269 | MS4A8B_1_10011 | MS4A8B | -0.57 | 0.03 |
| 270 | RAPSN_1_01010 | RAPSN | -0.57 | 0.03 |
| 271 | ATM_1_111010001 | ATM | -0.57 | 0.03 |
| 272 | SNAPIN_1_10100 | SNAPIN | -0.57 | 0.02 |
| 273 | SNAPC2_1_11111 | SNAPC2 | -0.57 | 0.02 |
| 274 | NDUFA1_1_11111 | NDUFA1 | -0.57 | 0.03 |
| 275 | TFPI_1_00110 | TFPI | -0.57 | 0.03 |
| 276 | EGFL7_1_11111 | EGFL7 | -0.57 | 0.03 |
| 277 | ZNF322_2_1000000110 | ZNF322 | -0.56 | 0.02 |
| 278 | ZNF322P1_2_100000110 | ZNF322P1 | -0.56 | 0.02 |
| 279 | LRRFIP2_1_01001 | LRRFIP2 | -0.56 | 0.04 |
| 280 | INSR_1_111101111 | INSR | -0.56 | 0.03 |
| 281 | ECT2_1_1111 | ECT2 | -0.56 | 0.03 |
| 282 | H2BFM_1_00011 | H2BFM | -0.56 | 0.04 |
| 283 | SLC13A3_1_0011 | SLC13A3 | -0.56 | 0.03 |
| 284 | GAMT_1_01011 | GAMT | -0.56 | 0.03 |
| 285 | BRWD1_1_01001 | BRWD1 | -0.56 | 0.03 |
| 286 | RAB23_1_01001 | RAB23 | -0.55 | 0.04 |
| 287 | CKLF-CMTM1_1_111 | CKLF-CMTM1 | -0.55 | 0.03 |
| 288 | LYVE1_1_00111 | LYVE1 | -0.55 | 0.02 |
| 289 | AWAT2_1_11011 | AWAT2 | -0.55 | 0.03 |
| 290 | GSTP1_1_1010 | GSTP1 | -0.55 | 0.01 |
| 291 | EFCAB4B_1_01011 | EFCAB4B | -0.55 | 0.03 |
| 292 | MVD_1_111 | MVD | -0.55 | 0.02 |
| 293 | PISD_1_1101 | PISD | -0.55 | 0.04 |
| 294 | TADA3_1_10111 | TADA3 | -0.55 | 0.02 |
| 295 | LIMK2_1_110010 | LIMK2 | -0.55 | 0.03 |
| 296 | EZH2_1_00101 | EZH2 | -0.55 | 0.03 |
| 297 | ALDH1B1_1_111 | ALDH1B1 | -0.55 | 0.04 |
| 298 | INSRR_1_0011 | INSRR | -0.55 | 0.04 |
| 299 | RALBP1_1_10101 | RALBP1 | -0.55 | 0.04 |
| 300 | YPEL2_1_11010 | YPEL2 | -0.55 | 0.03 |
| 301 | C19orf48_1_11111 | C19orf48 | -0.55 | 0.03 |
| 302 | MEF2BNB-MEF2B_1_0111 | MEF2BNB-MEF2B | -0.55 | 0.02 |
| 303 | MEF2B_1_0111 | MEF2B | -0.55 | 0.02 |
| 304 | ARF5_1_11101 | ARF5 | -0.55 | 0.04 |
| 305 | ALDH7A1_1_11001 | ALDH7A1 | -0.55 | 0.02 |
| 306 | TAF8_1_0111 | TAF8 | -0.55 | 0.05 |
| 307 | YWHAB_1_0111 | YWHAB | -0.54 | 0.03 |
| 308 | TREML2_1_1101 | TREML2 | -0.54 | 0.04 |
| 309 | NIT1_1_001101 | NIT1 | -0.54 | 0.03 |
| 310 | PLS3_1_01010 | PLS3 | -0.54 | 0.03 |
| 311 | ZNF124_1_10110 | ZNF124 | -0.54 | 0.04 |
| 312 | AMY1C_1_00110111111 | AMY1C | -0.54 | 0.04 |
| 313 | AMY1A_1_00110111111 | AMY1A | -0.54 | 0.04 |
| 314 | AMY1B_1_00110111111 | AMY1B | -0.54 | 0.04 |
| 315 | PCDHB4_1_01001 | PCDHB4 | -0.54 | 0.03 |
| 316 | ESX1_1_01111 | ESX1 | -0.54 | 0.03 |
| 317 | OSTBETA_1_10101 | OSTBETA | -0.54 | 0.04 |
| 318 | RNF144B_1_0110 | RNF144B | -0.54 | 0.04 |
| 319 | LOC100287178_1_001001001 | LOC100287178 | -0.54 | 0.05 |
| 320 | LOC100287238_1_001000101 | LOC100287238 | -0.54 | 0.05 |
| 321 | LOC728419_1_001001001 | LOC728419 | -0.54 | 0.05 |
| 322 | LOC100287205_1_001001001 | LOC100287205 | -0.54 | 0.05 |
| 323 | LOC728379_1_001001001 | LOC728379 | -0.54 | 0.05 |
| 324 | USP17L5_1_001001001 | USP17L5 | -0.54 | 0.05 |
| 325 | LOC728400_1_001001001 | LOC728400 | -0.54 | 0.05 |
| 326 | LOC100287513_1_001001001 | LOC100287513 | -0.54 | 0.05 |
| 327 | LOC100287327_1_0010001001 | LOC100287327 | -0.54 | 0.05 |
| 328 | LOC100287364_1_001001001 | LOC100287364 | -0.54 | 0.05 |
| 329 | LOC728373_1_001001001 | LOC728373 | -0.54 | 0.05 |
| 330 | USP17_1_001001001 | USP17 | -0.54 | 0.05 |
| 331 | LOC728405_1_001001001 | LOC728405 | -0.54 | 0.05 |
| 332 | LOC100287478_1_001001001 | LOC100287478 | -0.54 | 0.05 |
| 333 | LOC100288520_1_001001001 | LOC100288520 | -0.54 | 0.05 |
| 334 | LOC100287441_1_001001001 | LOC100287441 | -0.54 | 0.05 |
| 335 | LOC100287404_1_001001001 | LOC100287404 | -0.54 | 0.05 |
| 336 | LOC728369_1_001001001 | LOC728369 | -0.54 | 0.05 |
| 337 | LOC728393_1_001001001 | LOC728393 | -0.54 | 0.05 |
| 338 | SLC25A20_1_11000 | SLC25A20 | -0.54 | 0.04 |
| 339 | ABP1_1_0101 | ABP1 | -0.54 | 0.04 |
| 340 | CAPN5_1_11001 | CAPN5 | -0.54 | 0.04 |
| 341 | NDUFV2_1_11110 | NDUFV2 | -0.54 | 0.04 |
| 342 | ZNF702P_1_0101101 | ZNF702P | -0.54 | 0.03 |
| 343 | CYP51A1_1_10011 | CYP51A1 | -0.54 | 0.04 |
| 344 | KCNJ11_1_11111 | KCNJ11 | -0.54 | 0.03 |
| 345 | GNAI2_1_10101 | GNAI2 | -0.54 | 0.04 |
| 346 | MT1F_1_11101 | MT1F | -0.54 | 0.03 |
| 347 | TNK2_1_0111 | TNK2 | -0.54 | 0.04 |
| 348 | ADAMTS6_1_01100111 | ADAMTS6 | -0.54 | 0.05 |
| 349 | IDH3G_1_10111 | IDH3G | -0.54 | 0.05 |
| 350 | SKIL_1_1110 | SKIL | -0.54 | 0.04 |
| 351 | ABAT_1_0110 | ABAT | -0.53 | 0.04 |
| 352 | RGS1_1_01001 | RGS1 | -0.53 | 0.04 |
| 353 | SGK223_1_1110 | SGK223 | -0.53 | 0.04 |
| 354 | VANGL1_1_1001 | VANGL1 | -0.53 | 0.04 |
| 355 | RHOA_1_11001 | RHOA | -0.53 | 0.04 |
| 356 | AIRE_1_01100 | AIRE | -0.53 | 0.04 |
| 357 | MAN1A1_1_00110 | MAN1A1 | -0.53 | 0.04 |
| 358 | S1PR5_1_1010 | S1PR5 | -0.53 | 0.04 |
| 359 | CNTRL_1_1011 | CNTRL | -0.53 | 0.04 |
| 360 | PCDHGB1_1_11111 | PCDHGB1 | -0.53 | 0.05 |
| 361 | PLK2_1_1011 | PLK2 | -0.53 | 0.05 |
| 362 | PDK2_1_11100 | PDK2 | -0.53 | 0.05 |
| 363 | HNRNPA1L2_1_101 | HNRNPA1L2 | -0.53 | 0.04 |
| 364 | IFNA17_1_011000101011 | IFNA17 | -0.53 | 0.03 |
| 365 | PRDM1_1_11011 | PRDM1 | -0.53 | 0.03 |
| 366 | NME3_1_0111 | NME3 | -0.53 | 0.03 |
| 367 | IMPAD1_1_1101 | IMPAD1 | -0.53 | 0.04 |
| 368 | STX5_2_10011 | STX5 | -0.53 | 0.04 |
| 369 | EVPL_1_111 | EVPL | -0.53 | 0.03 |
| 370 | LTBP4_1_0111 | LTBP4 | -0.52 | 0.05 |
| 371 | TGFBR2_1_000100101 | TGFBR2 | -0.52 | 0.04 |
| 372 | CRP_1_01111 | CRP | -0.52 | 0.03 |
| 373 | RING1_1_01011 | RING1 | -0.52 | 0.02 |
| 374 | PKHD1_1_10110 | PKHD1 | -0.52 | 0.05 |
| 375 | KRT8_1_0101111 | KRT8 | -0.52 | 0.05 |
| 376 | SPNS1_1_10011 | SPNS1 | -0.52 | 0.04 |
| 377 | PCDH11Y_2_00111001 | PCDH11Y | -0.52 | 0.03 |
| 378 | REG1B_1_111001 | REG1B | -0.52 | 0.04 |
| 379 | AGAP9_1_11000111 | AGAP9 | -0.52 | 0.04 |
| 380 | AGAP10_1_11000111 | AGAP10 | -0.52 | 0.04 |
| 381 | IFNA8_1_11101010 | IFNA8 | -0.52 | 0.05 |
| 382 | ZNF766_1_11110 | ZNF766 | -0.52 | 0.04 |
| 383 | TNFAIP8L2_1_111 | TNFAIP8L2 | -0.52 | 0.03 |
| 384 | ALG8_1_11100 | ALG8 | -0.52 | 0.04 |
| 385 | AGAP5_1_101000111 | AGAP5 | -0.52 | 0.05 |
| 386 | CACNA2D4_1_10001 | CACNA2D4 | -0.51 | 0.03 |
| 387 | ZNF35_1_01110 | ZNF35 | -0.51 | 0.05 |
| 388 | UQCRQ_1_1111 | UQCRQ | -0.51 | 0.04 |
| 389 | HNF4G_1_01010 | HNF4G | -0.51 | 0.05 |
| 390 | CHD6_1_10011 | CHD6 | -0.51 | 0.04 |
| 391 | PDHA2_1_10010 | PDHA2 | -0.51 | 0.04 |
| 392 | PAX9_1_10011 | PAX9 | -0.51 | 0.04 |
| 393 | FBXW2_1_110 | FBXW2 | -0.51 | 0.05 |
| 394 | UROD_1_01111 | UROD | -0.51 | 0.03 |
| 395 | GIMAP4_1_0111 | GIMAP4 | -0.51 | 0.03 |
| 396 | EVX1_1_11111 | EVX1 | -0.50 | 0.04 |
| 397 | ADAT3_2_10100 | ADAT3 | -0.50 | 0.04 |
| 398 | CALML4_1_0111 | CALML4 | -0.50 | 0.04 |
| 399 | SLC9A8_2_10100 | SLC9A8 | -0.50 | 0.04 |
| 400 | CCT2_1_1001 | CCT2 | -0.50 | 0.03 |
| 401 | TRIM7_1_1010 | TRIM7 | -0.50 | 0.05 |
| 402 | TXNDC17_1_00101 | TXNDC17 | -0.50 | 0.04 |
| 403 | OLFML2B_1_10101 | OLFML2B | -0.50 | 0.05 |
| 404 | SORCS2_1_11111 | SORCS2 | -0.50 | 0.03 |
| 405 | HCN1_1_11000 | HCN1 | -0.50 | 0.05 |
| 406 | ARSA_1_011111 | ARSA | -0.50 | 0.03 |
| 407 | DPYS_1_11010 | DPYS | -0.49 | 0.04 |
| 408 | SDR16C5_1_11011 | SDR16C5 | -0.49 | 0.03 |
| 409 | FOXD4L3_2_0000000011001011 | FOXD4L3 | -0.49 | 0.04 |
| 410 | FOXD4L6_2_0000000011001011 | FOXD4L6 | -0.49 | 0.04 |
| 411 | HOXB8_1_01111 | HOXB8 | -0.49 | 0.05 |
| 412 | LCK_1_1101010 | LCK | -0.49 | 0.04 |
| 413 | ATN1_1_00111 | ATN1 | -0.49 | 0.03 |
| 414 | MAGI1_1_111 | MAGI1 | -0.49 | 0.05 |
| 415 | SIX6_1_00011 | SIX6 | -0.49 | 0.05 |
| 416 | TIGD6_1_00011 | TIGD6 | -0.49 | 0.05 |
| 417 | CYP17A1_1_10010 | CYP17A1 | -0.49 | 0.05 |
| 418 | DEGS2_1_10111 | DEGS2 | -0.48 | 0.04 |
| 419 | ZNF692_1_00011 | ZNF692 | -0.48 | 0.05 |
| 420 | PAICS_1_1011 | PAICS | -0.47 | 0.05 |
| 421 | IFT57_2_01010 | IFT57 | -0.47 | 0.05 |
| 422 | UCHL3_1_11011 | UCHL3 | -0.46 | 0.05 |
| 423 | OGFOD1_1_10111 | OGFOD1 | -0.46 | 0.05 |
| 424 | SNX29_1_1110 | SNX29 | -0.45 | 0.05 |
| 425 | ZNF79_1_1011 | ZNF79 | -0.43 | 0.05 |
